# Supplementary material for: Rapid protein assignments and structures from raw NMR spectra with the deep learning technique ARTINA
Source: Nat Commun. 2022 Oct 18;13:6151. doi: 10.1038/s41467-022-33879-5 (PMC9579175; doi:10.1038/s41467-022-33879-5)
Supplement: Supplementary file 1 — Supplementary Info File #1 [file 41467_2022_33879_MOESM1_ESM.pdf]

Supplementary Information

Rapid protein assignments and structures from raw NMR spectra with the deep learning technique ARTINA

Piotr Klukowski[1]*, Roland Riek[1]*, Peter Güntert[1,2,3]*

[1] Laboratory of Physical Chemistry, ETH Zurich, Vladimir-Prelog-Weg 2, 8093 Zurich, Switzerland

[2] Institute of Biophysical Chemistry, Goethe University Frankfurt, Max-von-Laue-Str. 9, 60438 Frankfurt am Main, Germany

[3] Department of Chemistry, Tokyo Metropolitan University, 1-1 Minami-Osawa, Hachioji, 192-0397 Tokyo, Japan

* e-mail: piotr.klukowski@phys.chem.ethz.ch; roland.riek@phys.chem.ethz.ch; peter.guentert@phys.chem.ethz.ch

Contents

Supplementary Figure 1 Examples of generated spectrum fragments for deconvolution model training

Supplementary Figure 2 Visualization of 100 protein structures determined automatically with ARTINA overlaid with corresponding PDB depositions

Supplementary Figure 3 Robustness of peak picking to background artefacts

Supplementary Figure 4 The three proteins with backbone RMSD > 3 Å between the automatically determined structure and the PDB deposition

Supplementary Table 1 Supported NMR spectrum types

Supplementary Table 2 Proteins and spectra used for automated structure determination experiments

Supplementary Table 3 Metadata for PDB reference structures

Supplementary Table 4 Results of automated structure determination of 100 proteins

Supplementary Table 5 Structure accuracy prediction

Supplementary Table 6 ANSURR structure evaluation scores

Supplementary Table 7 RPF structure evaluation scores

Supplementary Table 8 Consensus structure bundles

Supplementary Table 9 Results of restrained energy refinement

Supplementary Table 10 Chemical shift assignment accuracy of protein core residues

Supplementary Table 11 Quantitative analysis of sources of backbone RMSD in automatically determined structures

Supplementary Table 12 Quality metrics of 26 protein structures determined without and with 4D CC-NOESY spectra

Supplementary Table 13 Accuracy of automated chemical shift assignment using all input spectra

Supplementary Table 14 Accuracy of automated chemical shift assignment using all except NOESY-type input spectra

Supplementary Figure 1 Examples of generated spectrum fragments for deconvolution model training. Each row presents one generated spectrum fragment of size 64 × 32 × 5 that was used for the deconvolution model training. Ground truth positions of signal components are marked by red crosses.

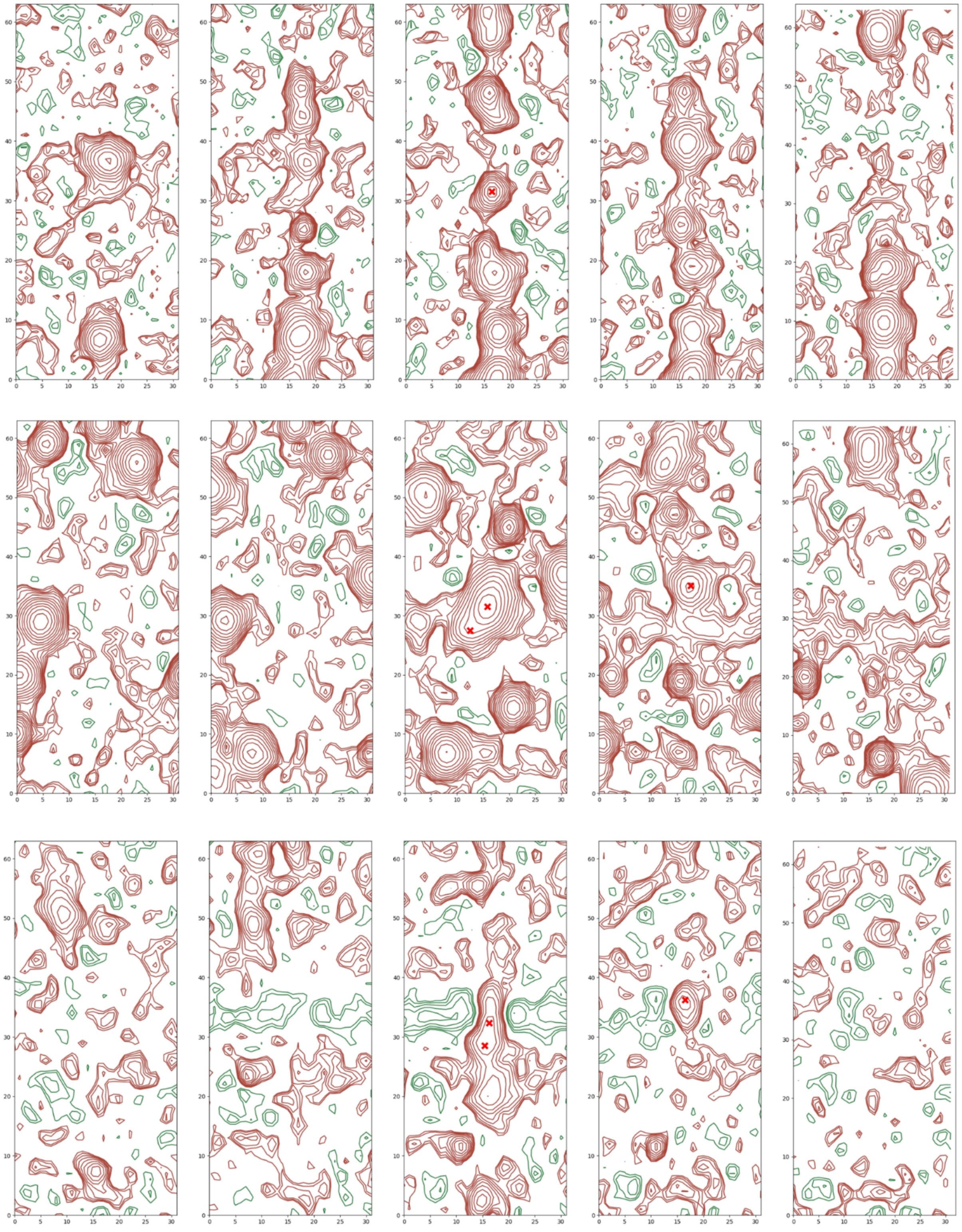

Supplementary Figure 2 Visualization of 100 protein structures determined with ARTINA (blue) overlaid with corresponding PDB depositions (orange). Each panel contains information about PDB code, RMSD between ARTINA structure and PDB deposition, and backbone/side-chain chemical shift assignment accuracy. Well-defined and disordered regions (Supplementary Table 4) are shown in and strong and light colors, respectively.

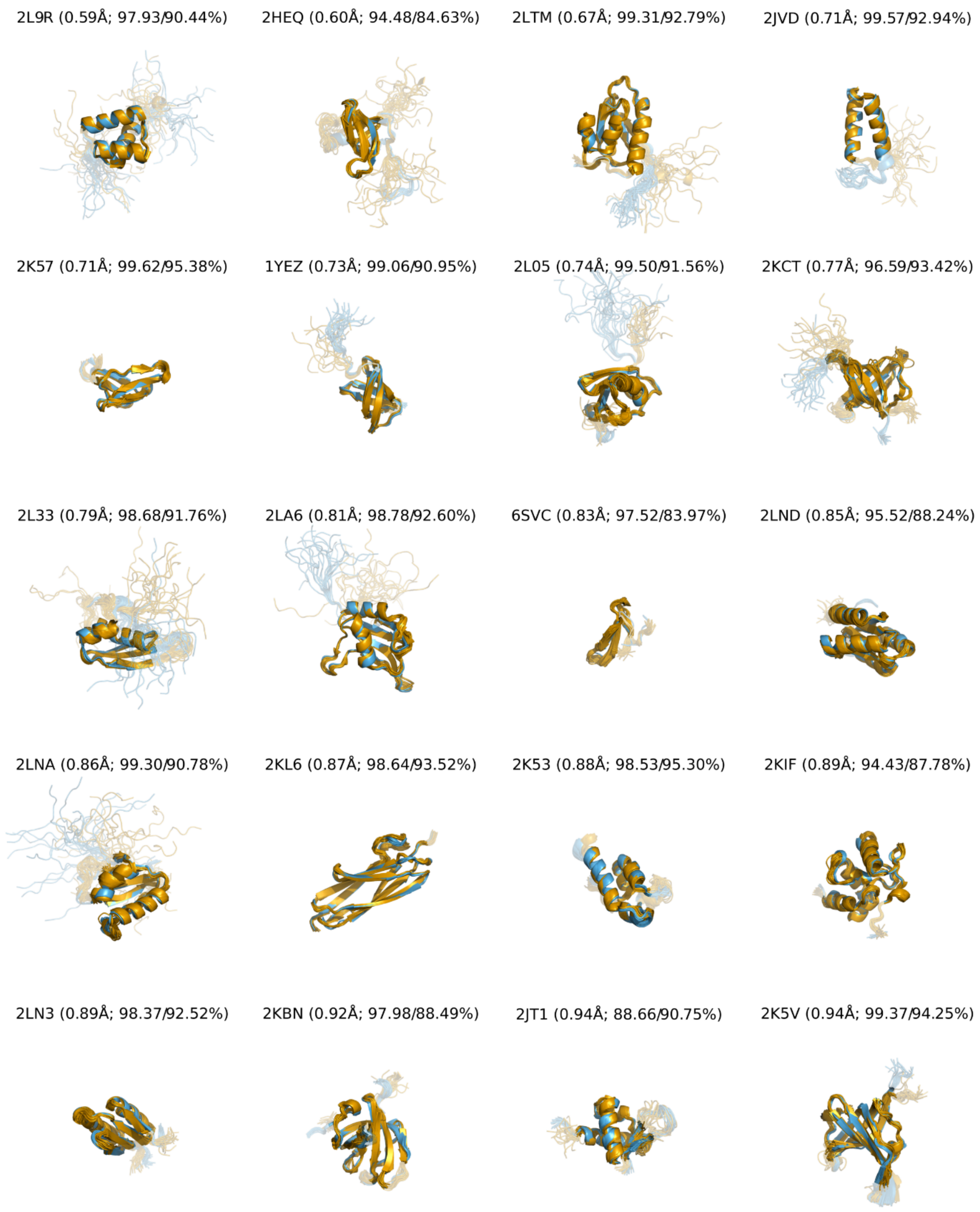

Supplementary Figure 2 (continued)

1VDY (0.95Å; 95.48/88.37%)

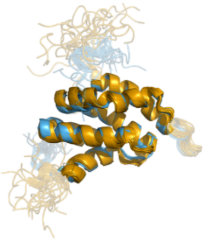

2KPN (0.97Å; 96.95/91.16%)

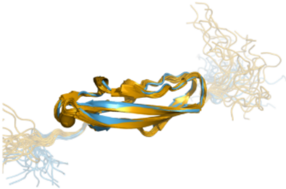

2MQL (0.98Å; 83.59/70.55%)

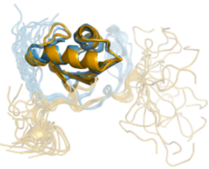

2K3A (0.99Å; 96.79/84.40%)

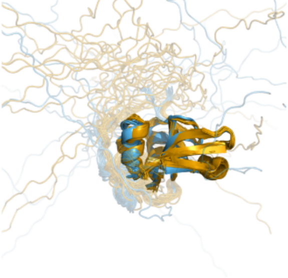

1WQU (0.99Å; 98.05/86.80%)

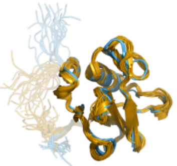

2K50 (1.00Å; 97.39/89.74%)

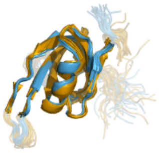

2KJR (1.02Å; 99.76/91.62%)

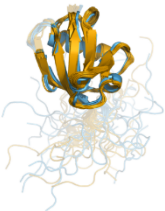

1VEE (1.03Å; 97.81/90.30%)

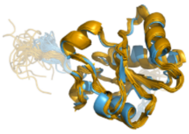

2K1G (1.05Å; 97.22/92.18%)

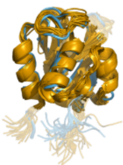

2L3B (1.05Å; 93.97/86.19%)

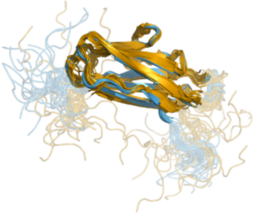

2M5O (1.08Å; 92.48/90.20%)

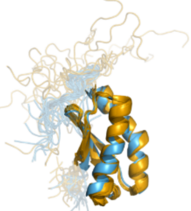

2K52 (1.10Å; 97.17/93.54%)

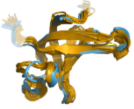

2M4F (1.11Å; 90.80/81.83%)

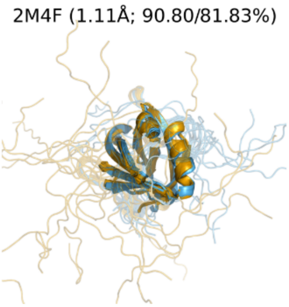

2MB0 (1.11Å; 97.69/88.46%)

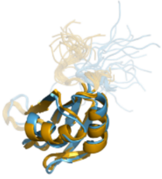

2N4B (1.14Å; 98.94/90.25%)

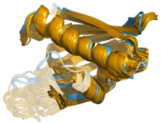

6SOW (1.16Å; 88.97/78.57%)

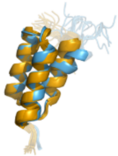

2LXU (1.19Å; 99.40/91.33%)

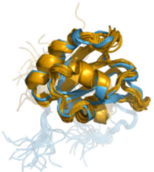

MDM2 (1.24Å; 98.08/84.88%)

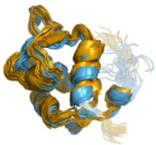

2KK8 (1.25Å; 99.18/90.26%)

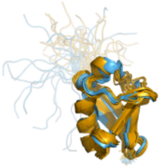

2KKL (1.26Å; 94.06/80.60%)

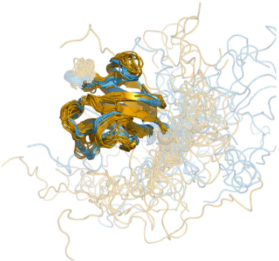

Supplementary Figure 2 (continued)

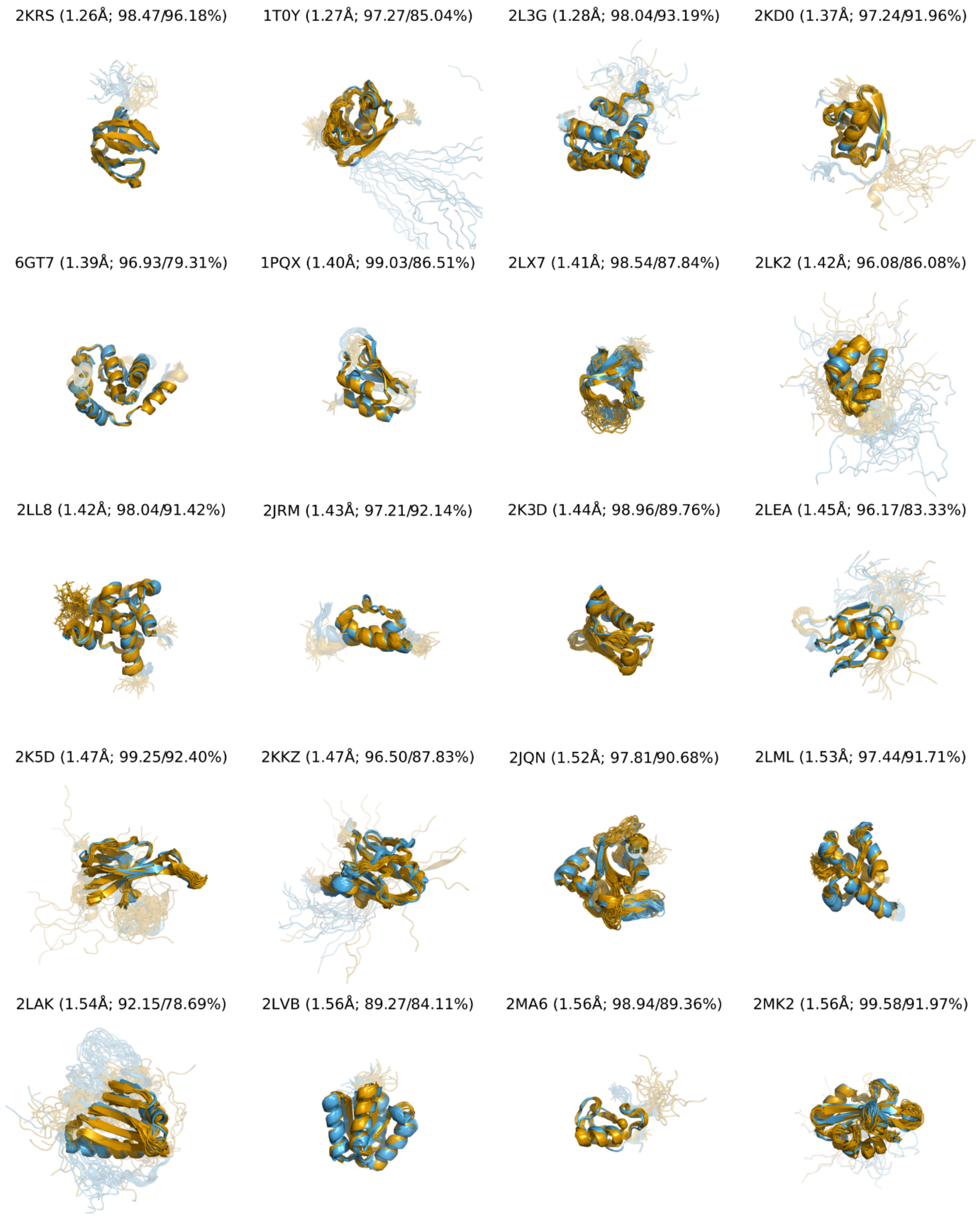

Supplementary Figure 2 (continued)

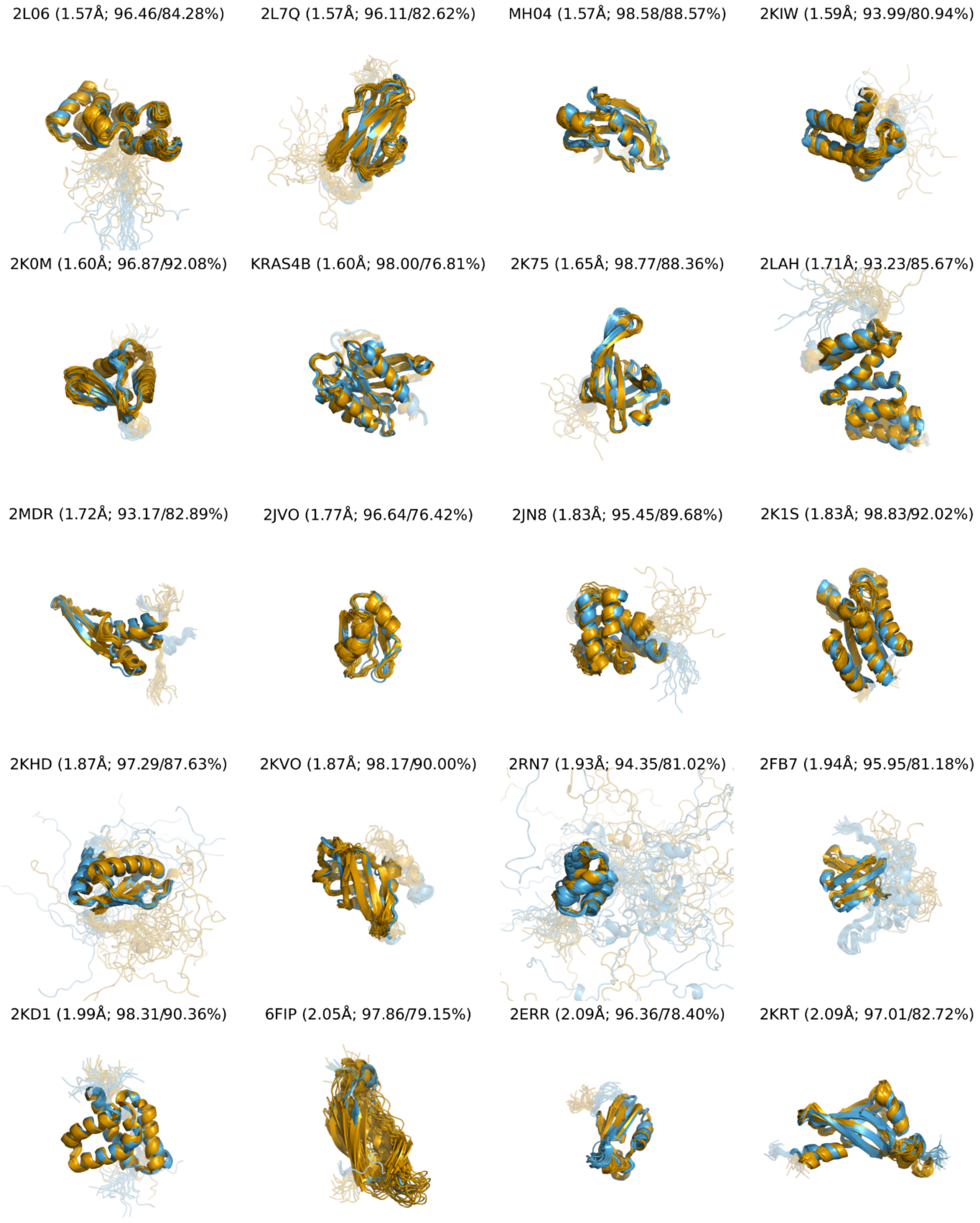

Supplementary Figure 2 (continued)

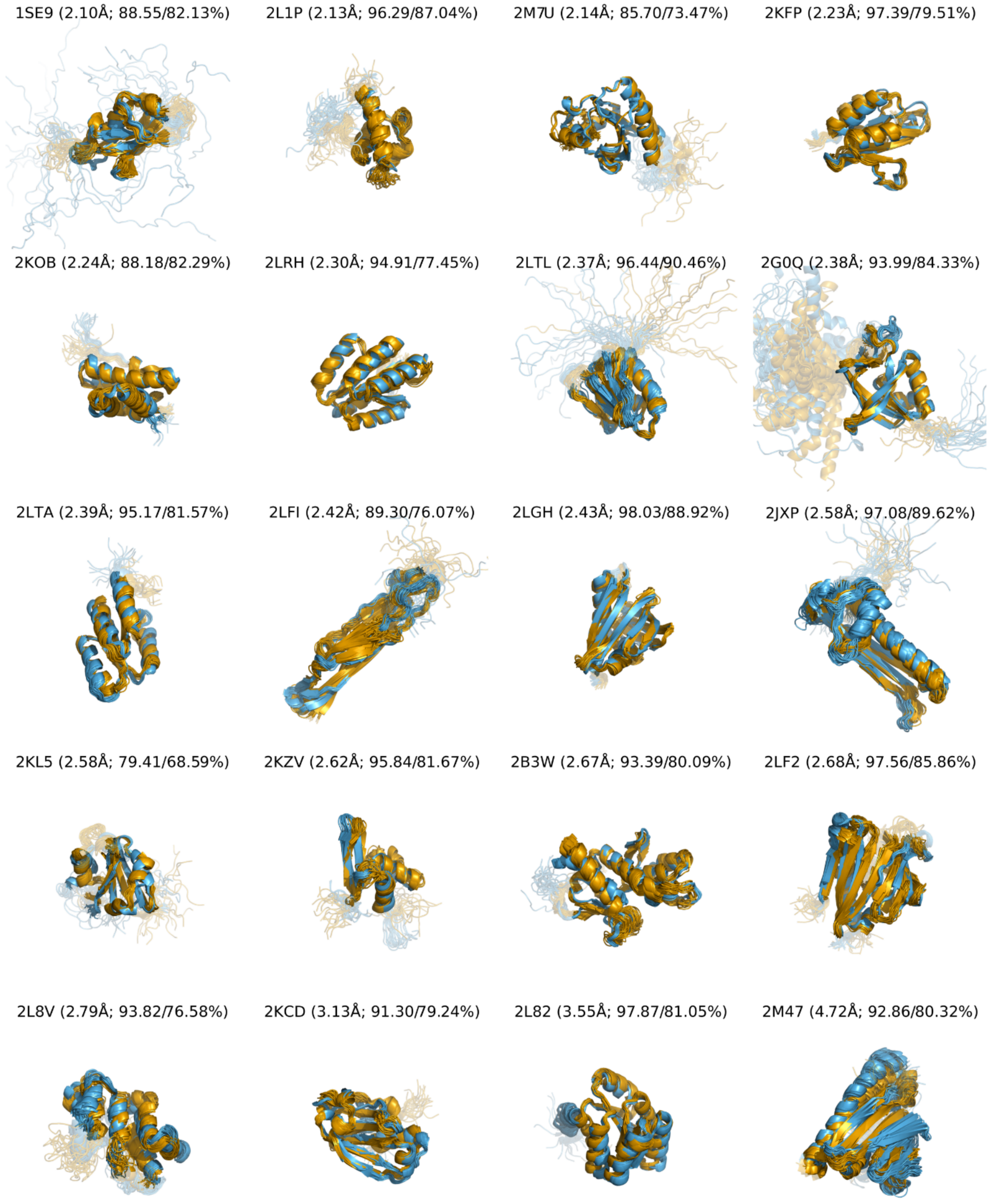

Supplementary Figure 3 Robustness of peak picking to background artefacts. A fragment of four consecutive layers of a ^{13}C-resolved [^{1}H, ^{1}H]-NOESY spectrum of a 20% ^{13}C-labelled protein of protein 6SOW[39] is shown. Final signal coordinates (after deconvolution) with classifier response > 0.5 are marked in black.

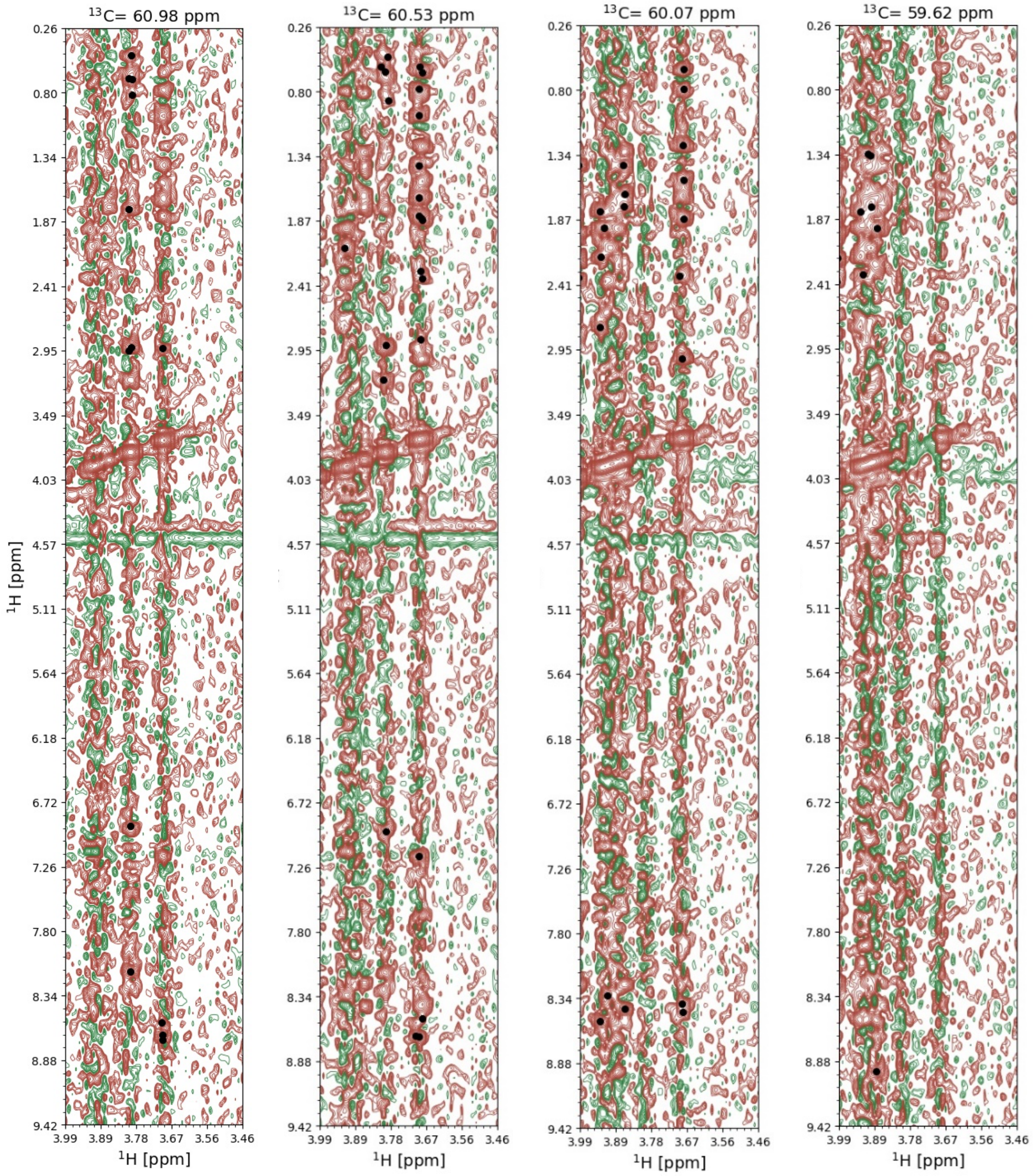

Supplementary Figure 4 The three proteins with backbone RMSD > 3 Å between the automatically determined structure (blue) and the PDB deposition (orange). **a** Protein 2KCD. The α-helix at residues 105–109 is displaced. **b** Protein 2L82. The α-helix at residues 138–153 is displaced. **c** Protein 2M47. The main reason for the high RMSD is a displacement of the last two α-helices at residues 111–157.

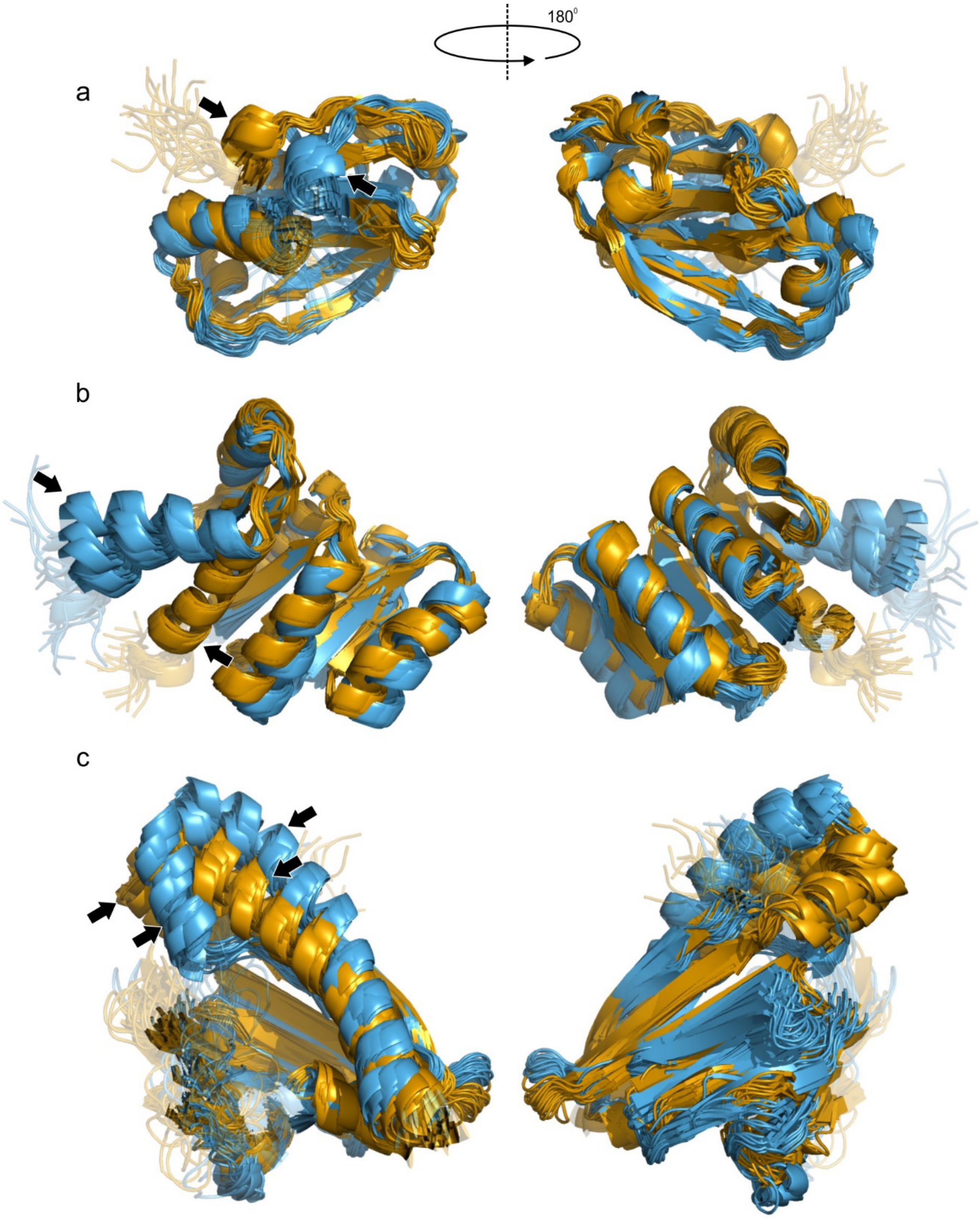

Supplementary Table 1 Supported NMR spectrum types.

**Spectrum category**	**Supported spectrum types**
2D	^{13}C-HSQC, ^{15}N-HSQC, NOESY (homonuclear), TOCSY (homonuclear), CACO, CBHEaro, CBHDaro
3D for backbone assignment	HNCA, HNHA, CBCANH, HNcoCA, HNCO, HNcaCO, CBCAcoNH, HBHAcoNH, CBCACOcaHA
3D for side-chain assignment	HCCH-TOCSY, ^{15}N-TOCSY, HCCH-COSY, CcoNH, HCcoNH, CCH-TOCSY
3D/4D NOESY	3D ^{13}C-resolved [^{1}H,^{1}H]-NOESY, 3D ^{15}N-resolved [^{1}H,^{1}H]-NOESY, 4D CC-NOESY

Further information about spectra can be provided to ARTINA by the use of tags. For instance, the ALI or ARO tags can be used to indicate that a ^{13}C-NOESY spectrum is expected to contain only aliphatic or aromatic signals. The tags POS and NEG can be used to restrict peak picking to positive and negative signals.

Supplementary Table 2 Proteins and spectra used for automated structure determination experiments.

ID	Protein	Residues	Number of spectra			
			Backbone	Side-chain	NOESY	Total
1	6SVC	35	2	1	3	6
2	2JVD	54	6	3	2	11
3	2K57	55	6	6	2	14
4	6SOW	58	5	2	3	10
5	2LX7	60	7	7	4	18
6	2MA6	61	5	7	4	16
7	2JRM	65	5	4	3	12
8	1YEZ	68	8	5	3	16
9	2L9R	69	5	1	3	9
10	2K52	74	7	4	3	14
11	2KRS	74	5	7	4	16
12	2K53	76	4	4	3	11
13	2JT1	77	6	1	3	10
14	2JVO	77	4	4	2	10
15	2ERR	81	3	2	2	7
16	2L1P	83	4	2	3	9
17	2LN3	83	5	3	3	11
18	2HEQ	84	6	3	4	13
19	2KK8	84	5	4	3	12
20	2KD0	85	6	4	3	13
21	2LML	86	6	5	4	15
22	2K3D	87	6	2	4	12
23	2LK2	89	7	6	4	17
24	MH04	90	7	3	2	12
25	1PQX	91	4	5	3	12
26	2L33	91	4	3	3	10
27	2KZV	92	7	6	4	17
28	2KCT	94	6	6	3	15
29	2MDR	94	4	3	3	10
30	2FB7	95	5	4	2	11
31	2MB0	95	2	3	3	8
32	2L05	95	6	4	3	13
33	2KJR	95	5	4	3	12
34	2M5O	97	5	1	3	9
35	MDM2	97	3	2	2	7
36	2LNA	99	6	7	4	17
37	2LA6	99	5	2	3	10
38	6FIP	99	6	5	2	13
39	2LEA	100	4	3	3	10
40	2LL8	101	6	6	3	15
41	2KPN	103	7	4	3	14
42	2K0M	104	7	4	3	14
43	2K5V	104	8	4	3	15
44	2MQL	105	2	4	3	9
45	2K75	106	6	6	3	15
46	2LTM	107	5	3	3	11
47	2KOB	108	7	5	4	16
48	2KHD	108	7	7	3	17
49	2RN7	108	5	4	3	12
50	2LXU	108	5	5	4	14
51	2KIF	108	1	1	3	5
52	2KBN	109	6	5	4	15
53	2MK2	109	7	4	3	14
54	2K50	110	8	3	3	14
55	2KL5	110	3	2	3	8
56	2LTA	110	5	2	3	10
57	2KIW	111	7	7	4	18
58	2LVB	112	4	1	3	8
59	2LND	112	5	3	3	11
60	1WQU	114	8	6	2	16
61	2KL6	114	8	5	3	16
62	6GT7	115	5	3	3	11
63	2JN8	115	7	8	3	18
64	2K5D	116	7	4	3	14
65	2KD1	118	6	4	3	13
66	2LTL	119	4	3	3	10
67	2KVO	120	7	7	4	18
68	1T0Y	120	6	5	3	14
69	2KCD	120	8	6	5	19
70	2KRT	121	7	5	3	15
71	2LFI	122	5	5	3	13
72	2JQN	122	5	3	3	11
73	2L7Q	124	6	4	3	13
74	2KFP	125	7	6	4	17
75	1SE9	126	6	3	3	12
76	2L3G	126	5	3	3	11
77	2L3B	130	7	4	4	15
78	2LRH	134	5	3	3	11
79	1VEE	134	8	5	3	16
80	2K1G	136	7	7	3	17
81	2KKZ	140	7	5	3	15
82	1VDY	140	8	7	2	17
83	2KKL	140	7	7	4	18
84	2N4B	142	6	5	4	15
85	2L8V	143	7	4	4	15
86	2LGH	144	7	6	4	17
87	2K1S	149	6	6	4	16
88	2M4F	151	8	6	2	16
89	2JXP	155	8	7	2	17
90	2L06	155	6	6	3	15
91	2LAH	160	4	3	3	10
92	2LAK	160	7	6	4	17
93	2L82	162	4	2	3	9
94	2M47	163	7	7	3	17
95	2K3A	163	8	6	3	17
96	2M7U	165	5	3	3	11
97	2B3W	168	8	7	5	20
98	KRAS4B	169	6	5	3	14
99	2G0Q	173	7	2	3	12
100	2LF2	175	7	7	4	18

Supplementary Table 3 Metadata for PDB reference structures. Extracted from PDB entries.

**Protein number**	1
**PDB code**	6SVC, doi:10.2210/pdb6SVC/pdb
**BMRB code**	34432, doi:10.13018/BMR34432
**PDB Header**	PEPTIDE BINDING PROTEIN
**Protein name**	PROTEIN ALLOSTERY OF WW DOMAIN AT ATOMIC RESOLUTION: APO STRUCTURE
**Deposition date**	18.09.2019
**PDB title**	PROTEIN ALLOSTERY OF THE WW DOMAIN AT ATOMIC RESOLUTION: APO STRUCTURE
**PDB authors**	D.STROTZ, J.ORTS, M.FRIEDMANN, P.GUNTERT, B.VOGELI, R.RIEK
**Last author**	RIEK
**Reference**	ANGEW.CHEM.INT.ED.ENGL. 59, 22132 (2020), doi:10.1002/ANIE.202008734
**Reference authors**	D.STROTZ, J.ORTS, H.KADAVATH, M.FRIEDMANN, D.GHOSH, S.OLSSON, C.N.CHI, A.POKHARNA, P.GUNTERT, B.VOGELI, R.RIEK
**Reference title**	PROTEIN ALLOSTERY AT ATOMIC RESOLUTION
**Software listed**	CCPNMR, CYANA, NMRDRAW, NMRPIPE
**Spectrometer**	BRUKER (700 MHZ)

**Protein number**	2
**PDB code**	2JVD, doi:10.2210/pdb2JVD/pdb
**BMRB code**	15476, doi:10.13018/BMR15476
**PDB Header**	STRUCTURAL GENOMICS, UNKNOWN FUNCTION
**Protein name**	FOLDED N-TERMINAL FRAGMENT OF UPF0291 PROTEIN YNZC FROM BACILLUS SUBTILIS
**Deposition date**	18.09.2007
**PDB title**	SOLUTION NMR STRUCTURE OF THE FOLDED N-TERMINAL FRAGMENT OF UPF0291 PROTEIN YNZC FROM BACILLUS SUBTILIS. NORTHEAST STRUCTURAL GENOMICS TARGET SR384-1-46
**PDB authors**	J.M.ARAMINI, S.SHARMA, Y.J.HUANG, L.ZHAO, L.A.OWENS, K.STOKES, M.JIANG, R.XIAO, M.C.BARAN, G.V.T.SWAPNA, T.B.ACTON, G.T.MONTELIONE, NORTHEAST STRUCTURAL GENOMICS CONSORTIUM (NESG)
**Last author**	MONTELIONE
**Reference**	PROTEINS 72, 526 (2008), doi:10.1002/PROT.22064
**Reference authors**	J.M.ARAMINI, S.SHARMA, Y.J.HUANG, G.V.SWAPNA, C.K.HO, K.SHETTY, K.CUNNINGHAM, L.C.MA, L.ZHAO, L.A.OWENS, M.JIANG, R.XIAO, J.LIU, M.C.BARAN, T.B.ACTON, B.ROST, G.T.MONTELIONE
**Reference title**	SOLUTION NMR STRUCTURE OF THE SOS RESPONSE PROTEIN YNZC FROM BACILLUS SUBTILIS
**Software listed**	AUTOASSIGN, AUTOSTRUCTURE, CYANA, MOLPROBITY, NMRPIPE, PDBSTAT, PROCHECK, PSVS, SPARKY, TOPSPIN, VNMR
**Spectrometer**	BRUKER, VARIAN (800 MHZ, 600 MHZ)

**Protein number**	3
**PDB code**	2K57, doi:10.2210/pdb2K57/pdb
**BMRB code**	15825, doi:10.13018/BMR15825
**PDB Header**	STRUCTURAL GENOMICS, UNKNOWN FUNCTION
**Protein name**	PUTATIVE LIPOPROTEIN FROM PSEUDOMONAS SYRINGAE GENE LOCUS PSPTO2350
**Deposition date**	25.06.2008
**PDB title**	SOLUTION NMR STRUCTURE OF PUTATIVE LIPOPROTEIN FROM PSEUDOMONAS SYRINGAE GENE LOCUS PSPTO2350. NORTHEAST STRUCTURAL GENOMICS TARGET PSR76A
**PDB authors**	D.HANG, J.A.ARAMINI, P.ROSSI, D.WANG, M.JIANG, M.MAGLAQUI, R.XIAO, J.LIU, M.C.BARAN, T.B.ACTON, B.ROST, G.T.MONTELIONE, NORTHEAST STRUCTURAL GENOMICS CONSORTIUM (NESG)
**Last author**	MONTELIONE
**Reference**	
**Reference authors**	
**Reference title**	
**Software listed**	AUTOASSIGN, CNS, CYANA, MOLMOL, MOLPROBITY, NMRPIPE, PDBSTAT, PROCHECK, PSVS, RPF(AUTOSTRUCTURE), SPARKY, TALOS, TOPSPIN
**Spectrometer**	BRUKER, VARIAN (800 MHZ, 600 MHZ)

**Protein number**	4
**PDB code**	6SOW, doi:10.2210/pdb6SOW/pdb
**BMRB code**	34430, doi:10.13018/BMR34430
**PDB Header**	PROTEIN BINDING
**Protein name**	STAPHYLOCOCCAL PROTEIN A, C DOMAIN
**Deposition date**	30.08.2019
**PDB title**	NMR SOLUTION STRUCTURE OF STAPHYLOCOCCAL PROTEIN A, C DOMAIN
**PDB authors**	S.M.BACKLUND, H.IWAI
**Last author**	IWAI
**Reference**	MOLECULES 2, 6 (2021), doi:10.3390/MOLECULES26030747
**Reference authors**	H.A.HEIKKINEN, S.M.BACKLUND, H.IWAI
**Reference title**	NMR STRUCTURE DETERMINATIONS OF SMALL PROTEINS USING ONLY ONE FRACTIONALLY 20% 13 C- AND UNIFORMLY 100% 15 N-LABELED SAMPLE
**Software listed**	AMBER, CCPNMR, CYANA, PSVS, TALOS
**Spectrometer**	BRUKER (850 MHZ)

**Protein number**	5
**PDB code**	2LX7, doi:10.2210/pdb2LX7/pdb

**BMRB code**	18662, doi:10.13018/BMR18662
**PDB Header**	PROTEIN BINDING
**Protein name**	SH3 DOMAIN OF GROWTH ARREST-SPECIFIC PROTEIN 7 (GAS7) (FRAGMENT 1-60) FROM HOMO SAPIENS
**Deposition date**	15.08.2012
**PDB title**	SOLUTION NMR STRUCTURE OF SH3 DOMAIN OF GROWTH ARREST-SPECIFIC PROTEIN 7 (GAS7) (FRAGMENT 1-60) FROM HOMO SAPIENS, NORTHEAST STRUCTURAL GENOMICS CONSORTIUM (NESG) TARGET HR8574A
**PDB authors**	Y.YANG, T.A.RAMELOT, L.DAN, E.KOHAN, H.JANJUA, R.XIAO, T.ACTON, J.K.EVERETT, G.T.MONTELIONE, M.A.KENNEDY, NORTHEAST STRUCTURAL GENOMICS CONSORTIUM (NESG)
**Last author**	KENNEDY
**Reference**	
**Reference authors**	
**Reference title**	
**Software listed**	AUTOASSIGN, AUTOSTRUCTURE, CNS, CYANA, NMRPIPE, PINE, PSVS, SPARKY, TALOS+, TOPSPIN, VNMRJ
**Spectrometer**	BRUKER, VARIAN (850 MHZ, 600 MHZ)

**Protein number**	6
**PDB code**	2MA6, doi:10.2210/pdb2MA6/pdb
**BMRB code**	19329, doi:10.13018/BMR19329
**PDB Header**	LIGASE
**Protein name**	RING FINGER DOMAIN FROM KIP1 UBIQUITINATION-PROMOTING E3 COMPLEX PROTEIN 1 (KPC1/RNF123) FROM HOMO SAPIENS
**Deposition date**	28.06.2013
**PDB title**	SOLUTION NMR STRUCTURE OF THE RING FINGER DOMAIN FROM THE KIP1 UBIQUITINATION-PROMOTING E3 COMPLEX PROTEIN 1 (KPC1/RNF123) FROM HOMO SAPIENS, NORTHEAST STRUCTURAL GENOMICS CONSORTIUM (NESG) TARGET HR8700A
**PDB authors**	T.A.RAMELOT, Y.YANG, H.JANJUA, E.KOHAN, H.WANG, R.XIAO, T.B.ACTON, J.K.EVERETT, G.T.MONTELIONE, M.A.KENNEDY, NORTHEAST STRUCTURAL GENOMICS CONSORTIUM (NESG)
**Last author**	KENNEDY
**Reference**	
**Reference authors**	
**Reference title**	
**Software listed**	AUTOSTRUCTURE, CNS, CYANA, FMCGUI, NMRPIPE, PINE, PSVS, SPARKY, TALOS+, TOPSPIN, VNMRJ
**Spectrometer**	BRUKER, VARIAN (850 MHZ, 600 MHZ)

**Protein number**	7
**PDB code**	2JRM, doi:10.2210/pdb2JRM/pdb
**BMRB code**	15339, doi:10.13018/BMR15339
**PDB Header**	STRUCTURAL GENOMICS, UNKNOWN FUNCTION
**Protein name**	RIBOSOME MODULATION FACTOR VP1593 FROM VIBRIO PARAHAEMOLYTICUS
**Deposition date**	27.06.2007
**PDB title**	SOLUTION NMR STRUCTURE OF RIBOSOME MODULATION FACTOR VP1593 FROM VIBRIO PARAHAEMOLYTICUS. NORTHEAST STRUCTURAL GENOMICS TARGET VPR55
**PDB authors**	Y.TANG, P.ROSSI, G.SWAPNA, H.WANG, M.JIANG, K.CUNNINGHAM, L.OWENS, L.MA, R.XIAO, J.LIU, M.C.BARAN, T.B.ACTON, B.ROST, G.T.MONTELIONE, NORTHEAST STRUCTURAL GENOMICS CONSORTIUM (NESG)
**Last author**	MONTELIONE
**Reference**	
**Reference authors**	
**Reference title**	
**Software listed**	AUTOASSIGN, AUTOSTRUCTURE, CNS, CYANA, NMRPIPE, PDBSTAT, PSVS, SPARKY, TOPSPIN, X-PLOR
**Spectrometer**	BRUKER (800 MHZ, 600 MHZ)

**Protein number**	8
**PDB code**	1YEZ, doi:10.2210/pdb1YEZ/pdb
**BMRB code**	6505, doi:10.13018/BMR6505
**PDB Header**	STRUCTURAL GENOMICS, UNKNOWN FUNCTION
**Protein name**	CONSERVED PROTEIN FROM GENE LOCUS MM1357 OF METHANOSARCINA MAZEI
**Deposition date**	29.12.2004
**PDB title**	SOLUTION STRUCTURE OF THE CONSERVED PROTEIN FROM THE GENE LOCUS MM1357 OF METHANOSARCINA MAZEI. NORTHEAST STRUCTURAL GENOMICS TARGET MAR30
**PDB authors**	P.ROSSI, J.M.ARAMINI, G.V.T.SWAPNA, Y.P.HUANG, R.XIAO, C.K.HO, L.C.MA, T.B.ACTON, G.T.MONTELIONE, NORTHEAST STRUCTURAL GENOMICS CONSORTIUM (NESG)
**Last author**	MONTELIONE
**Reference**	
**Reference authors**	
**Reference title**	
**Software listed**	AUTOASSIGN, AUTOSTRUCTURE, NMRPIPE, SPARKY, VNMR, XWINNMR
**Spectrometer**	BRUKER, VARIAN (600 MHZ, 500 MHZ)

**Protein number**	9
**PDB code**	2L9R, doi:10.2210/pdb2L9R/pdb
**BMRB code**	17484, doi:10.13018/BMR17484
**PDB Header**	TRANSCRIPTION
**Protein name**	HOMEOBOX DOMAIN OF HOMEOBOX PROTEIN NKX-3.1 FROM HOMO SAPIENS

**Deposition date**	22.02.2011
**PDB title**	SOLUTION NMR STRUCTURE OF HOMEOBOX DOMAIN OF HOMEOBOX PROTEIN NKX-3.1 FROM HOMO SAPIENS, NORTHEAST STRUCTURAL GENOMICS CONSORTIUM TARGET HR6470A
**PDB authors**	G.LIU, R.XIAO, H.-W.LEE, K.HAMILTON, C.CICCOSANTI, H.B.WANG, T.B.ACTON, J.K.EVERETT, Y.J.HUANG, G.T.MONTELIONE, NORTHEAST STRUCTURAL GENOMICS CONSORTIUM (NESG)
**Last author**	MONTELIONE
**Reference**	
**Reference authors**	
**Reference title**	
**Software listed**	AUTOASSIGN, AUTOSTRUCTURE, CNS, CYANA, NMRPIPE, TALOS+, TOPSPIN, VNMRJ, XEASY
**Spectrometer**	BRUKER, VARIAN (800 MHZ, 600 MHZ)

**Protein number**	10
**PDB code**	2K52, doi:10.2210/pdb2K52/pdb
**BMRB code**	15821, doi:10.13018/BMR15821
**PDB Header**	STRUCTURAL GENOMICS, UNKNOWN FUNCTION
**Protein name**	UNCHARACTERIZED PROTEIN MJ1198 FROM METHANOCALDOCOCCUS JANNASCHII
**Deposition date**	24.06.2008
**PDB title**	STRUCTURE OF UNCHARACTERIZED PROTEIN MJ1198 FROM METHANOCALDOCOCCUS JANNASCHII. NORTHEAST STRUCTURAL GENOMICS TARGET MJR117B
**PDB authors**	P.ROSSI, M.MAGLAQUI, E.L.FOOTE, K.HAMILTON, C.CICCOSANTI, R.XIAO, R.NAIR, G.SWAPNA, J.K.EVERETT, T.B.ACTON, B.ROST, G.T.MONTELIONE, NORTHEAST STRUCTURAL GENOMICS CONSORTIUM (NESG)
**Last author**	MONTELIONE
**Reference**	
**Reference authors**	
**Reference title**	
**Software listed**	AUTOASSIGN, CNS, CYANA, MOLMOL, MOLPROBITY, NMRPIPE, PROCHECK, PSVS, RPF(AUTOSTRUCTURE), SPARKY, TALOS, TOPSPIN
**Spectrometer**	BRUKER (800 MHZ, 600 MHZ)

**Protein number**	11
**PDB code**	2KRS, doi:10.2210/pdb2KRS/pdb
**BMRB code**	16647, doi:10.13018/BMR16647
**PDB Header**	STRUCTURAL GENOMICS, UNKNOWN FUNCTION
**Protein name**	SH3 DOMAIN FROM CPF_0587 (FRAGMENT 415-479) FROM CLOSTRIDIUM PERFRINGENS
**Deposition date**	22.12.2009
**PDB title**	SOLUTION NMR STRUCTURE OF SH3 DOMAIN FROM CPF_0587 (FRAGMENT 415-479) FROM CLOSTRIDIUM PERFRINGENS. NORTHEAST STRUCTURAL GENOMICS CONSORTIUM (NESG) TARGET CPR74A
**PDB authors**	T.A.RAMELOT, J.R.CORT, M.MAGLAQUI, C.CICCOSANTI, H.JANJUA, R.NAIR, B.ROST, T.B.ACTON, R.XIAO, J.K.EVERETT, G.T.MONTELIONE, M.A.KENNEDY, NORTHEAST STRUCTURAL GENOMICS CONSORTIUM (NESG)
**Last author**	KENNEDY
**Reference**	
**Reference authors**	
**Reference title**	
**Software listed**	AUTOASSIGN, AUTOSTRUCTURE, CNS, NMRPIPE, PDBSTAT, PSVS, SPARKY, TOPSPIN, VNMR, X-PLOR
**Spectrometer**	BRUKER, VARIAN (850 MHZ, 600 MHZ)

**Protein number**	12
**PDB code**	2K53, doi:10.2210/pdb2K53/pdb
**BMRB code**	15822, doi:10.13018/BMR15822
**PDB Header**	STRUCTURAL GENOMICS, UNKNOWN FUNCTION
**Protein name**	A3DK08 PROTEIN FROM CLOSTRIDIUM THERMOCELLUM
**Deposition date**	24.06.2008
**PDB title**	NMR SOLUTION STRUCTURE OF A3DK08 PROTEIN FROM CLOSTRIDIUM THERMOCELLUM: NORTHEAST STRUCTURAL GENOMICS CONSORTIUM TARGET CMR9
**PDB authors**	G.V.T.SWAPNA, W.HUANG, M.JIANG, E.L.FOOTE, R.XIAO, R.NAIR, J.EVERETT, T.B.ACTON, B.ROST, G.T.MONTELIONE, NORTHEAST STRUCTURAL GENOMICS CONSORTIUM (NESG)
**Last author**	MONTELIONE
**Reference**	
**Reference authors**	
**Reference title**	
**Software listed**	AUTOASSIGN, AUTOSTRUCTURE, CNS, CYANA
**Spectrometer**	BRUKER, VARIAN (800 MHZ, 600 MHZ)

**Protein number**	13
**PDB code**	2JT1, doi:10.2210/pdb2JT1/pdb
**BMRB code**	15386, doi:10.13018/BMR15386
**PDB Header**	TRANSCRIPTION
**Protein name**	PEFI (PLASMID-ENCODED FIMBRIAE REGULATORY) PROTEIN FROM SALMONELLA TYPHIMURIUM
**Deposition date**	17.07.2007
**PDB title**	SOLUTION NMR STRUCTURE OF PEFI (PLASMID-ENCODED FIMBRIAE REGULATORY) PROTEIN FROM SALMONELLA TYPHIMURIUM. NORTHEAST STRUCTURAL GENOMICS TARGET STR82

**PDB authors**	J.M.ARAMINI, P.ROSSI, H.WANG, C.NWOSU, K.CUNNINGHAM, L.-C.MA, R.XIAO, J.LIU, M.C.BARAN, G.V.T.SWAPNA, T.B.ACTON, B.ROST, G.T.MONTELIONE, NORTHEAST STRUCTURAL GENOMICS CONSORTIUM (NESG)
**Last author**	MONTELIONE
**Reference**	PROTEINS 79, 335 (2011), doi:10.1002/PROT.22869
**Reference authors**	J.M.ARAMINI, P.ROSSI, J.R.CORT, L.C.MA, R.XIAO, T.B.ACTON, G.T.MONTELIONE
**Reference title**	SOLUTION NMR STRUCTURE OF THE PLASMID-ENCODED FIMBRIAE REGULATORY PROTEIN PEFI FROM SALMONELLA ENTERICA SEROVAR TYPHIMURIUM
**Software listed**	AUTOASSIGN, AUTOSTRUCTURE, CNS, CYANA, MOLMOL, MOLPROBITY, NMRPIPE, PDBSTAT, PROCHECK, PSVS, SPARKY, TOPSPIN
**Spectrometer**	BRUKER (800 MHZ, 600 MHZ)

**Protein number**	14
**PDB code**	2JVO, doi:10.2210/pdb2JVO/pdb
**BMRB code**	15485, doi:10.13018/BMR15485
**PDB Header**	RNA BINDING PROTEIN
**Protein name**	SEGMENTAL ISOTOPE LABELING OF NPL3
**Deposition date**	24.09.2007
**PDB title**	SEGMENTAL ISOTOPE LABELING OF NPL3
**PDB authors**	L.SKRISOVSKA, F.H.-T.ALLAIN
**Last author**	ALLAIN
**Reference**	J.MOL.BIOL. 375, 151 (2008), doi:10.1016/J.JMB.2007.09.030
**Reference authors**	L.SKRISOVSKA, F.H.ALLAIN
**Reference title**	IMPROVED SEGMENTAL ISOTOPE LABELING METHODS FOR THE NMR STUDY OF MULTIDOMAIN OR LARGE PROTEINS: APPLICATION TO THE RRMS OF NPL3P AND HNRNP L
**Software listed**	ATNOS/CANDID, SPARKY, XWINNMR
**Spectrometer**	BRUKER (900 MHZ, 600 MHZ, 500 MHZ)

**Protein number**	15
**PDB code**	2ERR, doi:10.2210/pdb2ERR/pdb
**BMRB code**	6895, doi:10.13018/BMR6895
**PDB Header**	RNA BINDING PROTEIN
**Protein name**	RNA BINDING DOMAIN OF HUMAN FOX-1 IN COMPLEX WITH UGCAUGU
**Deposition date**	25.10.2005
**PDB title**	NMR STRUCTURE OF THE RNA BINDING DOMAIN OF HUMAN FOX-1 IN COMPLEX WITH UGCAUGU
**PDB authors**	F.H.ALLAIN, S.D.AUWETER
**Last author**	AUWETER
**Reference**	EMBO J. 25, 163 (2006), doi:10.1038/SJ.EMBOJ.7600918
**Reference authors**	S.D.AUWETER, R.FASAN, L.REYMOND, J.G.UNDERWOOD, D.L.BLACK, S.PITSCH, F.H.ALLAIN
**Reference title**	MOLECULAR BASIS OF RNA RECOGNITION BY THE HUMAN ALTERNATIVE SPLICING FACTOR FOX-1
**Software listed**	AMBER, CYANA
**Spectrometer**	BRUKER (900 MHZ, 600 MHZ, 500 MHZ)

**Protein number**	16
**PDB code**	2L1P, doi:10.2210/pdb2L1P/pdb
**BMRB code**	17092, doi:10.13018/BMR17092
**PDB Header**	DNA BINDING PROTEIN
**Protein name**	N-TERMINAL DOMAIN OF DNA-BINDING PROTEIN SATB1 FROM HOMO SAPIENS
**Deposition date**	02.08.2010
**PDB title**	NMR SOLUTION STRUCTURE OF THE N-TERMINAL DOMAIN OF DNA-BINDING PROTEIN SATB1 FROM HOMO SAPIENS: NORTHEAST STRUCTURAL GENOMICS TARGET HR4435B(179-250)
**PDB authors**	G.V.T.SWAPNA, A.F.MONTELIONE, R.SHASTRY, C.CICCOSANTI, H.JANJUA, R.XIAO, T.B.ACTON, J.K.EVERETT, G.T.MONTELIONE, NORTHEAST STRUCTURAL GENOMICS CONSORTIUM (NESG)
**Last author**	MONTELIONE
**Reference**	
**Reference authors**	
**Reference title**	
**Software listed**	AUTOASSIGN, AUTOSTRUCTURE, CNS, CYANA
**Spectrometer**	BRUKER (800 MHZ, 600 MHZ)

**Protein number**	17
**PDB code**	2LN3, doi:10.2210/pdb2LN3/pdb
**BMRB code**	18145, doi:10.13018/BMR18145
**PDB Header**	DE NOVO PROTEIN
**Protein name**	DE NOVO DESIGNED PROTEIN, IF3-LIKE FOLD
**Deposition date**	15.12.2011
**PDB title**	SOLUTION NMR STRUCTURE OF DE NOVO DESIGNED PROTEIN, IF3-LIKE FOLD, NORTHEAST STRUCTURAL GENOMICS CONSORTIUM TARGET OR135 (CASD TARGET)
**PDB authors**	G.LIU, R.KOGA, N.KOGA, R.XIAO, H.LEE, H.JANJUA, E.KOHAN, T.B.ACTON, J.K.EVERETT, D.BAKER, G.T.MONTELIONE, NORTHEAST STRUCTURAL GENOMICS CONSORTIUM (NESG)
**Last author**	MONTELIONE
**Reference**	NATURE 491, 222 (2012), doi:10.1038/NATURE11600
**Reference authors**	N.KOGA, R.TATSUMI-KOGA, G.LIU, R.XIAO, T.B.ACTON, G.T.MONTELIONE, D.BAKER
**Reference title**	PRINCIPLES FOR DESIGNING IDEAL PROTEIN STRUCTURES

**Software listed**	AUTOASSIGN, AUTOSTRUCTURE, CNS, CYANA, NMRPIPE, REDCAT, SPARKY, TALOS+, TOPSPIN, VNMRJ, XEASY
**Spectrometer**	BRUKER, VARIAN (800 MHZ, 600 MHZ)

**Protein number**	18
**PDB code**	2HEQ, doi:10.2210/pdb2HEQ/pdb
**BMRB code**	7175, doi:10.13018/BMR7175
**PDB Header**	STRUCTURAL GENOMICS, UNKNOWN FUNCTION
**Protein name**	BACILLUS SUBTILIS PROTEIN YORP
**Deposition date**	21.06.2006
**PDB title**	NMR STRUCTURE OF BACILLUS SUBTILIS PROTEIN YORP, NORTHEAST STRUCTURAL GENOMICS TARGET SR399
**PDB authors**	T.A.RAMELOT, J.R.CORT, D.WANG, H.JANJUA, K.CUNNINGHAM, L.-C.MA, R.XIAO, J.LIU, M.BARAN, G.V.T.SWAPNA, T.B.ACTON, B.ROST, G.M.MONTELIONE, M.A.KENNEDY, NORTHEAST STRUCTURAL GENOMICS CONSORTIUM (NESG)
**Last author**	KENNEDY
**Reference**	
**Reference authors**	
**Reference title**	
**Software listed**	AUTOSTRUCTURE, CNS, NMRPIPE, SPARKY, VNMR, X-PLOR_NIH
**Spectrometer**	VARIAN (750 MHZ, 600 MHZ)

**Protein number**	19
**PDB code**	2KK8, doi:10.2210/pdb2KK8/pdb
**BMRB code**	16355, doi:10.13018/BMR16355
**PDB Header**	STRUCTURAL GENOMICS, UNKNOWN FUNCTION
**Protein name**	A PUTATIVE UNCHARACTERIZED PROTEIN OBTAINED FROM ARABIDOPSIS THALIANA
**Deposition date**	16.06.2009
**PDB title**	NMR SOLUTION STRUCTURE OF A PUTATIVE UNCHARACTERIZED PROTEIN OBTAINED FROM ARABIDOPSIS THALIANA: NORTHEAST STRUCTURAL GENOMICS CONSORTIUM TARGET AR3449A
**PDB authors**	R.MANI, S.V.T.GURLA, R.SHASTRY, C.CICCOSANTI, E.FOOTE, M.JIANG, R.XIAO, R.NAIR, J.EVERETT, Y.HUANG, T.ACTON, B.ROST, G.T.MONTELIONE, NORTHEAST STRUCTURAL GENOMICS CONSORTIUM (NESG)
**Last author**	MONTELIONE
**Reference**	
**Reference authors**	
**Reference title**	
**Software listed**	AUTOASSIGN, AUTOSTRUCTURE, CNS, CYANA
**Spectrometer**	BRUKER, VARIAN (800 MHZ, 600 MHZ, 500 MHZ)

**Protein number**	20
**PDB code**	2KD0, doi:10.2210/pdb2KD0/pdb
**BMRB code**	16101, doi:10.13018/BMR16101
**PDB Header**	SIGNALING PROTEIN
**Protein name**	O64736 PROTEIN FROM ARABIDOPSIS THALIANA
**Deposition date**	31.12.2008
**PDB title**	NMR SOLUTION STRUCTURE OF O64736 PROTEIN FROM ARABIDOPSIS THALIANA. NORTHEAST STRUCTURAL GENOMICS CONSORTIUM MEGA TARGET AR3445A
**PDB authors**	G.V.T.SWAPNA, R.SHASTRY, E.FOOTE, C.CICCOSANTI, M.JIANG, R.XIAO, R.NAIR, J.EVERETT, Y.HUANG, T.B.ACTON, B.ROST, G.T.MONTELIONE, NORTHEAST STRUCTURAL GENOMICS CONSORTIUM (NESG)
**Last author**	MONTELIONE
**Reference**	
**Reference authors**	
**Reference title**	
**Software listed**	AUTOASSIGN, AUTOSTRUCTURE, CNS, CYANA, NMRPIPE, SPARKY
**Spectrometer**	BRUKER (800 MHZ, 600 MHZ)

**Protein number**	21
**PDB code**	2LML, doi:10.2210/pdb2LML/pdb
**BMRB code**	16860, doi:10.13018/BMR16860
**PDB Header**	TRANSPORT PROTEIN
**Protein name**	HOLO ACYL CARRIER PROTEIN FROM GEOBACTER METALLIREDUCENS REFINED WITH NH RDCS
**Deposition date**	05.12.2011
**PDB title**	SOLUTION NMR STRUCTURE OF HOLO ACYL CARRIER PROTEIN FROM GEOBACTER METALLIREDUCENS REFINED WITH NH RDCS, NORTHEAST STRUCTURAL GENOMICS CONSORTIUM TARGET GMR141
**PDB authors**	T.A.RAMELOT, M.J.SMOLA, H.LEE, L.ZHAO, C.CICCOSANTI, E.L.FOOTE, K.HAMILTON, R.NAIR, B.ROST, G.SWAPNA, T.B.ACTON, R.XIAO, J.K.EVERETT, J.H.PRESTEGARD, G.T.MONTELIONE, M.A.KENNEDY, NORTHEAST STRUCTURAL GENOMICS CONSORTIUM (NESG)
**Last author**	KENNEDY
**Reference**	BIOCHEMISTRY 50, 1442 (2011), doi:10.1021/BI101932S
**Reference authors**	T.A.RAMELOT, M.J.SMOLA, H.W.LEE, C.CICCOSANTI, K.HAMILTON, T.B.ACTON, R.XIAO, J.K.EVERETT, J.H.PRESTEGARD, G.T.MONTELIONE, M.A.KENNEDY
**Reference title**	SOLUTION STRUCTURE OF 4'-PHOSPHOPANTETHEINE - GMACP3 FROM GEOBACTER METALLIREDUCENS: A SPECIALIZED ACYL CARRIER PROTEIN WITH ATYPICAL STRUCTURAL FEATURES AND A PUTATIVE ROLE IN LIPOPOLYSACCHARIDE BIOSYNTHESIS
**Software listed**	AUTOASSIGN, AUTOSTRUCTURE, CNS, CYANA, FMCGUI, NMRPIPE, PDBSTAT, PINE_SERVER, PSVS, SPARKY, TOPSPIN, VNMR, X-PLOR_NIH
**Spectrometer**	BRUKER, VARIAN (850 MHZ, 600 MHZ)

**Protein number**	22
**PDB code**	2K3D, doi:10.2210/pdb2K3D/pdb
**BMRB code**	15750, doi:10.13018/BMR15750
**PDB Header**	STRUCTURAL GENOMICS, UNKNOWN FUNCTION
**Protein name**	FOLDED 79 RESIDUE FRAGMENT OF LIN0334 FROM LISTERIA INNOCUA
**Deposition date**	02.05.2008
**PDB title**	SOLUTION NMR STRUCTURE OF THE FOLDED 79 RESIDUE FRAGMENT OF LIN0334 FROM LISTERIA INNOCUA. NORTHEAST STRUCTURAL GENOMICS CONSORTIUM TARGET LKR15
**PDB authors**	T.A.RAMELOT, L.ZHAO, M.JIANG, E.L.FOOTE, R.XIAO, J.LIU, M.C.BARAN, G.V.T.SWAPNA, T.B.ACTON, B.ROST, G.T.MONTELIONE, M.A.KENNEDY, NORTHEAST STRUCTURAL GENOMICS CONSORTIUM (NESG)
**Last author**	KENNEDY
**Reference**	
**Reference authors**	
**Reference title**	
**Software listed**	AUTOASSIGN, AUTOSTRUCTURE, NMRPIPE, PSVS, SPARKY, TOPSPIN, VNMR, X-PLOR
**Spectrometer**	BRUKER, VARIAN (850 MHZ, 600 MHZ)

**Protein number**	23
**PDB code**	2LK2, doi:10.2210/pdb2LK2/pdb
**BMRB code**	17971, doi:10.13018/BMR17971
**PDB Header**	TRANSCRIPTION
**Protein name**	HOMEOBOX DOMAIN (171-248) OF HUMAN HOMEOBOX PROTEIN TGIF1
**Deposition date**	30.09.2011
**PDB title**	SOLUTION NMR STRUCTURE OF HOMEOBOX DOMAIN (171-248) OF HUMAN HOMEOBOX PROTEIN TGIF1, NORTHEAST STRUCTURAL GENOMICS CONSORTIUM TARGET HR4411B
**PDB authors**	Y.YANG, T.A.RAMELOT, J.R.CORT, R.SHASTRY, C.CICCOSANTI, K.HAMILTON, T.B.ACTON, R.XIAO, J.K.EVERETT, G.T.MONTELIONE, M.A.KENNEDY, NORTHEAST STRUCTURAL GENOMICS CONSORTIUM (NESG)
**Last author**	KENNEDY
**Reference**	
**Reference authors**	
**Reference title**	
**Software listed**	ASDP, AUTOASSIGN, CNS, CYANA, NMRPIPE, PDBSTAT, PINE_SERVER, PSVS, SPARKY, TOPSPIN, VNMR, X-PLOR_NIH
**Spectrometer**	BRUKER, VARIAN (850 MHZ, 600 MHZ)

**Protein number**	24
**PDB code**	(MH04)
**BMRB code**	
**PDB Header**	
**Protein name**	
**Deposition date**	
**PDB title**	
**PDB authors**	
**Last author**	
**Reference**	
**Reference authors**	
**Reference title**	
**Software listed**	
**Spectrometer**	

**Protein number**	25
**PDB code**	1PQX, doi:10.2210/pdb1PQX/pdb
**BMRB code**	5844, doi:10.13018/BMR5844
**PDB Header**	STRUCTURAL GENOMICS, UNKNOWN FUNCTION
**Protein name**	STAPHYLOCOCCUS AUREUS PROTEIN SAV1430
**Deposition date**	19.06.2003
**PDB title**	SOLUTION NMR STRUCTURE OF STAPHYLOCOCCUS AUREUS PROTEIN SAV1430. NORTHEAST STRUCTURAL GENOMICS CONSORTIUM TARGET ZR18
**PDB authors**	M.C.BARAN, J.M.ARAMINI, R.XIAO, Y.J.HUANG, T.B.ACTON, L.SHIH, G.T.MONTELIONE, NORTHEAST STRUCTURAL GENOMICS CONSORTIUM (NESG)
**Last author**	MONTELIONE
**Reference**	
**Reference authors**	
**Reference title**	
**Software listed**	AUTOASSIGN, AUTOPROC, AUTOSTRUCTURE, NMRPIPE, SPARKY, VNMR, X-PLOR
**Spectrometer**	VARIAN (600 MHZ, 500 MHZ)

**Protein number**	26
**PDB code**	2L33, doi:10.2210/pdb2L33/pdb
**BMRB code**	17169, doi:10.13018/BMR17169
**PDB Header**	TRANSCRIPTION REGULATOR
**Protein name**	DRBM 2 DOMAIN OF INTERLEUKIN ENHANCER- BINDING FACTOR 3 FROM HOMO SAPIENS
**Deposition date**	03.09.2010
**PDB title**	SOLUTION NMR STRUCTURE OF DRBM 2 DOMAIN OF INTERLEUKIN ENHANCER- BINDING FACTOR 3 FROM HOMO SAPIENS, NORTHEAST STRUCTURAL GENOMICS CONSORTIUM TARGET HR4527E

**PDB authors**	G.LIU, H.JANJUA, R.XIAO, T.B.ACTON, A.CICCOSANTI, R.B.SHASTRY, J.EVERETT, G.T.MONTELIONE, NORTHEAST STRUCTURAL GENOMICS CONSORTIUM, NORTHEAST STRUCTURAL GENOMICS CONSORTIUM (NESG)
**Last author**	MONTELIONE
**Reference**	
**Reference authors**	
**Reference title**	
**Software listed**	AUTOASSIGN, AUTOSTRUCTURE, CNS, CYANA, NMRPIPE, TALOS+, TOPSPIN, VNMRJ, XEASY
**Spectrometer**	BRUKER, VARIAN (800 MHZ, 600 MHZ)

**Protein number**	27
**PDB code**	2KZV, doi:10.2210/pdb2KZV/pdb
**BMRB code**	17020, doi:10.13018/BMR17020
**PDB Header**	STRUCTURAL GENOMICS, UNKNOWN FUNCTION
**Protein name**	CV_0373(175-257) PROTEIN FROM CHROMOBACTERIUM VIOLACEUM
**Deposition date**	25.06.2010
**PDB title**	SOLUTION NMR STRUCTURE OF CV_0373(175-257) PROTEIN FROM CHROMOBACTERIUM VIOLACEUM, NORTHEAST STRUCTURAL GENOMICS CONSORTIUM TARGET CVR118A
**PDB authors**	Y.YANG, T.A.RAMELOT, D.WANG, C.CICCOSANTI, L.MAO, H.JANJUA, T.B.ACTON, R.XIAO, J.K.EVERETT, G.T.MONTELIONE, M.A.KENNEDY, NORTHEAST STRUCTURAL GENOMICS CONSORTIUM (NESG)
**Last author**	KENNEDY
**Reference**	
**Reference authors**	
**Reference title**	
**Software listed**	AUTOASSIGN, AUTOSTRUCTURE, CNS, CYANA, NMRPIPE, PDBSTAT, PINE, PSVS, SPARKY, TOPSPIN, VNMR, X-PLOR
**Spectrometer**	BRUKER, VARIAN (850 MHZ, 600 MHZ)

**Protein number**	28
**PDB code**	2KCT, doi:10.2210/pdb2KCT/pdb
**BMRB code**	16096, doi:10.13018/BMR16096
**PDB Header**	CHAPERONE
**Protein name**	OB-FOLD DOMAIN OF HEME CHAPERONE CCME FROM DESULFOVIBRIO VULGARIS
**Deposition date**	29.12.2008
**PDB title**	SOLUTION NMR STRUCTURE OF THE OB-FOLD DOMAIN OF HEME CHAPERONE CCME FROM DESULFOVIBRIO VULGARIS. NORTHEAST STRUCTURAL GENOMICS TARGET DVR115G
**PDB authors**	J.M.ARAMINI, P.ROSSI, H.LEE, A.LEMAK, H.WANG, E.L.FOOTE, M.JIANG, R.XIAO, R.NAIR, G.V.T.SWAPNA, T.B.ACTON, B.ROST, J.K.EVERETT, G.T.MONTELIONE, NORTHEAST STRUCTURAL GENOMICS CONSORTIUM (NESG)
**Last author**	MONTELIONE
**Reference**	
**Reference authors**	
**Reference title**	
**Software listed**	AUTOASSIGN, AUTOSTRUCTURE, CNS, CYANA, NMRPIPE, PALES, PDBSTAT, PINE, PSVS, SPARKY, TOPSPIN, VNMRJ
**Spectrometer**	BRUKER, VARIAN (800 MHZ, 600 MHZ)

**Protein number**	29
**PDB code**	2MDR, doi:10.2210/pdb2MDR/pdb
**BMRB code**	19502, doi:10.13018/BMR19502
**PDB Header**	HYDROLASE
**Protein name**	THIRD DOUBLE-STRANDED RNA-BINDING DOMAIN (DSRBD3) OF HUMAN ADENOSINE-DEAMINASE ADAR1
**Deposition date**	17.09.2013
**PDB title**	SOLUTION STRUCTURE OF THE THIRD DOUBLE-STRANDED RNA-BINDING DOMAIN (DSRBD3) OF HUMAN ADENOSINE-DEAMINASE ADAR1
**PDB authors**	P.BARRAUD, S.BANERJEE, W.I.MOHAMED, M.F.JANTSCH, F.H.ALLAIN
**Last author**	ALLAIN
**Reference**	PROC.NATL.ACAD.SCI.USA 111, E1852 (2014), doi:10.1073/PNAS.1323698111
**Reference authors**	P.BARRAUD, S.BANERJEE, W.I.MOHAMED, M.F.JANTSCH, F.H.ALLAIN
**Reference title**	A BIMODULAR NUCLEAR LOCALIZATION SIGNAL ASSEMBLED VIA AN EXTENDED DOUBLE-STRANDED RNA-BINDING DOMAIN ACTS AS AN RNA-SENSING SIGNAL FOR TRANSPORTIN 1
**Software listed**	ATNOS, CING, CNS, CYANA, PROCHECKNMR, SPARKY, TOPSPIN
**Spectrometer**	BRUKER (900 MHZ, 750 MHZ, 700 MHZ, 600 MHZ, 500 MHZ)

**Protein number**	30
**PDB code**	2FB7, doi:10.2210/pdb2FB7/pdb
**BMRB code**	7084, doi:10.13018/BMR7084
**PDB Header**	STRUCTURAL GENOMICS, UNKNOWN FUNCTION
**Protein name**	PROTEIN FROM ZEBRA FISH DR.13312
**Deposition date**	08.12.2005
**PDB title**	NMR SOLUTION STRUCTURE OF PROTEIN FROM ZEBRA FISH DR.13312
**PDB authors**	R.C.TYLER, J.SONG, J.L.MARKLEY, CENTER FOR EUKARYOTIC STRUCTURAL GENOMICS (CESG)
**Last author**	MARKLEY
**Reference**	
**Reference authors**	
**Reference title**	
**Software listed**	ARIA, CNS, NMRPIPE, NMRVIEW

**Spectrometer**	VARIAN (600 MHZ)

**Protein number**	31
**PDB code**	2MB0, doi:10.2210/pdb2MB0/pdb
**BMRB code**	19382, doi:10.13018/BMR19382
**PDB Header**	SPLICING/RNA
**Protein name**	HNRNP G RRM IN COMPLEX WITH RNA 5'-AUCAAA-3'
**Deposition date**	22.07.2013
**PDB title**	SOLUTION STRUCTURE OF HNRNP G RRM IN COMPLEX WITH THE RNA 5'-AUCAAA-3'
**PDB authors**	A.MOURSY, F.H.-T.ALLAIN, A.CLERY
**Last author**	CLERY
**Reference**	NUCLEIC ACIDS RES. 42, 6659 (2014), doi:10.1093/NAR/GKU244
**Reference authors**	A.MOURSY, F.H.ALLAIN, A.CLERY
**Reference title**	CHARACTERIZATION OF THE RNA RECOGNITION MODE OF HNRNP G EXTENDS ITS ROLE IN SMN2 SPLICING REGULATION
**Software listed**	AMBER
**Spectrometer**	BRUKER (900 MHZ, 700 MHZ, 600 MHZ, 500 MHZ)

**Protein number**	32
**PDB code**	2L05, doi:10.2210/pdb2L05/pdb
**BMRB code**	17030, doi:10.13018/BMR17030
**PDB Header**	TRANSFERASE
**Protein name**	RAS-BINDING DOMAIN OF SERINE/THREONINE- PROTEIN KINASE B-RAF FROM HOMO SAPIENS
**Deposition date**	30.06.2010
**PDB title**	SOLUTION NMR STRUCTURE OF THE RAS-BINDING DOMAIN OF SERINE/THREONINE- PROTEIN KINASE B-RAF FROM HOMO SAPIENS, NORTHEAST STRUCTURAL GENOMICS CONSORTIUM TARGET HR4694F
**PDB authors**	J.M.ARAMINI, H.JANJUA, C.CICCOSANTI, R.SHASTRY, Y.J.HUANG, T.B.ACTON, R.XIAO, J.K.EVERETT, G.T.MONTELIONE, NORTHEAST STRUCTURAL GENOMICS CONSORTIUM (NESG)
**Last author**	MONTELIONE
**Reference**	
**Reference authors**	
**Reference title**	
**Software listed**	AUTOSTRUCTURE, CNS, CYANA, MOLPROBITY, NMRPIPE, PDBSTAT, PINE, PSVS, SPARKY, TALOS+, TOPSPIN
**Spectrometer**	BRUKER (800 MHZ, 600 MHZ)

**Protein number**	33
**PDB code**	2KJR, doi:10.2210/pdb2KJR/pdb
**BMRB code**	16338, doi:10.13018/BMR16338
**PDB Header**	CHAPERONE
**Protein name**	N-TERMINAL UBIQUITIN-LIKE DOMAIN FROM TUBULIN-BINDING COFACTOR B, CG11242, FROM DROSOPHILA MELANOGASTER
**Deposition date**	08.06.2009
**PDB title**	SOLUTION NMR STRUCTURE OF THE N-TERMINAL UBIQUITIN-LIKE DOMAIN FROM TUBULIN-BINDING COFACTOR B, CG11242, FROM DROSOPHILA MELANOGASTER. NORTHEAST STRUCTURAL GENOMICS CONSORTIUM TARGET FR629A (RESIDUES 8- 92)
**PDB authors**	T.A.RAMELOT, J.R.CORT, R.SHASTRY, C.CICCOSANTI, M.JIANG, R.NAIR, B.ROST, G.SWAPNA, T.B.ACTON, R.XIAO, J.K.EVERETT, G.T.MONTELIONE, M.A.KENNEDY, NORTHEAST STRUCTURAL GENOMICS CONSORTIUM (NESG)
**Last author**	KENNEDY
**Reference**	
**Reference authors**	
**Reference title**	
**Software listed**	AUTOASSIGN, AUTOSTRUCTURE, NMRPIPE, PDBSTAT, PSVS, SPARKY, TOPSPIN, VNMR, X-PLOR
**Spectrometer**	BRUKER, VARIAN (850 MHZ, 500 MHZ)

**Protein number**	34
**PDB code**	2M5O, doi:10.2210/pdb2M5O/pdb
**BMRB code**	19068, doi:10.13018/BMR19068
**PDB Header**	BIOSYNTHETIC PROTEIN
**Protein name**	SOLUTION NMR STRUCTURE CTD DOMAIN OF NFU1 IRON-SULFUR CLUSTER SCAFFOLD HOMOLOG FROM HOMO SAPIENS
**Deposition date**	01.03.2013
**PDB title**	SOLUTION NMR STRUCTURE CTD DOMAIN OF NFU1 IRON-SULFUR CLUSTER SCAFFOLD HOMOLOG FROM HOMO SAPIENS, NORTHEAST STRUCTURAL GENOMICS CONSORTIUM (NESG) TARGET HR2876C
**PDB authors**	G.LIU, R.XIAO, H.JANJUA, K.HAMILTON, R.SHASTRY, E.KOHAN, T.B.ACTON, J.K.EVERETT, K.PEDERSON, Y.J.HUANG, G.T.MONTELIONE, NORTHEAST STRUCTURAL GENOMICS CONSORTIUM (NESG), MITOCHONDRIAL PROTEIN PARTNERSHIP (MPP)
**Last author**	MONTELIONE
**Reference**	
**Reference authors**	
**Reference title**	
**Software listed**	AUTOASSIGN, AUTOSTRUCTURE, CNS, CYANA, NMRPIPE, PSVS, REDCAT, SPARKY, TALOS+, TOPSPIN, VNMRJ, XEASY
**Spectrometer**	BRUKER, VARIAN (800 MHZ, 600 MHZ)

**Protein number**	35
**PDB code**	(MDM2)
**BMRB code**	
**PDB Header**	
**Protein name**	
**Deposition date**	
**PDB title**	
**PDB authors**	
**Last author**	
**Reference**	
**Reference authors**	
**Reference title**	
**Software listed**	
**Spectrometer**	

**Protein number**	36
**PDB code**	2LNA, doi:10.2210/pdb2LNA/pdb
**BMRB code**	18156, doi:10.13018/BMR18156
**PDB Header**	HYDROLASE
**Protein name**	MITOCHONDRIAL INNER MEMBRANE DOMAIN (RESIDUES 164-251), FTSH_EXT, FROM PARAPLEGIN-LIKE PROTEIN AFG3L2 FROM HOMO SAPIENS
**Deposition date**	20.12.2011
**PDB title**	SOLUTION NMR STRUCTURE OF THE MITOCHONDRIAL INNER MEMBRANE DOMAIN (RESIDUES 164-251), FTSH_EXT, FROM THE PARAPLEGIN-LIKE PROTEIN AFG3L2 FROM HOMO SAPIENS, NORTHEAST STRUCTURAL GENOMICS CONSORTIUM TARGET HR6741A
**PDB authors**	T.A.RAMELOT, Y.YANG, H.LEE, H.JANUA, E.KOHAN, R.SHASTRY, T.B.ACTON, R.XIAO, J.K.EVERETT, J.H.PRESTEGARD, G.T.MONTELIONE, M.A.KENNEDY, NORTHEAST STRUCTURAL GENOMICS CONSORTIUM (NESG), MITOCHONDRIAL PROTEIN PARTNERSHIP (MPP)
**Last author**	KENNEDY
**Reference**	FEBS LETT. 587, 3522 (2013), doi:10.1016/J.FEBSLET.2013.09.009
**Reference authors**	T.A.RAMELOT, Y.YANG, I.D.SAHU, H.W.LEE, R.XIAO, G.A.LORIGAN, G.T.MONTELIONE, M.A.KENNEDY
**Reference title**	NMR STRUCTURE AND MD SIMULATIONS OF THE AAA PROTEASE INTERMEMBRANE SPACE DOMAIN INDICATES PERIPHERAL MEMBRANE LOCALIZATION WITHIN THE HEXAOLIGOMER
**Software listed**	AUTOSTRUCTURE, CNS, CYANA, NMRPIPE, PALES, PINE, PSVS, SPARKY, TALOS+, TOPSPIN, VNMRJ, X-PLOR_NIH
**Spectrometer**	BRUKER, VARIAN (850 MHZ, 600 MHZ)

**Protein number**	37
**PDB code**	2LA6, doi:10.2210/pdb2LA6/pdb
**BMRB code**	17508, doi:10.13018/BMR17508
**PDB Header**	RNA BINDING PROTEIN
**Protein name**	RRM DOMAIN OF RNA-BINDING PROTEIN FUS FROM HOMO SAPIENS
**Deposition date**	04.03.2011
**PDB title**	SOLUTION NMR STRUCTURE OF RRM DOMAIN OF RNA-BINDING PROTEIN FUS FROM HOMO SAPIENS, NORTHEAST STRUCTURAL GENOMICS CONSORTIUM TARGET HR6430A
**PDB authors**	G.LIU, R.XIAO, H.JANJUA, C.CICCOSANTI, H.WANG, H.LEE, T.B.ACTON, J.K.EVERETT, Y.J.HUANG, G.T.MONTELIONE, NORTHEAST STRUCTURAL GENOMICS CONSORTIUM (NESG)
**Last author**	MONTELIONE
**Reference**	
**Reference authors**	
**Reference title**	
**Software listed**	AUTOASSIGN, AUTOSTRUCTURE, CNS, CYANA, NMRPIPE, SPARKY, TALOS+, TOPSPIN, VNMRJ, XEASY
**Spectrometer**	BRUKER, VARIAN (800 MHZ, 600 MHZ)

**Protein number**	38
**PDB code**	6FIP, doi:10.2210/pdb6FIP/pdb
**BMRB code**	34235, doi:10.13018/BMR34235
**PDB Header**	TRANSPORT PROTEIN
**Protein name**	PSEUDOMONAS AERUGINOSA TONB CTD
**Deposition date**	19.01.2018
**PDB title**	SOLUTION NMR STRUCTURE OF PSEUDOMONAS AERUGINOSA TONB CTD
**PDB authors**	J.S.OEEMIG, O.H.SAMULI OLLILA, H.A.HEIKKINEN, H.IWAI
**Last author**	IWAI
**Reference**	PEERJ 6, E5412 (2018), doi:10.7717/PEERJ.5412
**Reference authors**	J.S.OEEMIG, O.H.S.OLLILA, H.IWAI
**Reference title**	NMR STRUCTURE OF THE C-TERMINAL DOMAIN OF TONB PROTEIN FROMPSEUDOMONAS AERUGINOSA
**Software listed**	AMBER, CCPNMR, CYANA, NMRPIPE
**Spectrometer**	VARIAN (800 MHZ)

**Protein number**	39
**PDB code**	2LEA, doi:10.2210/pdb2LEA/pdb
**BMRB code**	17705, doi:10.13018/BMR17705
**PDB Header**	RNA BINDING PROTEIN
**Protein name**	HUMAN SRSF2 (SC35) RRM
**Deposition date**	15.06.2011
**PDB title**	SOLUTION STRUCTURE OF HUMAN SRSF2 (SC35) RRM

**PDB authors**	G.M.DAUBNER, A.CLERY, S.JAYNE, J.STEVENIN, F.H.-T.ALLAIN
**Last author**	ALLAIN
**Reference**	EMBO J. 31, 162 (2012), doi:10.1038/EMBOJ.2011.367
**Reference authors**	G.M.DAUBNER, A.CLERY, S.JAYNE, J.STEVENIN, F.H.ALLAIN
**Reference title**	A SYN-ANTI CONFORMATIONAL DIFFERENCE ALLOWS SRSF2 TO RECOGNIZE GUANINES AND CYTOSINES EQUALLY WELL
**Software listed**	AMBER, CYANA, SPARKY
**Spectrometer**	BRUKER (900 MHZ, 700 MHZ, 600 MHZ, 500 MHZ)

**Protein number**	40
**PDB code**	2LL8, doi:10.2210/pdb2LL8/pdb
**BMRB code**	18032, doi:10.13018/BMR18032
**PDB Header**	TRANSFERASE
**Protein name**	SPECIALIZED HOLO-ACYL CARRIER PROTEIN RPA2022 FROM RHODOPSEUDOMONAS PALUSTRIS REFINED WITH NH RDCS
**Deposition date**	31.10.2011
**PDB title**	SOLUTION NMR STRUCTURE OF THE SPECIALIZED HOLO-ACYL CARRIER PROTEIN RPA2022 FROM RHODOPSEUDOMONAS PALUSTRIS REFINED WITH NH RDCS, NORTHEAST STRUCTURAL GENOMICS CONSORTIUM TARGET RPR324
**PDB authors**	T.A.RAMELOT, S.NI, P.ROSSI, Y.YANG, H.WANG, C.CICCOSANTI, M.MAGLAQUI, H.JANJUA, R.NAIR, B.ROSET, T.B.ACTON, R.XIAO, J.K.EVERETT, J.H.PRESTEGARD, G.T.MONTELIONE, M.A.KENNEDY, NORTHEAST STRUCTURAL GENOMICS CONSORTIUM (NESG)
**Last author**	KENNEDY
**Reference**	BIOCHEMISTRY 51, 7239 (2012), doi:10.1021/BI300546B
**Reference authors**	T.A.RAMELOT, P.ROSSI, F.FOROUHAR, H.W.LEE, Y.YANG, S.NI, S.UNSER, S.LEW, J.SEETHARAMAN, R.XIAO, T.B.ACTON, J.K.EVERETT, J.H.PRESTEGARD, J.F.HUNT, G.T.MONTELIONE, M.A.KENNEDY
**Reference title**	STRUCTURE OF A SPECIALIZED ACYL CARRIER PROTEIN ESSENTIAL FOR LIPID A BIOSYNTHESIS WITH VERY LONG-CHAIN FATTY ACIDS IN OPEN AND CLOSED CONFORMATIONS
**Software listed**	AUTOASSIGN, AUTOSTRUCTURE, CNS, CYANA, FMCGUI, NMRPIPE, PDBSTAT, PINE_SERVER, PSVS, SPARKY, TOPSPIN, VNMR, X-PLOR_NIH
**Spectrometer**	BRUKER, VARIAN (850 MHZ, 600 MHZ)

**Protein number**	41
**PDB code**	2KPN, doi:10.2210/pdb2KPN/pdb
**BMRB code**	16561, doi:10.13018/BMR16561
**PDB Header**	HYDROLASE
**Protein name**	A BACTERIAL IG-LIKE (BIG_3) DOMAIN FROM BACILLUS CEREUS
**Deposition date**	16.10.2009
**PDB title**	SOLUTION NMR STRUCTURE OF A BACTERIAL IG-LIKE (BIG_3) DOMAIN FROM BACILLUS CEREUS. NORTHEAST STRUCTURAL GENOMICS CONSORTIUM TARGET BCR147A
**PDB authors**	J.M.ARAMINI, D.WANG, C.T.CICCOSANTI, H.JANJUA, B.ROST, T.B.ACTON, R.XIAO, G.V.T.SWAPNA, J.K.EVERETT, G.T.MONTELIONE, NORTHEAST STRUCTURAL GENOMICS CONSORTIUM (NESG)
**Last author**	MONTELIONE
**Reference**	
**Reference authors**	
**Reference title**	
**Software listed**	AUTOSTRUCTURE, CNS, CYANA, MOLPROBITY, NMRPIPE, PDBSTAT, PINE, PSVS, SPARKY, TALOS, TOPSPIN
**Spectrometer**	BRUKER (800 MHZ, 600 MHZ)

**Protein number**	42
**PDB code**	2K0M, doi:10.2210/pdb2K0M/pdb
**BMRB code**	15652, doi:10.13018/BMR15652
**PDB Header**	STRUCTURAL GENOMICS, UNKNOWN FUNCTION
**Protein name**	UNCHARACTERIZED PROTEIN FROM RHODOSPIRILLUM RUBRUM GENE LOCUS RRU_A0810
**Deposition date**	04.02.2008
**PDB title**	SOLUTION NMR STRUCTURE OF THE UNCHARACTERIZED PROTEIN FROM RHODOSPIRILLUM RUBRUM GENE LOCUS RRU_A0810. NORTHEAST STRUCTURAL GENOMICS TARGET RRR43
**PDB authors**	P.ROSSI, H.WANG, M.JIANG, E.L.FOOTE, R.XIAO, J.LIU, G.SWAPNA, T.B.ACTON, M.C.BARAN, B.ROST, G.T.MONTELIONE, NORTHEAST STRUCTURAL GENOMICS CONSORTIUM (NESG)
**Last author**	MONTELIONE
**Reference**	
**Reference authors**	
**Reference title**	
**Software listed**	AUTOASSIGN, CNS, CYANA, MOLMOL, MOLPROBITY, NMRPIPE, PROCHECKNMR, PSVS, SPARKY, TALOS, TOPSPIN
**Spectrometer**	BRUKER (800 MHZ)

**Protein number**	43
**PDB code**	2K5V, doi:10.2210/pdb2K5V/pdb
**BMRB code**	15849, doi:10.13018/BMR15849
**PDB Header**	DNA BINDING PROTEIN
**Protein name**	SECOND OB-FOLD DOMAIN OF REPLICATION PROTEIN A FROM METHANOCOCCUS MARIPALUDIS
**Deposition date**	30.06.2008
**PDB title**	SOLUTION NMR STRUCTURE OF THE SECOND OB-FOLD DOMAIN OF REPLICATION PROTEIN A FROM METHANOCOCCUS MARIPALUDIS. NORTHEAST STRUCTURAL GENOMICS TARGET MRR110B

**PDB authors**	J.M.ARAMINI, M.MAGLAQUI, M.JIANG, C.CICCOSANTI, R.XIAO, R.NAIR, J.K.EVERETT, G.VT.SWAPNA, T.B.ACTON, B.ROST, G.T.MONTELIONE, NORTHEAST STRUCTURAL GENOMICS CONSORTIUM (NESG)
**Last author**	MONTELIONE
**Reference**	
**Reference authors**	
**Reference title**	
**Software listed**	AUTOASSIGN, AUTOSTRUCTURE, CNS, CYANA, NMRPIPE, PDBSTAT, PSVS, SPARKY, TOPSPIN
**Spectrometer**	BRUKER (800 MHZ, 600 MHZ)

**Protein number**	44
**PDB code**	2MQL, doi:10.2210/pdb2MQL/pdb
**BMRB code**	25038, doi:10.13018/BMR25038
**PDB Header**	RNA BINDING PROTEIN
**Protein name**	STRUCTURAL INVESTIGATION OF HNRNP L
**Deposition date**	24.06.2014
**PDB title**	STRUCTURAL INVESTIGATION OF HNRNP L
**PDB authors**	M.BLATTER, F.ALLAIN
**Last author**	ALLAIN
**Reference**	J.MOL.BIOL. 427, 3001 (2015), doi:10.1016/J.JMB.2015.05.020
**Reference authors**	M.BLATTER, S.DUNIN-HORKAWICZ, I.GRISHINA, C.MARIS, S.THORE, T.MAIER, A.BINDEREIF, J.M.BUJNICKI, F.H.ALLAIN
**Reference title**	THE SIGNATURE OF THE FIVE-STRANDED VRRM FOLD DEFINED BY FUNCTIONAL, STRUCTURAL AND COMPUTATIONAL ANALYSIS OF THE HNRNP L PROTEIN
**Software listed**	AMBER, CYANA, SPARKY, TOPSPIN
**Spectrometer**	BRUKER (900 MHZ, 700 MHZ)

**Protein number**	45
**PDB code**	2K75, doi:10.2210/pdb2K75/pdb
**BMRB code**	15902, doi:10.13018/BMR15902
**PDB Header**	DNA BINDING PROTEIN
**Protein name**	OB DOMAIN OF TA0387 FROM THERMOPLASMA ACIDOPHILUM
**Deposition date**	01.08.2008
**PDB title**	SOLUTION NMR STRUCTURE OF THE OB DOMAIN OF TA0387 FROM THERMOPLASMA ACIDOPHILUM. NORTHEAST STRUCTURAL GENOMICS CONSORTIUM TARGET TAR80B
**PDB authors**	T.A.RAMELOT, K.DING, D.LEE, M.JIANG, C.CICCOSANTI, R.XIAO, R.NAIR, J.K.EVERETT, G.SWAPNA, T.B.ACTON, B.ROST, G.T.MONTELIONE, M.A.KENNEDY, NORTHEAST STRUCTURAL GENOMICS CONSORTIUM (NESG)
**Last author**	KENNEDY
**Reference**	
**Reference authors**	
**Reference title**	
**Software listed**	AUTOASSIGN, AUTOSTRUCTURE, NMRPIPE, PSVS, SPARKY, TOPSPIN, VNMR, X-PLOR
**Spectrometer**	BRUKER, VARIAN (850 MHZ, 600 MHZ)

**Protein number**	46
**PDB code**	2LTM, doi:10.2210/pdb2LTM/pdb
**BMRB code**	18489, doi:10.13018/BMR18489
**PDB Header**	ELECTRON TRANSPORT
**Protein name**	NFU1 IRON-SULFUR CLUSTER SCAFFOLD HOMOLOG FROM HOMO SAPIENS
**Deposition date**	29.05.2012
**PDB title**	SOLUTION NMR STRUCTURE OF NFU1 IRON-SULFUR CLUSTER SCAFFOLD HOMOLOG FROM HOMO SAPIENS, NORTHEAST STRUCTURAL GENOMICS CONSORTIUM (NESG) TARGET HR2876B
**PDB authors**	G.LIU, R.XIAO, H.JANJUA, K.HAMILTON, R.SHASTRY, E.KOHAN, T.B.ACTON, J.K.EVERETT, H.LEE, Y.J.HUANG, G.T.MONTELIONE, NORTHEAST STRUCTURAL GENOMICS CONSORTIUM (NESG), MITOCHONDRIAL PROTEIN PARTNERSHIP (MPP)
**Last author**	MONTELIONE
**Reference**	
**Reference authors**	
**Reference title**	
**Software listed**	AUTOASSIGN, AUTOSTRUCTURE, CNS, CYANA, NMRPIPE, PALES, PSVS, REDCAT, SPARKY, TALOS+, TOPSPIN, VNMRJ, XEASY
**Spectrometer**	BRUKER, VARIAN (800 MHZ, 600 MHZ)

**Protein number**	47
**PDB code**	2KOB, doi:10.2210/pdb2KOB/pdb
**BMRB code**	16498, doi:10.13018/BMR16498
**PDB Header**	STRUCTURAL GENOMICS, UNKNOWN FUNCTION
**Protein name**	CLOLEP_01837 (FRAGMENT 61-160) FROM CLOSTRIDIUM LEPTUM
**Deposition date**	15.09.2009
**PDB title**	SOLUTION NMR STRUCTURE OF CLOLEP_01837 (FRAGMENT 61-160) FROM CLOSTRIDIUM LEPTUM. NORTHEAST STRUCTURAL GENOMICS CONSORTIUM TARGET QLR8A
**PDB authors**	T.A.RAMELOT, D.LEE, C.CICCOSANTI, M.JIANG, R.NAIR, B.ROST, T.B.ACTON, R.XIAO, J.K.EVERETT, G.T.MONTELIONE, M.A.KENNEDY, NORTHEAST STRUCTURAL GENOMICS CONSORTIUM (NESG)
**Last author**	KENNEDY
**Reference**	
**Reference authors**	
**Reference title**	

**Software listed**	AUTOASSIGN, AUTOSTRUCTURE, CNS, NMRPIPE, PDBSTAT, PSVS, SPARKY, TOPSPIN, VNMR, X-PLOR
**Spectrometer**	BRUKER, VARIAN (850 MHZ, 600 MHZ)

**Protein number**	48
**PDB code**	2KHD, doi:10.2210/pdb2KHD/pdb
**BMRB code**	16238, doi:10.13018/BMR16238
**PDB Header**	STRUCTURAL GENOMICS, UNKNOWN FUNCTION
**Protein name**	VC_A0919 FROM VIBRIO CHOLERAE
**Deposition date**	02.04.2009
**PDB title**	SOLUTION NMR STRUCTURE OF VC_A0919 FROM VIBRIO CHOLERAE. NORTHEAST STRUCTURAL GENOMICS CONSORTIUM TARGET VCR52
**PDB authors**	T.A.RAMELOT, J.R.CORT, H.WANG, C.CICCOSANTI, M.JIANG, J.LIU, B.ROST, G.V.T.SWAPNA, T.B.ACTON, R.XIAO, J.K.EVERETT, G.T.MONTELIONE, M.A.KENNEDY, NORTHEAST STRUCTURAL GENOMICS CONSORTIUM (NESG)
**Last author**	KENNEDY
**Reference**	
**Reference authors**	
**Reference title**	
**Software listed**	AUTOASSIGN, AUTOSTRUCTURE, NMRPIPE, PDBSTAT, PSVS, SPARKY, TOPSPIN, VNMR, X-PLOR
**Spectrometer**	BRUKER, VARIAN (850 MHZ, 750 MHZ, 600 MHZ)

**Protein number**	49
**PDB code**	2RN7, doi:10.2210/pdb2RN7/pdb
**BMRB code**	11017, doi:10.13018/BMR11017
**PDB Header**	UNKNOWN FUNCTION
**Protein name**	TNPE PROTEIN FROM SHIGELLA FLEXNERI
**Deposition date**	08.12.2007
**PDB title**	NMR SOLUTION STRUCTURE OF TNPE PROTEIN FROM SHIGELLA FLEXNERI. NORTHEAST STRUCTURAL GENOMICS TARGET SFR125
**PDB authors**	T.A.RAMELOT, J.R.CORT, A.SEMESI, M.GARCIA, A.A.YEE, C.H.ARROWSMITH, M.A.KENNEDY, NORTHEAST STRUCTURAL GENOMICS CONSORTIUM (NESG)
**Last author**	KENNEDY
**Reference**	
**Reference authors**	
**Reference title**	
**Software listed**	AUTOSTRUCTURE, CNS, NMRPIPE, SPARKY, VNMR, X-PLOR
**Spectrometer**	VARIAN (750 MHZ, 600 MHZ)

**Protein number**	50
**PDB code**	2LXU, doi:10.2210/pdb2LXU/pdb
**BMRB code**	18698, doi:10.13018/BMR18698
**PDB Header**	RNA BINDING PROTEIN
**Protein name**	EUKARYOTIC RNA RECOGNITION MOTIF, RRM1, FROM HETEROGENEOUS NUCLEAR RIBONUCLEOPROTEIN H FROM HOMO SAPIENS
**Deposition date**	31.08.2012
**PDB title**	SOLUTION NMR STRUCTURE OF THE EUKARYOTIC RNA RECOGNITION MOTIF, RRM1, FROM THE HETEROGENEOUS NUCLEAR RIBONUCLEOPROTEIN H FROM HOMO SAPIENS, NORTHEAST STRUCTURAL GENOMICS CONSORTIUM (NESG) TARGET HR8614A
**PDB authors**	T.A.RAMELOT, Y.YANG, K.PEDERSON, R.SHASTRY, E.KOHAN, H.JANJUA, R.XIAO, T.B.ACTON, J.K.EVERETT, J.H.PRESTEGARD, G.T.MONTELIONE, M.A.KENNEDY, NORTHEAST STRUCTURAL GENOMICS CONSORTIUM (NESG)
**Last author**	KENNEDY
**Reference**	
**Reference authors**	
**Reference title**	
**Software listed**	AUTOSTRUCTURE, CNS, CYANA, FMCGUI, NMRPIPE, PALES, PINE, PSVS, SPARKY, TALOS+, TOPSPIN, VNMRJ
**Spectrometer**	BRUKER, VARIAN (800 MHZ, 600 MHZ)

**Protein number**	51
**PDB code**	2KIF, doi:10.2210/pdb2KIF/pdb
**BMRB code**	16272, doi:10.13018/BMR16272
**PDB Header**	TRANSFERASE
**Protein name**	AN O6-METHYLGUANINE DNA METHYLTRANSFERASE FAMILY PROTEIN FROM VIBRIO PARAHAEMOLYTICUS
**Deposition date**	03.05.2009
**PDB title**	SOLUTION NMR STRUCTURE OF AN O6-METHYLGUANINE DNA METHYLTRANSFERASE FAMILY PROTEIN FROM VIBRIO PARAHAEMOLYTICUS. NORTHEAST STRUCTURAL GENOMICS CONSORTIUM TARGET VPR247
**PDB authors**	J.M.ARAMINI, R.L.BELOTE, C.T.CICCOSANTI, M.JIANG, B.ROST, R.NAIR, G.V.T.SWAPNA, T.B.ACTON, R.XIAO, J.K.EVERETT, G.T.MONTELIONE, NORTHEAST STRUCTURAL GENOMICS CONSORTIUM (NESG)
**Last author**	MONTELIONE
**Reference**	J.BIOL.CHEM. 285, 13736 (2010), doi:10.1074/JBC.M109.093591
**Reference authors**	J.M.ARAMINI, J.L.TUBBS, S.KANUGULA, P.ROSSI, A.ERTEKIN, M.MAGLAQUI, K.HAMILTON, C.T.CICCOSANTI, M.JIANG, R.XIAO, T.T.SOONG, B.ROST, T.B.ACTON, J.K.EVERETT, A.E.PEGG, J.A.TAINER, G.T.MONTELIONE

**Reference title**	STRUCTURAL BASIS OF O6-ALKYLGUANINE RECOGNITION BY A BACTERIAL ALKYLTRANSFERASE-LIKE DNA REPAIR PROTEIN
**Software listed**	AUTOASSIGN, AUTOSTRUCTURE, CNS, CYANA, MOLPROBITY, NMRPIPE, PDBSTAT, PINE, PSVS, SPARKY, TOPSPIN
**Spectrometer**	BRUKER (800 MHZ, 600 MHZ)

**Protein number**	52
**PDB code**	2KBN, doi:10.2210/pdb2KBN/pdb
**BMRB code**	16051, doi:10.13018/BMR16051
**PDB Header**	STRUCTURAL GENOMICS, UNKNOWN FUNCTION
**Protein name**	OB DOMAIN (RESIDUES 67-166) OF MM0293 FROM METHANOSARCINA MAZEI
**Deposition date**	03.12.2008
**PDB title**	SOLUTION NMR STRUCTURE OF THE OB DOMAIN (RESIDUES 67-166) OF MM0293 FROM METHANOSARCINA MAZEI. NORTHEAST STRUCTURAL GENOMICS CONSORTIUM TARGET MAR214A
**PDB authors**	T.A.RAMELOT, K.DING, M.MAGLIQUI, M.JIANG, C.CICCOSANTI, R.XIAO, J.LUI, J.K.EVERETT, G.SWAPNA, T.B.ACTON, B.ROST, G.T.MONTELIONE, M.A.KENNEDY, NORTHEAST STRUCTURAL GENOMICS CONSORTIUM (NESG)
**Last author**	KENNEDY
**Reference**	
**Reference authors**	
**Reference title**	
**Software listed**	AUTOASSIGN, AUTOSTRUCTURE, CNS, CYANA, PSVS, SPARKY, TOPSPIN, X-PLOR
**Spectrometer**	BRUKER, VARIAN (850 MHZ, 600 MHZ)

**Protein number**	53
**PDB code**	2MK2, doi:10.2210/pdb2MK2/pdb
**BMRB code**	19749, doi:10.13018/BMR19749
**PDB Header**	HYDROLASE
**Protein name**	N-TERMINAL DOMAIN (SH2 DOMAIN) OF HUMAN INOSITOL POLYPHOSPHATE PHOSPHATASE-LIKE PROTEIN 1 (INPPL1) (FRAGMENT 20-117)
**Deposition date**	23.01.2014
**PDB title**	SOLUTION NMR STRUCTURE OF N-TERMINAL DOMAIN (SH2 DOMAIN) OF HUMAN INOSITOL POLYPHOSPHATE PHOSPHATASE-LIKE PROTEIN 1 (INPPL1) (FRAGMENT 20-117), NORTHEAST STRUCTURAL GENOMICS CONSORTIUM TARGET HR9134A
**PDB authors**	Y.YANG, T.A.RAMELOT, H.JANJUA, R.XIAO, J.K.EVERETT, G.T.MONTELIONE, M.A.KENNEDY, NORTHEAST STRUCTURAL GENOMICS CONSORTIUM (NESG)
**Last author**	KENNEDY
**Reference**	
**Reference authors**	
**Reference title**	
**Software listed**	AUTOASSIGN, AUTOSTRUCTURE, CNS, CYANA, NMRPIPE, PALES, PINE, PSVS, REDCAT, SPARKY, TALOS+, TOPSPIN, VNMRJ, XEASY
**Spectrometer**	BRUKER, VARIAN (850 MHZ, 600 MHZ)

**Protein number**	54
**PDB code**	2K50, doi:10.2210/pdb2K50/pdb
**BMRB code**	15819, doi:10.13018/BMR15819
**PDB Header**	STRUCTURAL GENOMICS, UNKNOWN FUNCTION
**Protein name**	REPLICATION FACTOR A RELATED PROTEIN FROM METHANOBACTERIUM THERMOAUTOTROPHICUM
**Deposition date**	23.06.2008
**PDB title**	SOLUTION NMR STRUCTURE OF THE REPLICATION FACTOR A RELATED PROTEIN FROM METHANOBACTERIUM THERMOAUTOTROPHICUM. NORTHEAST STRUCTURAL GENOMICS TARGET TR91A
**PDB authors**	P.ROSSI, R.XIAO, M.MAGLAQUI, E.L.FOOTE, C.CICCOSANTI, G.SWAPNA, T.B.ACTON, B.ROST, J.K.EVERETT, M.JIANG, R.NAIR, G.T.MONTELIONE, NORTHEAST STRUCTURAL GENOMICS CONSORTIUM (NESG)
**Last author**	MONTELIONE
**Reference**	
**Reference authors**	
**Reference title**	
**Software listed**	AUTOASSIGN, CNS, CYANA, MOLMOL, MOLPROBITY, NMRPIPE, PROCHECK, PSVS, RPF(AUTOSTRUCTURE), SPARKY, TALOS, TOPSPIN
**Spectrometer**	BRUKER (800 MHZ, 600 MHZ)

**Protein number**	55
**PDB code**	2KL5, doi:10.2210/pdb2KL5/pdb
**BMRB code**	16384, doi:10.13018/BMR16384
**PDB Header**	STRUCTURAL GENOMICS, UNKNOWN FUNCTION
**Protein name**	PROTEIN YUTD FROM B.SUBTILIS
**Deposition date**	30.06.2009
**PDB title**	SOLUTION NMR STRUCTURE OF PROTEIN YUTD FROM B.SUBTILIS, NORTHEAST STRUCTURAL GENOMICS CONSORTIUM TARGET SR232
**PDB authors**	G.LIU, K.HAMILTON, R.XIAO, C.CICCOSANTI, C.J.HO, J.EVERETT, R.NAIR, T.ACTON, B.ROST, G.T.MONTELIONE, NORTHEAST STRUCTURAL GENOMICS CONSORTIUM (NESG)
**Last author**	MONTELIONE
**Reference**	
**Reference authors**	
**Reference title**	
**Software listed**	AUTOASSIGN, AUTOSTRUCTURE, CNS, CYANA, NMRPIPE, TOPSPIN, VNMRJ, XEASY

**Spectrometer**	BRUKER, VARIAN (800 MHZ, 600 MHZ)

**Protein number**	56
**PDB code**	2LTA, doi:10.2210/pdb2LTA/pdb
**BMRB code**	18465, doi:10.13018/BMR18465
**PDB Header**	DE NOVO PROTEIN
**Protein name**	DE NOVO DESIGNED PROTEIN, ROSSMANN 3X1 FOLD
**Deposition date**	15.05.2012
**PDB title**	SOLUTION NMR STRUCTURE OF DE NOVO DESIGNED PROTEIN, ROSSMANN 3X1 FOLD, NORTHEAST STRUCTURAL GENOMICS CONSORTIUM TARGET OR157
**PDB authors**	G.LIU, R.KOGA, N.KOGA, R.XIAO, K.PEDERSON, K.HAMILTON, E.KOHAN, T.B.ACTON, G.KORNHABER, J.K.EVERETT, D.BAKER, G.T.MONTELIONE, NORTHEAST STRUCTURAL GENOMICS CONSORTIUM (NESG)
**Last author**	MONTELIONE
**Reference**	NATURE 491, 222 (2012), doi:10.1038/NATURE11600
**Reference authors**	N.KOGA, R.TATSUMI-KOGA, G.LIU, R.XIAO, T.B.ACTON, G.T.MONTELIONE, D.BAKER
**Reference title**	PRINCIPLES FOR DESIGNING IDEAL PROTEIN STRUCTURES
**Software listed**	AUTOASSIGN, AUTOSTRUCTURE, CNS, CYANA, NMRPIPE, PSVS, REDCAT, SPARKY, TALOS+, TOPSPIN, VNMRJ, XEASY
**Spectrometer**	BRUKER, VARIAN (800 MHZ, 600 MHZ)

**Protein number**	57
**PDB code**	2KIW, doi:10.2210/pdb2KIW/pdb
**BMRB code**	16298, doi:10.13018/BMR16298
**PDB Header**	DNA BINDING PROTEIN
**Protein name**	DOMAIN N-TERMINAL TO INTEGRASE DOMAIN OF SH1003 FROM STAPHYLOCOCCUS HAEMOLYTICUS
**Deposition date**	12.05.2009
**PDB title**	SOLUTION NMR STRUCTURE OF THE DOMAIN N-TERMINAL TO THE INTEGRASE DOMAIN OF SH1003 FROM STAPHYLOCOCCUS HAEMOLYTICUS. NORTHEAST STRUCTURAL GENOMICS CONSORTIUM TARGET SHR105F (64-166)
**PDB authors**	Y.YANG, T.A.RAMELOT, R.L.BELOTE, E.L.FOOTE, H.JANJUA, R.NAIR, B.ROST, G.SWAPNA, T.B.ACTON, R.XIAO, J.K.EVERETT, G.T.MONTELIONE, M.A.KENNEDY, NORTHEAST STRUCTURAL GENOMICS CONSORTIUM (NESG)
**Last author**	KENNEDY
**Reference**	
**Reference authors**	
**Reference title**	
**Software listed**	AUTOASSIGN, AUTOSTRUCTURE, CNS, NMRPIPE, PDBSTAT, PSVS, SPARKY, TOPSPIN, VNMR, X-PLOR
**Spectrometer**	BRUKER, VARIAN (850 MHZ, 600 MHZ)

**Protein number**	58
**PDB code**	2LVB, doi:10.2210/pdb2LVB/pdb
**BMRB code**	18561, doi:10.13018/BMR18561
**PDB Header**	DE NOVO PROTEIN
**Protein name**	SOLUTION NMR STRUCTURE DE NOVO DESIGNED PFK FOLD PROTEIN
**Deposition date**	30.06.2012
**PDB title**	SOLUTION NMR STRUCTURE DE NOVO DESIGNED PFK FOLD PROTEIN, NORTHEAST STRUCTURAL GENOMICS CONSORTIUM (NESG) TARGET OR250
**PDB authors**	G.LIU, N.KOGA, R.KOGA, R.XIAO, K.HAMILTON, E.KOHAN, T.B.ACTON, G.KORNHABER, J.K.EVERETT, D.BAKER, G.T.MONTELIONE, NORTHEAST STRUCTURAL GENOMICS CONSORTIUM (NESG)
**Last author**	MONTELIONE
**Reference**	NATURE 491, 222 (2012), doi:10.1038/NATURE11600
**Reference authors**	N.KOGA, R.TATSUMI-KOGA, G.LIU, R.XIAO, T.B.ACTON, G.T.MONTELIONE, D.BAKER
**Reference title**	PRINCIPLES FOR DESIGNING IDEAL PROTEIN STRUCTURES
**Software listed**	AUTOASSIGN, AUTOSTRUCTURE, CNS, CYANA, NMRPIPE, PSVS, SPARKY, TALOS+, TOPSPIN, VNMRJ, XEASY
**Spectrometer**	BRUKER, VARIAN (800 MHZ, 600 MHZ)

**Protein number**	59
**PDB code**	2LND, doi:10.2210/pdb2LND/pdb
**BMRB code**	18161, doi:10.13018/BMR18161
**PDB Header**	DE NOVO PROTEIN
**Protein name**	DE NOVO DESIGNED PROTEIN, PFK FOLD
**Deposition date**	23.12.2011
**PDB title**	SOLUTION NMR STRUCTURE OF DE NOVO DESIGNED PROTEIN, PFK FOLD, NORTHEAST STRUCTURAL GENOMICS CONSORTIUM TARGET OR134
**PDB authors**	G.LIU, N.KOGA, R.KOGA, R.XIAO, H.LEE, H.JANJUA, E.KOHAN, T.B.ACTON, J.K.EVERETT, D.BAKER, G.T.MONTELIONE, NORTHEAST STRUCTURAL GENOMICS CONSORTIUM (NESG)
**Last author**	MONTELIONE
**Reference**	
**Reference authors**	
**Reference title**	
**Software listed**	AUTOASSIGN, AUTOSTRUCTURE, CNS, CYANA, NMRPIPE, PSVS, SPARKY, TALOS+, TOPSPIN, VNMRJ, XEASY
**Spectrometer**	BRUKER, VARIAN (800 MHZ, 600 MHZ)

**Protein number**	60
**PDB code**	1WQU, doi:10.2210/pdb1WQU/pdb
**BMRB code**	6331, doi:10.13018/BMR6331
**PDB Header**	TRANSFERASE
**Protein name**	HUMAN FES SH2 DOMAIN
**Deposition date**	02.10.2004
**PDB title**	SOLUTION STRUCTURE OF THE HUMAN FES SH2 DOMAIN
**PDB authors**	A.SCOTT, D.PANTOJA-UCEDA, S.KOSHIBA, M.INOUE, T.KIGAWA, T.TERADA, M.SHIROUZU, A.TANAKA, S.SUGANO, S.YOKOYAMA, P.GUNTERT, RIKEN STRUCTURAL GENOMICS/PROTEOMICS INITIATIVE (RSGI)
**Last author**	GUNTERT
**Reference**	J.BIOMOL.NMR 31, 357 (2005), doi:10.1007/S10858-005-0946-6
**Reference authors**	A.SCOTT, D.PANTOJA-UCEDA, S.KOSHIBA, M.INOUE, T.KIGAWA, T.TERADA, M.SHIROUZU, A.TANAKA, S.SUGANO, S.YOKOYAMA, P.GUNTERT
**Reference title**	SOLUTION STRUCTURE OF THE SRC HOMOLOGY 2 DOMAIN FROM THE HUMAN FELINE SARCOMA ONCOGENE FES
**Software listed**	CYANA, NMRPIPE, NMRVIEW, OPALP
**Spectrometer**	BRUKER (800 MHZ, 600 MHZ)

**Protein number**	61
**PDB code**	2KL6, doi:10.2210/pdb2KL6/pdb
**BMRB code**	16385, doi:10.13018/BMR16385
**PDB Header**	STRUCTURAL GENOMICS, UNKNOWN FUNCTION
**Protein name**	CARDB DOMAIN OF PF1109 FROM PYROCOCCUS FURIOSUS
**Deposition date**	30.06.2009
**PDB title**	SOLUTION NMR STRUCTURE OF THE CARDB DOMAIN OF PF1109 FROM PYROCOCCUS FURIOSUS. NORTHEAST STRUCTURAL GENOMICS CONSORTIUM TARGET PFR193A
**PDB authors**	J.M.ARAMINI, D.LEE, C.CICCOSANTI, K.HAMILTON, R.NAIR, B.ROST, T.B.ACTON, R.XIAO, G.V.T.SWAPNA, J.K.EVERETT, G.T.MONTELIONE, NORTHEAST STRUCTURAL GENOMICS CONSORTIUM (NESG)
**Last author**	MONTELIONE
**Reference**	
**Reference authors**	
**Reference title**	
**Software listed**	AUTOSTRUCTURE, CNS, CYANA, MOLPROBITY, NMRPIPE, PDBSTAT, PINE, PSVS, SPARKY, TALOS, TOPSPIN
**Spectrometer**	BRUKER (800 MHZ, 600 MHZ)

**Protein number**	62
**PDB code**	6GT7, doi:10.2210/pdb6GT7/pdb
**BMRB code**	34287, doi:10.13018/BMR34287
**PDB Header**	TRANSFERASE
**Protein name**	FREE HELIX BUNDLE DOMAIN FROM FUNCTIONAL PRN1 PRIMASE
**Deposition date**	15.06.2018
**PDB title**	NMR STRUCTURE OF THE FREE HELIX BUNDLE DOMAIN FROM THE FUNCTIONAL PRN1 PRIMASE
**PDB authors**	J.BOUDET, G.LIPPS, F.ALLAIN
**Last author**	ALLAIN
**Reference**	CELL 176, 154 (2019), doi:10.1016/J.CELL.2018.11.031
**Reference authors**	J.BOUDET, J.C.DEVILLIER, T.WIEGAND, L.SALMON, B.H.MEIER, G.LIPPS, F.H.ALLAIN
**Reference title**	A SMALL HELICAL BUNDLE PREPARES PRIMER SYNTHESIS BY BINDING TWO NUCLEOTIDES THAT ENHANCE SEQUENCE-SPECIFIC RECOGNITION OF THE DNA TEMPLATE
**Software listed**	AMBER, CANDID, CYANA, SPARKY
**Spectrometer**	BRUKER (900 MHZ, 700 MHZ, 600 MHZ)

**Protein number**	63
**PDB code**	2JN8, doi:10.2210/pdb2JN8/pdb
**BMRB code**	15089, doi:10.13018/BMR15089
**PDB Header**	STRUCTURAL GENOMICS, UNKNOWN FUNCTION
**Protein name**	Q8ZRJ2 FROM SALMONELLA TYPHIMURIUM
**Deposition date**	29.12.2006
**PDB title**	SOLUTION NMR STRUCTURE OF Q8ZRJ2 FROM SALMONELLA TYPHIMURIUM. NORTHEAST STRUCTURAL GENOMICS TARGET STR65
**PDB authors**	J.M.ARAMINI, J.R.CORT, C.K.HO, K.CUNNINGHAM, L.-C.MA, R.XIAO, J.LIU, M.C.BARAN, G.V.T.SWAPNA, T.B.ACTON, B.ROST, G.T.MONTELIONE, NORTHEAST STRUCTURAL GENOMICS CONSORTIUM (NESG)
**Last author**	MONTELIONE
**Reference**	
**Reference authors**	
**Reference title**	
**Software listed**	AUTOASSIGN, AUTOSTRUCTURE, BRUKER, NMRPIPE, PDBSTAT, PSVS, SPARKY, VNMR, X-PLOR
**Spectrometer**	BRUKER, VARIAN (750 MHZ, 600 MHZ, 500 MHZ)

**Protein number**	64
**PDB code**	2K5D, doi:10.2210/pdb2K5D/pdb
**BMRB code**	15829, doi:10.13018/BMR15829
**PDB Header**	STRUCTURAL GENOMICS, UNKNOWN FUNCTION
**Protein name**	SAG0934 FROM STREPTOCOCCUS AGALACTIAE
**Deposition date**	26.06.2008

**PDB title**	SOLUTION NMR STRUCTURE OF SAG0934 FROM STREPTOCOCCUS AGALACTIAE. NORTHEAST STRUCTURAL GENOMICS TARGET SAR32[1-108]
**PDB authors**	J.M.ARAMINI, P.ROSSI, L.ZHAO, E.L.FOOTE, M.JIANG, R.XIAO, S.SHARMA, G.VT.SWAPNA, R.NAIR, J.K.EVERETT, T.B.ACTON, B.ROST, G.T.MONTELIONE, NORTHEAST STRUCTURAL GENOMICS CONSORTIUM (NESG)
**Last author**	MONTELIONE
**Reference**	
**Reference authors**	
**Reference title**	
**Software listed**	AUTOASSIGN, AUTOSTRUCTURE, CNS, CYANA, NMRPIPE, PDBSTAT, PSVS, SPARKY, TOPSPIN
**Spectrometer**	BRUKER (800 MHZ, 600 MHZ)

**Protein number**	65
**PDB code**	2KD1, doi:10.2210/pdb2KD1/pdb
**BMRB code**	16102, doi:10.13018/BMR16102
**PDB Header**	STRUCTURAL GENOMICS, UNKNOWN FUNCTION
**Protein name**	INTEGRASE-LIKE DOMAIN FROM BACILLUS CEREUS ORDERED LOCUS BC_1272
**Deposition date**	31.12.2008
**PDB title**	SOLUTION NMR STRUCTURE OF THE INTEGRASE-LIKE DOMAIN FROM BACILLUS CEREUS ORDERED LOCUS BC_1272. NORTHEAST STRUCTURAL GENOMICS CONSORTIUM TARGET BCR268F
**PDB authors**	P.ROSSI, H.LEE, M.MAGLAQUI, E.L.FOOTE, W.A.BUCHWALD, M.JIANG, G.V.T.SWAPNA, R.NAIR, R.XIAO, T.B.ACTON, B.ROST, J.H.PRESTEGARD, G.T.MONTELIONE, NORTHEAST STRUCTURAL GENOMICS CONSORTIUM (NESG)
**Last author**	MONTELIONE
**Reference**	
**Reference authors**	
**Reference title**	
**Software listed**	CNS, CYANA, MOLMOL, MOLPROBITY, NMRPIPE, PDBSTAT, PINE, PROCHECK, PSVS, SPARKY, TALOS, TOPSPIN
**Spectrometer**	BRUKER, VARIAN (800 MHZ, 600 MHZ)

**Protein number**	66
**PDB code**	2LTL, doi:10.2210/pdb2LTL/pdb
**BMRB code**	18487, doi:10.13018/BMR18487
**PDB Header**	ELECTRON TRANSPORT
**Protein name**	NIFU-LIKE PROTEIN FROM SACCHAROMYCES CEREVISIAE
**Deposition date**	29.05.2012
**PDB title**	SOLUTION NMR STRUCTURE OF NIFU-LIKE PROTEIN FROM SACCHAROMYCES CEREVISIAE, NORTHEAST STRUCTURAL GENOMICS CONSORTIUM (NESG) TARGET YR313A
**PDB authors**	G.LIU, R.XIAO, K.HAMILTON, H.JANJUA, R.SHASTRY, E.KOHAN, T.B.ACTON, J.K.EVERETT, H.LEE, Y.J.HUANG, G.T.MONTELIONE, NORTHEAST STRUCTURAL GENOMICS CONSORTIUM (NESG), MITOCHONDRIAL PROTEIN PARTNERSHIP (MPP)
**Last author**	MONTELIONE
**Reference**	
**Reference authors**	
**Reference title**	
**Software listed**	AUTOASSIGN, AUTOSTRUCTURE, CNS, CYANA, NMRPIPE, PALES, PINE, PSVS, REDCAT, SPARKY, TALOS+, TOPSPIN, VNMRJ, XEASY
**Spectrometer**	BRUKER, VARIAN (800 MHZ, 600 MHZ)

**Protein number**	67
**PDB code**	2KVO, doi:10.2210/pdb2KVO/pdb
**BMRB code**	16782, doi:10.13018/BMR16782
**PDB Header**	PHOTOSYNTHESIS
**Protein name**	PHOTOSYSTEM II REACTION CENTER PSB28 PROTEIN FROM SYNECHOCYSTIS SP.(STRAIN PCC 6803)
**Deposition date**	22.03.2010
**PDB title**	SOLUTION NMR STRUCTURE OF PHOTOSYSTEM II REACTION CENTER PSB28 PROTEIN FROM SYNECHOCYSTIS SP.(STRAIN PCC 6803), NORTHEAST STRUCTURAL GENOMICS CONSORTIUM TARGET SGR171
**PDB authors**	Y.YANG, T.A.RAMELOT, J.R.CORT, D.WANG, C.CICCOSANTI, K.HAMILTON, R.NAIR, B.ROST, T.B.ACTON, R.XIAO, J.K.EVERETT, G.T.MONTELIONE, M.A.KENNEDY, NORTHEAST STRUCTURAL GENOMICS CONSORTIUM (NESG)
**Last author**	KENNEDY
**Reference**	PROTEINS 79, 340 (2011), doi:10.1002/PROT.22876
**Reference authors**	Y.YANG, T.A.RAMELOT, J.R.CORT, D.WANG, C.CICCOSANTI, K.HAMILTON, R.NAIR, B.ROST, T.B.ACTON, R.XIAO, J.K.EVERETT, G.T.MONTELIONE, M.A.KENNEDY
**Reference title**	SOLUTION NMR STRUCTURE OF PHOTOSYSTEM II REACTION CENTER PROTEIN PSB28 FROM SYNECHOCYSTIS SP. STRAIN PCC 6803
**Software listed**	AUTOASSIGN, AUTOSTRUCTURE, CNS, NMRPIPE, PDBSTAT, PINE_SERVER, PSVS, SPARKY, TOPSPIN, VNMR, X-PLOR_NIH
**Spectrometer**	BRUKER, VARIAN (850 MHZ, 600 MHZ)

**Protein number**	68
**PDB code**	1T0Y, doi:10.2210/pdb1T0Y/pdb
**BMRB code**	6176, doi:10.13018/BMR6176
**PDB Header**	CHAPERONE
**Protein name**	A UBIQUITIN-LIKE DOMAIN FROM TUBULIN-BINDING COFACTOR B

**Deposition date**	13.04.2004
**PDB title**	SOLUTION STRUCTURE OF A UBIQUITIN-LIKE DOMAIN FROM TUBULIN-BINDING COFACTOR B
**PDB authors**	B.L.LYTLE, F.C.PETERSON, S.H.QUI, M.LUO, B.F.VOLKMAN, J.L.MARKLEY, CENTER FOR EUKARYOTIC STRUCTURAL GENOMICS (CESG)
**Last author**	MARKLEY
**Reference**	J.BIOL.CHEM. 279, 46787 (2004), doi:10.1074/JBC.M409422200
**Reference authors**	B.L.LYTLE, F.C.PETERSON, S.H.QIU, M.LUO, Q.ZHAO, J.L.MARKLEY, B.F.VOLKMAN
**Reference title**	SOLUTION STRUCTURE OF A UBIQUITIN-LIKE DOMAIN FROM TUBULIN-BINDING COFACTOR B
**Software listed**	CYANA, NMRPIPE, SPSCAN, X-PLOR_NIH, XEASY, XWINNMR
**Spectrometer**	BRUKER (600 MHZ)

**Protein number**	69
**PDB code**	2KCD, doi:10.2210/pdb2KCD/pdb
**BMRB code**	16072, doi:10.13018/BMR16072
**PDB Header**	STRUCTURAL GENOMICS, UNKNOWN FUNCTION
**Protein name**	SSP0047 FROM STAPHYLOCOCCUS SAPROPHYTICUS
**Deposition date**	19.12.2008
**PDB title**	SOLUTION NMR STRUCTURE OF SSP0047 FROM STAPHYLOCOCCUS SAPROPHYTICUS. NORTHEAST STRUCTURAL GENOMICS CONSORTIUM TARGET SYR6
**PDB authors**	T.A.RAMELOT, K.DING, C.X.CHEN, M.JIANG, C.CICCOSANTI, R.XIAO, J.LIU, M.C.BARAN, G.SWAPNA, T.B.ACTON, B.ROST, G.T.MONTELIONE, M.A.KENNEDY, NORTHEAST STRUCTURAL GENOMICS CONSORTIUM (NESG)
**Last author**	KENNEDY
**Reference**	
**Reference authors**	
**Reference title**	
**Software listed**	AUTOASSIGN, AUTOSTRUCTURE, NMRPIPE, PDBSTAT, PSVS, SPARKY, TOPSPIN, VNMR, X-PLOR
**Spectrometer**	BRUKER, VARIAN (850 MHZ, 750 MHZ, 600 MHZ)

**Protein number**	70
**PDB code**	2KRT, doi:10.2210/pdb2KRT/pdb
**BMRB code**	16648, doi:10.13018/BMR16648
**PDB Header**	LIPID BINDING PROTEIN
**Protein name**	A CONSERVED HYPOTHETICAL MEMBRANE LIPOPROTEIN OBTAINED FROM UREAPLASMA PARVUM
**Deposition date**	22.12.2009
**PDB title**	SOLUTION NMR STRUCTURE OF A CONSERVED HYPOTHETICAL MEMBRANE LIPOPROTEIN OBTAINED FROM UREAPLASMA PARVUM: NORTHEAST STRUCTURAL GENOMICS CONSORTIUM TARGET UUR17A (139-239)
**PDB authors**	R.MANI, G.SWAPNA, H.JANJUA, C.CICCOSANTI, Y.HUANG, D.PATEL, R.XIAO, T.ACTON, J.EVERETT, G.T.MONTELIONE, NORTHEAST STRUCTURAL GENOMICS CONSORTIUM (NESG)
**Last author**	MONTELIONE
**Reference**	
**Reference authors**	
**Reference title**	
**Software listed**	AUTOASSIGN, AUTOSTRUCTURE, CNS, CYANA, PINE
**Spectrometer**	BRUKER, VARIAN (800 MHZ, 600 MHZ, 500 MHZ)

**Protein number**	71
**PDB code**	2LFI, doi:10.2210/pdb2LFI/pdb
**BMRB code**	17754, doi:10.13018/BMR17754
**PDB Header**	METAL BINDING PROTEIN
**Protein name**	A MUCBP DOMAIN (FRAGMENT 187-294) OF PROTEIN LBA1460 FROM LACTOBACILLUS ACIDOPHILUS
**Deposition date**	30.06.2011
**PDB title**	SOLUTION NMR STRUCTURE OF A MUCBP DOMAIN (FRAGMENT 187-294) OF THE PROTEIN LBA1460 FROM LACTOBACILLUS ACIDOPHILUS, NORTHEAST STRUCTURAL GENOMICS CONSORTIUM TARGET LAR80A
**PDB authors**	E.A.FELDMANN, T.A.RAMELOT, Y.YANG, H.LEE, C.CICCOSANTI, H.JANJUA, R.NAIR, T.B.ACTON, R.XIAO, J.K.EVERETT, J.H.PRESTEGARD, G.T.MONTELIONE, M.A.KENNEDY, NORTHEAST STRUCTURAL GENOMICS CONSORTIUM (NESG)
**Last author**	KENNEDY
**Reference**	
**Reference authors**	
**Reference title**	
**Software listed**	AUTOSTRUCTURE, CNS, CYANA, NMRPIPE, PDBSTAT, PINE_SERVER, PSVS, SPARKY, TOPSPIN, VNMR, X-PLOR_NIH
**Spectrometer**	BRUKER, VARIAN (850 MHZ, 600 MHZ)

**Protein number**	72
**PDB code**	2JQN, doi:10.2210/pdb2JQN/pdb
**BMRB code**	15281, doi:10.13018/BMR15281
**PDB Header**	STRUCTURAL GENOMICS
**Protein name**	CC0527 FROM CAULOBACTER CRESCENTUS
**Deposition date**	05.06.2007
**PDB title**	SOLUTION NMR STRUCTURE OF CC0527 FROM CAULOBACTER CRESCENTUS. NORTHEAST STRUCTURAL GENOMICS TARGET CCR55
**PDB authors**	J.M.ARAMINI, P.ROSSI, H.N.B.MOSELEY, D.WANG, C.NWOSU, K.CUNNINGHAM, L.MA, R.XIAO, J.LIU, M.C.BARAN, G.V.T.SWAPNA, T.B.ACTON, B.ROST, G.T.MONTELIONE, NORTHEAST STRUCTURAL GENOMICS CONSORTIUM (NESG)

**Last author**	MONTELIONE
**Reference**	
**Reference authors**	
**Reference title**	
**Software listed**	AUTOASSIGN, AUTOSTRUCTURE, CNS, NMRPIPE, PDBSTAT, PSVS, SPARKY, TOPSPIN, VNMR, X-PLOR
**Spectrometer**	BRUKER, VARIAN (800 MHZ, 600 MHZ)

**Protein number**	73
**PDB code**	2L7Q, doi:10.2210/pdb2L7Q/pdb
**BMRB code**	17370, doi:10.13018/BMR17370
**PDB Header**	STRUCTURAL GENOMICS, UNKNOWN FUNCTION
**Protein name**	CONJUGATE TRANSPOSON PROTEIN BVU_1572(27- 141) FROM BACTEROIDES VULGATUS
**Deposition date**	20.12.2010
**PDB title**	SOLUTION NMR STRUCTURE OF CONJUGATE TRANSPOSON PROTEIN BVU_1572(27- 141) FROM BACTEROIDES VULGATUS, NORTHEAST STRUCTURAL GENOMICS CONSORTIUM TARGET BVR155
**PDB authors**	Y.YANG, T.A.RAMELOT, J.R.CORT, D.WANG, C.CICCOSANTI, H.JANJUA, T.B.ACTON, R.XIAO, J.K.EVERETT, G.T.MONTELIONE, M.A.KENNEDY, NORTHEAST STRUCTURAL GENOMICS CONSORTIUM (NESG)
**Last author**	KENNEDY
**Reference**	PROTEINS 80, 667 (2012), doi:10.1002/PROT.23235
**Reference authors**	T.A.RAMELOT, Y.YANG, R.XIAO, T.B.ACTON, J.K.EVERETT, G.T.MONTELIONE, M.A.KENNEDY
**Reference title**	SOLUTION NMR STRUCTURE OF BT_0084, A CONJUGATIVE TRANSPOSON LIPOPROTEIN FROM BACTEROIDES THETAIOTAMICRON
**Software listed**	AUTOASSIGN, AUTOSTRUCTURE, CNS, CYANA, NMRPIPE, PDBSTAT, PINE_SERVER, PSVS, SPARKY, TOPSPIN, VNMR, X-PLOR_NIH
**Spectrometer**	BRUKER, VARIAN (850 MHZ, 600 MHZ)

**Protein number**	74
**PDB code**	2KFP, doi:10.2210/pdb2KFP/pdb
**BMRB code**	16186, doi:10.13018/BMR16186
**PDB Header**	STRUCTURAL GENOMICS, UNKNOWN FUNCTION
**Protein name**	PSPTO_3016 FROM PSEUDOMONAS SYRINGAE
**Deposition date**	24.02.2009
**PDB title**	SOLUTION NMR STRUCTURE OF PSPTO_3016 FROM PSEUDOMONAS SYRINGAE. NORTHEAST STRUCTURAL GENOMICS CONSORTIUM TARGET PSR293
**PDB authors**	E.A.FELDMANN, T.A.RAMELOT, L.ZHAO, K.HAMILTON, C.CICCOSANTI, R.XIAO, R.NAIR, J.K.EVERETT, G.SWAPNA, T.B.ACTON, B.ROST, G.T.MONTELIONE, M.A.KENNEDY, NORTHEAST STRUCTURAL GENOMICS CONSORTIUM (NESG)
**Last author**	KENNEDY
**Reference**	J.STRUCT.FUNCT.GENOM. 13, 155 (2012), doi:10.1007/S10969-012-9140-8
**Reference authors**	E.A.FELDMANN, J.SEETHARAMAN, T.A.RAMELOT, S.LEW, L.ZHAO, K.HAMILTON, C.CICCOSANTI, R.XIAO, T.B.ACTON, J.K.EVERETT, L.TONG, G.T.MONTELIONE, M.A.KENNEDY
**Reference title**	SOLUTION NMR AND X-RAY CRYSTAL STRUCTURES OF PSEUDOMONAS SYRINGAE PSPTO_3016 FROM PROTEIN DOMAIN FAMILY PF04237 (DUF419) ADOPT A "DOUBLE WING" DNA BINDING MOTIF
**Software listed**	AUTOASSIGN, AUTOSTRUCTURE, NMRPIPE, PSVS, SPARKY, TOPSPIN, VNMR, X-PLOR
**Spectrometer**	BRUKER, VARIAN (850 MHZ, 600 MHZ)

**Protein number**	75
**PDB code**	1SE9, doi:10.2210/pdb1SE9/pdb
**BMRB code**	6128, doi:10.13018/BMR6128
**PDB Header**	PLANT PROTEIN
**Protein name**	AT3G01050, A UBIQUITIN-FOLD PROTEIN FROM ARABIDOPSIS THALIANA
**Deposition date**	16.02.2004
**PDB title**	STRUCTURE OF AT3G01050, A UBIQUITIN-FOLD PROTEIN FROM ARABIDOPSIS THALIANA
**PDB authors**	B.F.VOLKMAN, B.L.LYTLE, F.C.PETERSON, CENTER FOR EUKARYOTIC STRUCTURAL GENOMICS (CESG)
**Last author**	PETERSON
**Reference**	NAT.METHODS 1, 149 (2004), doi:10.1038/NMETH716
**Reference authors**	D.A.VINAROV, B.L.LYTLE, F.C.PETERSON, E.M.TYLER, B.F.VOLKMAN, J.L.MARKLEY
**Reference title**	CELL-FREE PROTEIN PRODUCTION AND LABELING PROTOCOL FOR NMR-BASED STRUCTURAL PROTEOMICS
**Software listed**	CYANA, NMRPIPE, SPSCAN, X-PLOR_NIH, XEASY, XWINNMR
**Spectrometer**	BRUKER (600 MHZ)

**Protein number**	76
**PDB code**	2L3G, doi:10.2210/pdb2L3G/pdb
**BMRB code**	17192, doi:10.13018/BMR17192
**PDB Header**	SIGNALING PROTEIN
**Protein name**	CH DOMAIN OF RHO GUANINE NUCLEOTIDE EXCHANGE FACTOR 7 FROM HOMO SAPIENS
**Deposition date**	13.09.2010
**PDB title**	SOLUTION NMR STRUCTURE OF CH DOMAIN OF RHO GUANINE NUCLEOTIDE EXCHANGE FACTOR 7 FROM HOMO SAPIENS, NORTHEAST STRUCTURAL GENOMICS CONSORTIUM TARGET HR4495E
**PDB authors**	G.LIU, R.XIAO, H.JANJUA, T.B.ACTON, A.CICCOSANTI, R.SHASTRY, J.EVERETT, G.T.MONTELIONE, NORTHEAST STRUCTURAL GENOMICS CONSORTIUM (NESG)
**Last author**	MONTELIONE
**Reference**	

**Reference authors**	
**Reference title**	
**Software listed**	AUTOASSIGN, AUTOSTRUCTURE, CNS, CYANA, NMRPIPE, TALOS+, TOPSPIN, VNMRJ, XEASY
**Spectrometer**	BRUKER, VARIAN (800 MHZ, 600 MHZ)

**Protein number**	77
**PDB code**	2L3B, doi:10.2210/pdb2L3B/pdb
**BMRB code**	17176, doi:10.13018/BMR17176
**PDB Header**	STRUCTURAL GENOMICS, UNKNOWN FUNCTION
**Protein name**	BT_0084 LIPOPROTEIN FROM BACTEROIDES THETAIOTAOMICRON
**Deposition date**	10.09.2010
**PDB title**	SOLUTION NMR STRUCTURE OF THE BT_0084 LIPOPROTEIN FROM BACTEROIDES THETAIOTAOMICRON, NORTHEAST STRUCTURAL GENOMICS CONSORTIUM TARGET BTR376
**PDB authors**	T.A.RAMELOT, Y.YANG, D.WANG, C.CICCOSANTI, H.JANJUA, T.B.ACTON, R.XIAO, J.K.EVERETT, G.T.MONTELIONE, M.A.KENNEDY, NORTHEAST STRUCTURAL GENOMICS CONSORTIUM (NESG)
**Last author**	KENNEDY
**Reference**	PROTEINS 867 2012, doi:10.1002/PROT.23235
**Reference authors**	T.A.RAMELOT, Y.YANG, R.XIAO, T.B.ACTON, J.K.EVERETT, G.T.MONTELIONE, M.A.KENNEDY
**Reference title**	SOLUTION NMR STRUCTURE OF BT_0084, A CONJUGATIVE TRANSPOSON LIPOPROTEIN FROM BACTEROIDES THETAIOTAMICRON
**Software listed**	AUTOASSIGN, AUTOSTRUCTURE, CYANA, NMRPIPE, PDBSTAT, PSVS, SPARKY, TOPSPIN, VNMR, X-PLOR
**Spectrometer**	BRUKER, VARIAN (850 MHZ, 600 MHZ)

**Protein number**	78
**PDB code**	2LRH, doi:10.2210/pdb2LRH/pdb
**BMRB code**	18372, doi:10.13018/BMR18372
**PDB Header**	DE NOVO PROTEIN
**Protein name**	DE NOVO DESIGNED PROTEIN, P-LOOP NTPASE FOLD
**Deposition date**	30.03.2012
**PDB title**	SOLUTION NMR STRUCTURE OF DE NOVO DESIGNED PROTEIN, P-LOOP NTPASE FOLD, NORTHEAST STRUCTURAL GENOMICS CONSORTIUM TARGET OR137
**PDB authors**	G.LIU, N.KOGA, R.KOGA, R.XIAO, H.LEE, H.JANJUA, E.KOHAN, T.B.ACTON, J.K.EVERETT, D.BAKER, G.T.MONTELIONE, NORTHEAST STRUCTURAL GENOMICS CONSORTIUM (NESG)
**Last author**	MONTELIONE
**Reference**	
**Reference authors**	
**Reference title**	
**Software listed**	AUTOASSIGN, AUTOSTRUCTURE, CNS, CYANA, NMRPIPE, PALES, PSVS, REDCAT, SPARKY, TALOS+, TOPSPIN, VNMRJ, XEASY
**Spectrometer**	BRUKER, VARIAN (800 MHZ, 600 MHZ)

**Protein number**	79
**PDB code**	1VEE, doi:10.2210/pdb1VEE/pdb
**BMRB code**	5929, doi:10.13018/BMR5929
**PDB Header**	STRUCTURAL GENOMICS, UNKNOWN FUNCTION
**Protein name**	HYPOTHETICAL RHODANESE DOMAIN AT4G01050 FROM ARABIDOPSIS THALIANA
**Deposition date**	30.03.2004
**PDB title**	NMR STRUCTURE OF THE HYPOTHETICAL RHODANESE DOMAIN AT4G01050 FROM ARABIDOPSIS THALIANA
**PDB authors**	D.PANTOJA-UCEDA, B.LOPEZ-MENDEZ, S.KOSHIBA, M.INOUE, T.KIGAWA, T.TERADA, M.SHIROUZU, A.TANAKA, M.SEKI, K.SHINOZAKI, S.YOKOYAMA, P.GUNTERT, RIKEN STRUCTURAL GENOMICS/PROTEOMICS INITIATIVE (RSGI)
**Last author**	GUNTERT
**Reference**	PROTEIN SCI. 14, 224 (2005), doi:10.1110/PS.041138705
**Reference authors**	D.PANTOJA-UCEDA, B.LOPEZ-MENDEZ, S.KOSHIBA, M.INOUE, T.KIGAWA, T.TERADA, M.SHIROUZU, A.TANAKA, M.SEKI, K.SHINOZAKI, S.YOKOYAMA, P.GUNTERT
**Reference title**	SOLUTION STRUCTURE OF THE RHODANESE HOMOLOGY DOMAIN AT4G01050(175-295) FROM ARABIDOPSIS THALIANA
**Software listed**	CYANA, OPALP
**Spectrometer**	BRUKER (800 MHZ, 600 MHZ)

**Protein number**	80
**PDB code**	2K1G, doi:10.2210/pdb2K1G/pdb
**BMRB code**	15603, doi:10.13018/BMR15603
**PDB Header**	LIPOPROTEIN
**Protein name**	LIPOPROTEIN SPR FROM ESCHERICHIA COLI K12
**Deposition date**	03.03.2008
**PDB title**	SOLUTION NMR STRUCTURE OF LIPOPROTEIN SPR FROM ESCHERICHIA COLI K12. NORTHEAST STRUCTURAL GENOMICS TARGET ER541-37-162
**PDB authors**	J.M.ARAMINI, P.ROSSI, L.ZHAO, M.JIANG, M.MAGLAQUI, R.XIAO, J.LIU, M.C.BARAN, G.V.T.SWAPNA, Y.J.HUANG, T.B.ACTON, B.ROST, G.T.MONTELIONE, NORTHEAST STRUCTURAL GENOMICS CONSORTIUM (NESG)
**Last author**	MONTELIONE
**Reference**	BIOCHEMISTRY 47, 9715 (2008), doi:10.1021/BI8010779
**Reference authors**	J.M.ARAMINI, P.ROSSI, Y.J.HUANG, L.ZHAO, M.JIANG, M.MAGLAQUI, R.XIAO, J.LOCKE, R.NAIR, B.ROST, T.B.ACTON, M.INOUYE, G.T.MONTELIONE

**Reference title**	SOLUTION NMR STRUCTURE OF THE NLPC/P60 DOMAIN OF LIPOPROTEIN SPR FROM ESCHERICHIA COLI: STRUCTURAL EVIDENCE FOR A NOVEL CYSTEINE PEPTIDASE CATALYTIC TRIAD
**Software listed**	AUTOASSIGN, AUTOSTRUCTURE, CNS, CYANA, NMRPIPE, PDBSTAT, PSVS, SPARKY, TOPSPIN, VNMR
**Spectrometer**	BRUKER, VARIAN (800 MHZ, 600 MHZ)

**Protein number**	81
**PDB code**	2KKZ, doi:10.2210/pdb2KKZ/pdb
**BMRB code**	16376, doi:10.13018/BMR16376
**PDB Header**	ANTIVIRAL PROTEIN
**Protein name**	MONOMERIC W187R MUTANT OF A/UDORN NS1 EFFECTOR DOMAIN
**Deposition date**	29.06.2009
**PDB title**	SOLUTION NMR STRUCTURE OF THE MONOMERIC W187R MUTANT OF A/UDORN NS1 EFFECTOR DOMAIN. NORTHEAST STRUCTURAL GENOMICS TARGET OR8C[W187R]
**PDB authors**	J.M.ARAMINI, L.MA, H.LEE, L.ZHAO, K.CUNNINGHAM, C.CICCOSANTI, H.JANJUA, Y.FANG, R.XIAO, R.M.KRUG, G.T.MONTELIONE, NORTHEAST STRUCTURAL GENOMICS CONSORTIUM (NESG)
**Last author**	MONTELIONE
**Reference**	
**Reference authors**	
**Reference title**	
**Software listed**	AUTOASSIGN, AUTOSTRUCTURE, CNS, CYANA, MOLPROBITY, NMRPIPE, PDBSTAT, PSVS, SPARKY, TALOS, TOPSPIN
**Spectrometer**	BRUKER (800 MHZ, 600 MHZ)

**Protein number**	82
**PDB code**	1VDY, doi:10.2210/pdb1VDY/pdb
**BMRB code**	5928, doi:10.13018/BMR5928
**PDB Header**	STRUCTURAL GENOMICS, UNKNOWN FUNCTION
**Protein name**	HYPOTHETICAL ENTH-VHS DOMAIN AT3G16270 FROM ARABIDOPSIS THALIANA
**Deposition date**	25.03.2004
**PDB title**	NMR STRUCTURE OF THE HYPOTHETICAL ENTH-VHS DOMAIN AT3G16270 FROM ARABIDOPSIS THALIANA
**PDB authors**	B.LOPEZ-MENDEZ, D.PANTOJA-UCEDA, T.TOMIZAWA, S.KOSHIBA, T.KIGAWA, M.SHIROUZU, T.TERADA, M.INOUE, T.YABUKI, M.AOKI, E.SEKI, T.MATSUDA, H.HIROTA, M.YOSHIDA, A.TANAKA, T.OSANAI, M.SEKI, K.SHINOZAKI, S.YOKOYAMA, P.GUNTERT, RIKEN STRUCTURAL GENOMICS/PROTEOMICS INITIATIVE (RSGI)
**Last author**	GUNTERT
**Reference**	
**Reference authors**	
**Reference title**	
**Software listed**	CYANA, OPALP
**Spectrometer**	BRUKER (800 MHZ, 600 MHZ)

**Protein number**	83
**PDB code**	2KKL, doi:10.2210/pdb2KKL/pdb
**BMRB code**	16364, doi:10.13018/BMR16364
**PDB Header**	STRUCTURAL GENOMICS, UNKNOWN FUNCTION
**Protein name**	FHA DOMAIN OF MB1858 FROM MYCOBACTERIUM BOVIS
**Deposition date**	25.06.2009
**PDB title**	SOLUTION NMR STRUCTURE OF FHA DOMAIN OF MB1858 FROM MYCOBACTERIUM BOVIS. NORTHEAST STRUCTURAL GENOMICS CONSORTIUM TARGET MBR243C (24- 155)
**PDB authors**	Y.YANG, T.A.RAMELOT, D.WANG, E.L.FOOTE, M.JIANG, R.NAIR, B.ROST, G.SWAPNA, T.B.ACTON, R.XIAO, J.K.EVERETT, G.T.MONTELIONE, M.A.KENNEDY, NORTHEAST STRUCTURAL GENOMICS CONSORTIUM (NESG)
**Last author**	KENNEDY
**Reference**	
**Reference authors**	
**Reference title**	
**Software listed**	AUTOASSIGN, AUTOSTRUCTURE, CNS, NMRPIPE, PDBSTAT, PSVS, SPARKY, TOPSPIN, VNMR, X-PLOR
**Spectrometer**	BRUKER, VARIAN (850 MHZ, 600 MHZ)

**Protein number**	84
**PDB code**	2N4B, doi:10.2210/pdb2N4B/pdb
**BMRB code**	17611, doi:10.13018/BMR17611
**PDB Header**	STRUCTURAL GENOMICS, UNKNOWN FUNCTION
**Protein name**	RALSTONIA METALLIDURANS RMET_5065 DETERMINED BY COMBINING EVOLUTIONARY COUPLINGS (EC) AND SPARSE NMR DATA
**Deposition date**	17.06.2015
**PDB title**	EC-NMR STRUCTURE OF RALSTONIA METALLIDURANS RMET_5065 DETERMINED BY COMBINING EVOLUTIONARY COUPLINGS (EC) AND SPARSE NMR DATA. NORTHEAST STRUCTURAL GENOMICS CONSORTIUM TARGET CRR115
**PDB authors**	Y.TANG, Y.J.HUANG, T.A.HOPF, C.SANDER, D.MARKS, G.T.MONTELIONE, NORTHEAST STRUCTURAL GENOMICS CONSORTIUM (NESG)
**Last author**	MONTELIONE
**Reference**	NAT.METHODS 12, 751 (2015), doi:10.1038/NMETH.3455
**Reference authors**	Y.TANG, Y.J.HUANG, T.A.HOPF, C.SANDER, D.S.MARKS, G.T.MONTELIONE

**Reference title**	PROTEIN STRUCTURE DETERMINATION BY COMBINING SPARSE NMR DATA WITH EVOLUTIONARY COUPLINGS
**Software listed**	ASDP, CYANA, EC-NMR, EVFOLD-PLM, REDUCE, ROSETTA, TALOS+
**Spectrometer**	

**Protein number**	85
**PDB code**	2L8V, doi:10.2210/pdb2L8V/pdb
**BMRB code**	17429, doi:10.13018/BMR17429
**PDB Header**	PHOTOSYNTHESIS
**Protein name**	PHYCOBILISOME LINKER POLYPEPTIDE DOMAIN OF CPCC (20-153) FROM THERMOSYNECHOCOCCUS ELONGATUS
**Deposition date**	26.01.2011
**PDB title**	SOLUTION NMR STRUCTURE OF THE PHYCOBILISOME LINKER POLYPEPTIDE DOMAIN OF CPCC (20-153) FROM THERMOSYNECHOCOCCUS ELONGATUS, NORTHEAST STRUCTURAL GENOMICS CONSORTIUM TARGET TER219A
**PDB authors**	T.A.RAMELOT, Y.YANG, J.R.CORT, D.LEE, C.CICCOSANTI, K.HAMILTON, T.B.ACTON, R.XIAO, J.K.EVERETT, G.T.MONTELIONE, M.A.KENNEDY, NORTHEAST STRUCTURAL GENOMICS CONSORTIUM (NESG)
**Last author**	KENNEDY
**Reference**	
**Reference authors**	
**Reference title**	
**Software listed**	AUTOASSIGN, AUTOSTRUCTURE, CNS, CYANA, NMRPIPE, PDBSTAT, PINE_SERVER, PSVS, SPARKY, TOPSPIN, VNMR, X-PLOR_NIH
**Spectrometer**	BRUKER, VARIAN (850 MHZ, 600 MHZ)

**Protein number**	86
**PDB code**	2LGH, doi:10.2210/pdb2LGH/pdb
**BMRB code**	17809, doi:10.13018/BMR17809
**PDB Header**	STRUCTURAL GENOMICS, UNKNOWN FUNCTION
**Protein name**	AHSA1-LIKE PROTEIN AHA_2358 FROM AEROMONAS HYDROPHILA REFINED WITH NH RDCS
**Deposition date**	26.07.2011
**PDB title**	SOLUTION NMR STRUCTURE OF THE AHSA1-LIKE PROTEIN AHA_2358 FROM AEROMONAS HYDROPHILA REFINED WITH NH RDCS, NORTHEAST STRUCTURAL GENOMICS CONSORTIUM TARGET AHR99
**PDB authors**	T.A.RAMELOT, Y.YANG, H.LEE, D.WANG, C.CICCOSANTI, H.JANJUA, R.NAIR, T.B.ACTON, R.XIAO, J.K.EVERETT, J.H.PRESTEGARD, G.T.MONTELIONE, M.A.KENNEDY, NORTHEAST STRUCTURAL GENOMICS CONSORTIUM (NESG)
**Last author**	KENNEDY
**Reference**	
**Reference authors**	
**Reference title**	
**Software listed**	AUTOASSIGN, AUTOSTRUCTURE, CNS, CYANA, NMRPIPE, PDBSTAT, PINE_SERVER, PSVS, SPARKY, TOPSPIN, VNMR, X-PLOR_NIH
**Spectrometer**	BRUKER, VARIAN (850 MHZ, 600 MHZ)

**Protein number**	87
**PDB code**	2K1S, doi:10.2210/pdb2K1S/pdb
**BMRB code**	15683, doi:10.13018/BMR15683
**PDB Header**	LIPOPROTEIN
**Protein name**	FOLDED C-TERMINAL FRAGMENT OF YIAD FROM ESCHERICHIA COLI
**Deposition date**	14.03.2008
**PDB title**	SOLUTION NMR STRUCTURE OF THE FOLDED C-TERMINAL FRAGMENT OF YIAD FROM ESCHERICHIA COLI. NORTHEAST STRUCTURAL GENOMICS CONSORTIUM TARGET ER553
**PDB authors**	T.A.RAMELOT, L.ZHAO, K.HAMILTON, M.MAGLAQUI, R.XIAO, J.LIU, M.C.BARAN, G.SWAPNA, T.B.ACTON, B.ROST, G.T.MONTELIONE, M.A.KENNEDY, NORTHEAST STRUCTURAL GENOMICS CONSORTIUM (NESG)
**Last author**	KENNEDY
**Reference**	
**Reference authors**	
**Reference title**	
**Software listed**	AUTOASSIGN, AUTOSTRUCTURE, CNS, NMRPIPE, PSVS, SPARKY, TOPSPIN, VNMR, X-PLOR
**Spectrometer**	BRUKER, VARIAN (850 MHZ, 600 MHZ)

**Protein number**	88
**PDB code**	2M4F, doi:10.2210/pdb2M4F/pdb
**BMRB code**	19001, doi:10.13018/BMR19001
**PDB Header**	IMMUNE SYSTEM
**Protein name**	OUTER SURFACE PROTEIN E
**Deposition date**	05.02.2013
**PDB title**	SOLUTION STRUCTURE OF OUTER SURFACE PROTEIN E
**PDB authors**	A.BHATTACHARJEE, J.S.OEEMIG, R.KOLODZIEJCZYK, T.MERI, T.KAJANDER, H.IWAI, T.JOKIRANTA, A.GOLDMAN
**Last author**	GOLDMAN
**Reference**	J.BIOL.CHEM. 288, 18685 (2013), doi:10.1074/JBC.M113.459040
**Reference authors**	A.BHATTACHARJEE, J.S.OEEMIG, R.KOLODZIEJCZYK, T.MERI, T.KAJANDER, M.J.LEHTINEN, H.IWAI, T.S.JOKIRANTA, A.GOLDMAN
**Reference title**	STRUCTURAL BASIS FOR COMPLEMENT EVASION BY LYME DISEASE PATHOGEN BORRELIA BURGDORFERI
**Software listed**	AMBER, CCPNMR_ANALYSIS, CING, CYANA, NMRPIPE, VNMRJ

**Spectrometer**	VARIAN (800 MHZ, 600 MHZ)

**Protein number**	89
**PDB code**	2JXP, doi:10.2210/pdb2JXP/pdb
**BMRB code**	15568, doi:10.13018/BMR15568
**PDB Header**	LIPOPROTEIN
**Protein name**	UNCHARACTERIZED LIPOPROTEIN B FROM NITROSOMONAS EUROPAEA
**Deposition date**	27.11.2007
**PDB title**	SOLUTION NMR STRUCTURE OF UNCHARACTERIZED LIPOPROTEIN B FROM NITROSOMONAS EUROPAEA. NORTHEAST STRUCTURAL GENOMICS TARGET NER45A
**PDB authors**	P.ROSSI, D.WANG, H.JANJUA, L.OWENS, R.XIAO, M.C.BARAN, G.SWAPNA, T.B.ACTON, B.ROST, G.T.MONTELIONE, NORTHEAST STRUCTURAL GENOMICS CONSORTIUM (NESG)
**Last author**	MONTELIONE
**Reference**	
**Reference authors**	
**Reference title**	
**Software listed**	AUTOASSIGN, CNS, CYANA, MOLMOL, MOLPROBITY, NMRPIPE, PROCHECK, PSVS, RPF, SPARKY, TOPSPIN, XEASY
**Spectrometer**	BRUKER (800 MHZ)

**Protein number**	90
**PDB code**	2L06, doi:10.2210/pdb2L06/pdb
**BMRB code**	17031, doi:10.13018/BMR17031
**PDB Header**	PROTEIN BINDING
**Protein name**	PBS LINKER POLYPEPTIDE DOMAIN (FRAGMENT 254-400) OF PHYCOBILISOME LINKER PROTEIN APCE FROM SYNECHOCYSTIS SP. PCC 6803
**Deposition date**	30.06.2010
**PDB title**	SOLUTION NMR STRUCTURE OF THE PBS LINKER POLYPEPTIDE DOMAIN (FRAGMENT 254-400) OF PHYCOBILISOME LINKER PROTEIN APCE FROM SYNECHOCYSTIS SP. PCC 6803. NORTHEAST STRUCTURAL GENOMICS CONSORTIUM TARGET SGR209C
**PDB authors**	T.A.RAMELOT, Y.YANG, J.R.CORT, K.HAMILTON, C.CICCOSANTI, D.LEE, T.B.ACTON, R.XIAO, J.K.EVERETT, G.T.MONTELIONE, M.A.KENNEDY, NORTHEAST STRUCTURAL GENOMICS CONSORTIUM (NESG)
**Last author**	KENNEDY
**Reference**	
**Reference authors**	
**Reference title**	
**Software listed**	AUTOASSIGN, AUTOSTRUCTURE, CYANA, NMRPIPE, PDBSTAT, PSVS, SPARKY, TOPSPIN, VNMR, X-PLOR
**Spectrometer**	BRUKER, VARIAN (850 MHZ, 750 MHZ, 600 MHZ, 500 MHZ)

**Protein number**	91
**PDB code**	2LAH, doi:10.2210/pdb2LAH/pdb
**BMRB code**	17524, doi:10.13018/BMR17524
**PDB Header**	CELL CYCLE, APOPTOSIS
**Protein name**	MITOTIC CHECKPOINT SERINE/THREONINE-PROTEIN KINASE BUB1 N-TERMINAL DOMAIN FROM HOMO SAPIENS
**Deposition date**	14.03.2011
**PDB title**	SOLUTION NMR STRUCTURE OF MITOTIC CHECKPOINT SERINE/THREONINE-PROTEIN KINASE BUB1 N-TERMINAL DOMAIN FROM HOMO SAPIENS, NORTHEAST STRUCTURAL GENOMICS CONSORTIUM TARGET HR5460A (METHODS DEVELOPMENT)
**PDB authors**	G.LIU, R.XIAO, H.LEE, K.HAMILTON, T.B.ACTON, C.CICCOSANTI, J.K.EVERETT, R.T.SHASTRY, Y.J.HUANG, G.T.MONTELIONE, N.NORTHEAST STRUCTURAL GENOMICS CONSORTIUM, NORTHEAST STRUCTURAL GENOMICS CONSORTIUM (NESG)
**Last author**	MONTELIONE
**Reference**	
**Reference authors**	
**Reference title**	
**Software listed**	AUTOASSIGN, AUTOSTRUCTURE, CNS, CYANA, NMRPIPE, SPARKY, TALOS+, TOPSPIN, VNMRJ, XEASY
**Spectrometer**	BRUKER, VARIAN (800 MHZ, 600 MHZ)

**Protein number**	92
**PDB code**	2LAK, doi:10.2210/pdb2LAK/pdb
**BMRB code**	17530, doi:10.13018/BMR17530
**PDB Header**	STRUCTURE GENOMICS, UNKNOWN FUNCTION
**Protein name**	AHSA1-LIKE PROTEIN RHE_CH02687 (1-152) FROM RHIZOBIUM ETLI
**Deposition date**	16.03.2011
**PDB title**	SOLUTION NMR STRUCTURE OF THE AHSA1-LIKE PROTEIN RHE_CH02687 (1-152) FROM RHIZOBIUM ETLI, NORTHEAST STRUCTURAL GENOMICS CONSORTIUM TARGET RER242
**PDB authors**	Y.YANG, T.A.RAMELOT, J.R.CORT, D.WANG, C.CICCOSANTI, H.JANJUA, R.NAIR, B.ROST, T.B.ACTON, R.XIAO, J.K.EVERETT, G.T.MONTELIONE, M.A.KENNEDY, NORTHEAST STRUCTURAL GENOMICS CONSORTIUM (NESG)
**Last author**	KENNEDY
**Reference**	
**Reference authors**	
**Reference title**	
**Software listed**	AUTOASSIGN, AUTOSTRUCTURE, CNS, CYANA, NMRPIPE, PDBSTAT, PINE_SERVER, PSVS, SPARKY, TOPSPIN, VNMR, X-PLOR_NIH
**Spectrometer**	BRUKER, VARIAN (850 MHZ, 600 MHZ)

**Protein number**	93
**PDB code**	2L82, doi:10.2210/pdb2L82/pdb
**BMRB code**	17390, doi:10.13018/BMR17390
**PDB Header**	DE NOVO PROTEIN
**Protein name**	DE NOVO DESIGNED PROTEIN, P-LOOP NTPASE FOLD
**Deposition date**	31.12.2010
**PDB title**	SOLUTION NMR STRUCTURE OF DE NOVO DESIGNED PROTEIN, P-LOOP NTPASE FOLD, NORTHEAST STRUCTURAL GENOMICS CONSORTIUM TARGET OR32
**PDB authors**	G.LIU, N.KOGA, R.KOGA, R.XIAO, K.HAMILTON, H.JANJUA, S.TONG, T.B.ACTON, J.EVERETT, D.BAKER, G.T.MONTELIONE, NORTHEAST STRUCTURAL GENOMICS CONSORTIUM (NESG)
**Last author**	MONTELIONE
**Reference**	
**Reference authors**	
**Reference title**	
**Software listed**	AUTOASSIGN, AUTOSTRUCTURE, CNS, CYANA, NMRPIPE, PALES, PINE, REDCAT, SPARKY, TALOS+, TOPSPIN, VNMRJ, XEASY
**Spectrometer**	BRUKER, VARIAN (800 MHZ, 600 MHZ)

**Protein number**	94
**PDB code**	2M47, doi:10.2210/pdb2M47/pdb
**BMRB code**	18989, doi:10.13018/BMR18989
**PDB Header**	STRUCTURAL GENOMICS, UNKNOWN FUNCTION
**Protein name**	POLYKETIDE_CYC-LIKE PROTEIN CGL2372 FROM CORYNEBACTERIUM GLUTAMICUM
**Deposition date**	30.01.2013
**PDB title**	SOLUTION NMR STRUCTURE OF THE POLYKETIDE_CYC-LIKE PROTEIN CGL2372 FROM CORYNEBACTERIUM GLUTAMICUM, NORTHEAST STRUCTURAL GENOMICS CONSORTIUM TARGET CGR160
**PDB authors**	Y.YANG, T.A.RAMELOT, D.LEE, C.CICCOSANTI, A.SAPIN, H.JANJUA, R.NAIR, B.ROST, T.B.ACTON, R.XIAO, J.K.EVERETT, G.T.MONTELIONE, M.A.KENNEDY, NORTHEAST STRUCTURAL GENOMICS CONSORTIUM (NESG)
**Last author**	KENNEDY
**Reference**	
**Reference authors**	
**Reference title**	
**Software listed**	AUTOASSIGN, AUTOSTRUCTURE, CNS, CYANA, NMRPIPE, PINE, PSVS, SPARKY, TALOS+, TOPSPIN, VNMRJ, XEASY
**Spectrometer**	BRUKER, VARIAN (850 MHZ, 600 MHZ)

**Protein number**	95
**PDB code**	2K3A, doi:10.2210/pdb2K3A/pdb
**BMRB code**	15335, doi:10.13018/BMR15335
**PDB Header**	HYDROLASE
**Protein name**	STAPHYLOCOCCUS SAPROPHYTICUS CHAP (CYSTEINE, HISTIDINE-DEPENDENT AMIDOHYDROLASES/PEPTIDASES) DOMAIN PROTEIN
**Deposition date**	29.04.2008
**PDB title**	NMR SOLUTION STRUCTURE OF STAPHYLOCOCCUS SAPROPHYTICUS CHAP (CYSTEINE, HISTIDINE-DEPENDENT AMIDOHYDROLASES/PEPTIDASES) DOMAIN PROTEIN. NORTHEAST STRUCTURAL GENOMICS CONSORTIUM TARGET SYR11
**PDB authors**	P.ROSSI, J.M.ARAMINI, C.X.CHEN, C.NWOSU, K.C.CUNNINGHAM, L.A.OWENS, R.XIAO, J.LIU, M.C.BARAN, G.SWAPNA, T.B.ACTON, B.ROST, G.T.MONTELIONE, NORTHEAST STRUCTURAL GENOMICS CONSORTIUM (NESG)
**Last author**	MONTELIONE
**Reference**	PROTEINS 74, 515 (2008), doi:10.1002/PROT.22267
**Reference authors**	P.ROSSI, J.M.ARAMINI, R.XIAO, C.X.CHEN, C.NWOSU, L.A.OWENS, M.MAGLAQUI, R.NAIR, M.FISCHER, T.B.ACTON, B.HONIG, B.ROST, G.T.MONTELIONE
**Reference title**	STRUCTURAL ELUCIDATION OF THE CYS-HIS-GLU-ASN PROTEOLYTIC RELAY IN THE SECRETED CHAP DOMAIN ENZYME FROM THE HUMAN PATHOGEN STAPHYLOCOCCUS SAPROPHYTICUS
**Software listed**	AUTOASSIGN, AUTOSTRUCTURE, CNS, CYANA, MOLMOL, MOLPROBITY, NMRPIPE, PDBSTAT, PROCHECK, PROSA, PSVS, SPARKY, TOPSPIN, VERIFY3D, X-PLOR
**Spectrometer**	BRUKER (800 MHZ, 600 MHZ)

**Protein number**	96
**PDB code**	2M7U, doi:10.2210/pdb2M7U/pdb
**BMRB code**	19213, doi:10.13018/BMR19213
**PDB Header**	SIGNALING PROTEIN
**Protein name**	BLUE LIGHT-ABSORBING STATE OF TEPIXJ, AN ACTIVE CYANOBACTERIOCHROME DOMAIN
**Deposition date**	01.05.2013
**PDB title**	BLUE LIGHT-ABSORBING STATE OF TEPIXJ, AN ACTIVE CYANOBACTERIOCHROME DOMAIN
**PDB authors**	G.CORNILESCU, C.C.CORNILESCU, S.E.BURGIE, J.M.WALKER, J.L.MARKLEY, A.T.ULIJASZ, R.D.VIERSTRA
**Last author**	VIERSTRA
**Reference**	
**Reference authors**	
**Reference title**	
**Software listed**	NMRPIPE, PIPP, X-PLOR_NIH
**Spectrometer**	BRUKER, VARIAN (900 MHZ, 800 MHZ, 700 MHZ, 600 MHZ)

**Protein number**	97
**PDB code**	2B3W, doi:10.2210/pdb2B3W/pdb
**BMRB code**	6782, doi:10.13018/BMR6782
**PDB Header**	STRUCTURAL GENOMICS, UNKNOWN FUNCTION
**Protein name**	E.COLI PROTEIN YBIA
**Deposition date**	21.09.2005
**PDB title**	NMR STRUCTURE OF THE E.COLI PROTEIN YBIA, NORTHEAST STRUCTURAL GENOMICS TARGET ET24
**PDB authors**	T.A.RAMELOT, J.R.CORT, R.XIAO, L.Y.SHIH, T.B.ACTON, G.T.MONTELIONE, M.A.KENNEDY, NORTHEAST STRUCTURAL GENOMICS CONSORTIUM (NESG)
**Last author**	KENNEDY
**Reference**	
**Reference authors**	
**Reference title**	
**Software listed**	AUTOSTRUCTURE, CNS, NMRPIPE, SPARKY, VNMR, X-PLOR
**Spectrometer**	VARIAN (800 MHZ, 750 MHZ, 600 MHZ)

**Protein number**	98
**PDB code**	(KRAS4B)
**BMRB code**	
**PDB Header**	
**Protein name**	
**Deposition date**	
**PDB title**	
**PDB authors**	
**Last author**	
**Reference**	
**Reference authors**	
**Reference title**	
**Software listed**	
**Spectrometer**	

**Protein number**	99
**PDB code**	2G0Q, doi:10.2210/pdb2G0Q/pdb
**BMRB code**	7007, doi:10.13018/BMR7007
**PDB Header**	STRUCTURAL GENOMICS, UNKNOWN FUNCTION
**Protein name**	AT5G39720.1 FROM ARABIDOPSIS THALIANA
**Deposition date**	13.02.2006
**PDB title**	SOLUTION STRUCTURE OF AT5G39720.1 FROM ARABIDOPSIS THALIANA
**PDB authors**	B.F.VOLKMAN, F.C.PETERSON, B.L.LYTLE, CENTER FOR EUKARYOTIC STRUCTURAL GENOMICS (CESG)
**Last author**	LYTLE
**Reference**	ACTA CRYSTALLOGR.,SECT.F 62, 490 (2006), doi:10.1107/S1744309106015946
**Reference authors**	B.L.LYTLE, F.C.PETERSON, E.M.TYLER, C.L.NEWMAN, D.A.VINAROV, J.L.MARKLEY, B.F.VOLKMAN
**Reference title**	SOLUTION STRUCTURE OF ARABIDOPSIS THALIANA PROTEIN AT5G39720.1, A MEMBER OF THE AIG2-LIKE PROTEIN FAMILY
**Software listed**	GARANT, NMRPIPE, SPSCAN, X-PLOR_NIH, XEASY, XWINNMR
**Spectrometer**	BRUKER (600 MHZ)

**Protein number**	100
**PDB code**	2LF2, doi:10.2210/pdb2LF2/pdb
**BMRB code**	17736, doi:10.13018/BMR17736
**PDB Header**	STRUCTURAL GENOMICS, UNKNOWN FUNCTION
**Protein name**	AHSA1-LIKE PROTEIN CHU_1110 FROM CYTOPHAGA HUTCHINSONII
**Deposition date**	28.06.2011
**PDB title**	SOLUTION NMR STRUCTURE OF THE AHSA1-LIKE PROTEIN CHU_1110 FROM CYTOPHAGA HUTCHINSONII, NORTHEAST STRUCTURAL GENOMICS CONSORTIUM TARGET CHR152
**PDB authors**	Y.YANG, T.A.RAMELOT, D.LEE, C.CICCOSANTI, T.B.ACTON, R.XIAO, J.K.EVERETT, G.T.MONTELIONE, M.A.KENNEDY, NORTHEAST STRUCTURAL GENOMICS CONSORTIUM (NESG)
**Last author**	KENNEDY
**Reference**	
**Reference authors**	
**Reference title**	
**Software listed**	AUTOASSIGN, AUTOSTRUCTURE, CNS, CYANA, NMRPIPE, PDBSTAT, PINE_SERVER, PSVS, SPARKY, TOPSPIN, VNMR, X-PLOR_NIH
**Spectrometer**	BRUKER, VARIAN (850 MHZ, 600 MHZ)

Supplementary Table 4 Results of automated structure determination of 100 proteins.

ID	Protein	Chemical shift assignment		Ramachandran plot statistics				Structure calculation and comparison to PDB reference				
		Backbone accuracy [%]	Side-chain accuracy [%]	Most favoured [%]	Additionally allowed [%]	Generously allowed [%]	Disallowed [%]	Number of distance restraints	CYANA target function [Å^2]	Residue range for RMSD calculation	Backbone RMSD to reference [Å]	Side-chain RMSD to reference [Å]
1	6SVC	97.52	83.97	96.40	3.60	0.00	0.00	1066	1.80	7-29	0.83	1.53
2	2JVD	99.57	92.94	89.80	10.20	0.00	0.00	1182	0.30	4-37	0.71	1.29
3	2K57	99.62	95.38	88.20	11.80	0.00	0.00	1176	0.35	5-52	0.71	1.22
4	6SOW	88.97	78.57	87.70	12.30	0.00	0.00	1111	2.32	8-55	1.16	2.04
5	2LX7	98.54	87.84	91.40	8.60	0.00	0.00	484	0.32	5-59	1.41	2.02
6	2MA6	98.94	89.36	67.40	30.00	2.60	0.00	953	3.25	10-57	1.56	2.09
7	2JRM	97.21	92.14	87.30	12.60	0.00	0.10	1312	0.50	6-47	1.43	2.55
8	1YEZ	99.06	90.95	89.10	10.90	0.00	0.00	1063	0.58	15-25,29-66	0.73	1.20
9	2L9R	97.93	90.44	89.20	10.80	0.00	0.00	1322	0.37	13-56	0.59	1.18
10	2K52	97.17	93.54	91.00	9.00	0.00	0.00	2018	2.68	7-70	1.10	1.81
11	2KRS	98.47	96.18	88.00	12.00	0.00	0.00	1378	0.35	2-61	1.26	1.50
12	2K53	98.53	95.30	93.40	6.60	0.00	0.00	1183	0.60	8-28,39-66	0.88	1.29
13	2JT1	88.66	90.75	91.60	8.40	0.00	0.00	1240	0.11	5-57,66-69	0.94	1.39
14	2JVO	96.64	76.42	77.90	22.00	0.10	0.00	2263	10.44	6-71	1.77	2.49
15	2ERR	96.36	78.40	87.30	12.70	0.00	0.00	1578	13.61	2-75	2.09	3.16
16	2L1P	96.29	87.04	83.90	13.40	2.60	0.00	2075	2.10	19-78	2.13	2.94
17	2LN3	98.37	92.52	87.20	12.70	0.10	0.00	1947	1.15	6-72	0.89	1.34
18	2HEQ	94.48	84.63	80.40	17.80	1.80	0.00	1822	3.20	17-20,34-68	0.60	1.48
19	2KK8	99.18	90.26	84.40	15.60	0.00	0.00	1818	2.24	10-82	1.25	2.04
20	2KD0	97.24	91.96	85.80	14.20	0.00	0.00	2215	4.43	13-81	1.37	2.02
21	2LML	97.44	91.71	91.30	8.70	0.00	0.00	1197	0.67	3-78	1.53	1.90
22	2K3D	98.96	89.76	85.90	14.10	0.00	0.00	1922	2.00	2-81	1.44	2.13
23	2LK2	96.08	86.08	82.90	17.10	0.00	0.00	1152	0.94	14-65	1.42	1.98
24	MH04	98.58	88.57	89.00	11.00	0.00	0.00	1522	0.86	6-87	1.57	2.35
25	1PQX	99.03	86.51	81.70	16.00	2.30	0.00	1977	10.18	13-19,28-34,38-65,70-81	1.40	2.02
26	2L33	98.68	91.76	79.70	20.10	0.20	0.00	2092	1.37	19-36,46-79	0.79	1.32
27	2KZV	95.84	81.67	85.50	14.50	0.00	0.00	1440	0.41	9-80	2.62	3.13
28	2KCT	96.59	93.42	83.20	16.70	0.10	0.10	2431	4.45	11-37,44-83	0.77	1.32
29	2MDR	93.17	82.89	85.00	13.90	0.80	0.30	2307	3.06	9-89	1.72	3.04
30	2FB7	95.95	81.18	79.90	20.00	0.10	0.00	1218	1.51	20-52,74-87	1.94	2.52
31	2MB0	97.69	88.46	83.50	16.50	0.00	0.00	2701	9.63	8-41,49-85	1.11	1.81
32	2L05	99.50	91.56	85.10	14.10	0.10	0.80	2336	2.07	19-89	0.74	1.23
33	2KJR	99.76	91.62	79.90	20.10	0.00	0.00	2109	1.04	15-23,28-94	1.02	1.35
34	2M5O	92.48	90.20	77.40	22.60	0.10	0.00	2476	7.18	17-91	1.08	1.59
35	MDM2	98.08	84.88	87.40	12.30	0.30	0.00	1452	1.29	9-92	1.24	1.87
36	2LNA	99.30	90.78	78.80	19.90	1.20	0.00	2569	4.06	16-49,59-94	0.86	1.45
37	2LA6	98.78	92.60	83.60	16.40	0.00	0.00	2370	6.62	15-97	0.81	1.34
38	6FIP	97.86	79.15	85.20	14.80	0.10	0.00	2381	17.06	11-93	2.05	2.88
39	2LEA	96.17	83.33	75.40	24.50	0.10	0.00	2542	7.98	15-45,55-88	1.45	2.00
40	2LL8	98.04	91.42	89.90	10.10	0.00	0.00	2237	2.64	4-90	1.42	1.79
41	2KPN	96.95	91.16	79.80	20.00	0.20	0.00	2287	2.33	12-84	0.97	1.58
42	2K0M	96.87	92.08	88.00	12.00	0.00	0.10	1508	0.69	7-70,76-93	1.60	2.09
43	2K5V	99.37	94.25	89.20	10.80	0.00	0.00	1974	1.10	2-29,38-78,83-94	0.94	1.50
44	2MQL	83.59	70.55	72.10	25.10	2.80	0.10	2836	22.17	15-84	0.98	1.56
45	2K75	98.77	88.36	90.10	9.90	0.10	0.00	1881	0.77	3-92	1.65	1.98
46	2LTM	99.31	92.79	85.60	14.40	0.00	0.00	2696	2.68	14-99	0.67	1.46
47	2KOB	88.18	82.29	84.90	15.10	0.00	0.00	1840	2.66	3-93	2.24	2.93
48	2KHD	97.29	87.63	87.90	12.00	0.10	0.00	1059	0.04	31-97	1.87	2.22
49	2RN7	94.35	81.02	84.10	15.80	0.10	0.00	459	0.54	10-55	1.93	2.26
50	2LXU	99.40	91.33	82.90	17.10	0.00	0.00	2115	1.40	9-95	1.19	1.81

Supplementary Table 4 continued

ID	Protein	Chemical shift assignment		Ramachandran plot statistics				Structure calculation and comparison to PDB reference				
		Backbone accuracy [%]	Side-chain accuracy [%]	Most favored [%]	Additionally allowed [%]	Generously allowed [%]	Disallowed [%]	Number of distance restraints	CYANA target function [Å^2]	Residue range for RMSD calculation	Backbone RMSD to reference [Å]	Side-chain RMSD to reference [Å]
51	2KIF	94.43	87.78	81.80	18.20	0.10	0.00	3056	3.64	3-97	0.89	1.51
52	2KBN	97.98	88.49	88.80	11.20	0.00	0.00	2347	0.45	5-29,34-54,58-76,81-94	0.92	1.46
53	2MK2	99.58	91.97	85.50	14.40	0.10	0.00	2049	2.62	14-108	1.56	2.13
54	2K50	97.39	89.74	84.40	15.40	0.00	0.20	2157	0.86	10-34,42-85,92-105	1.00	1.57
55	2KL5	79.41	68.59	72.40	25.50	2.20	0.00	1878	5.90	12-53,58-66,76-86,93-99	2.58	3.38
56	2LTA	95.17	81.57	91.10	8.90	0.00	0.00	1989	0.73	4-98	2.39	2.90
57	2KIW	93.99	80.94	87.50	12.50	0.00	0.00	1964	1.10	2-81	1.59	2.08
58	2LVB	89.27	84.11	92.70	7.30	0.00	0.00	1711	0.86	3-102	1.56	2.17
59	2LND	95.52	88.24	92.90	7.10	0.00	0.00	2605	1.29	3-48,52-101	0.85	1.44
60	1WQU	98.05	86.80	79.60	20.20	0.20	0.10	3785	9.51	8-106	0.99	1.53
61	2KL6	98.64	93.52	86.00	14.00	0.00	0.00	2752	1.99	6-106	0.87	1.38
62	6GT7	96.93	79.31	89.10	10.90	0.00	0.00	3069	4.21	7-30,40-87,94-113	1.39	2.00
63	2JN8	95.45	89.68	83.00	16.80	0.30	0.00	1863	1.12	12-26,31-109	1.83	2.36
64	2K5D	99.25	92.40	75.50	24.30	0.20	0.00	2425	1.44	19-50,55-84,98-107	1.47	1.87
65	2KD1	98.31	90.36	83.60	16.30	0.00	0.10	2550	6.35	7-89	1.99	3.02
66	2LTL	96.44	90.46	89.00	11.00	0.00	0.00	1667	1.07	19-35,39-41,46-110	2.37	2.85
67	2KVO	98.17	90.00	79.20	20.80	0.00	0.00	2112	3.88	3-23,28-103	1.87	2.18
68	1T0Y	97.27	85.04	76.10	23.80	0.10	0.00	2521	1.60	4-83	1.27	1.96
69	2KCD	91.30	79.24	83.40	16.60	0.00	0.00	1421	10.14	3-108	3.13	3.59
70	2KRT	97.01	82.72	81.80	18.20	0.00	0.00	1844	0.83	6-114	2.09	2.73
71	2LFI	89.30	76.07	85.90	14.10	0.00	0.00	1661	0.80	2-104	2.42	2.89
72	2JQN	97.81	90.68	88.50	11.50	0.00	0.00	1904	1.09	3-111	1.52	1.86
73	2L7Q	96.11	82.62	83.80	16.20	0.00	0.00	2125	1.74	12-37,46-101,105-114	1.57	2.15
74	2KFP	97.39	79.51	82.30	17.70	0.00	0.00	2650	8.46	3-115	2.23	2.89
75	1SE9	88.55	82.13	76.80	22.80	0.30	0.10	1507	1.85	17-84,94-101	2.10	2.59
76	2L3G	98.04	93.19	83.90	16.10	0.00	0.00	2870	2.34	13-123	1.28	1.95
77	2L3B	93.97	86.19	83.20	16.80	0.00	0.00	2185	0.81	14-38,45-113	1.05	1.63
78	2LRH	94.91	77.45	90.60	9.40	0.00	0.00	3261	5.26	3-122	2.30	2.75
79	1VEE	97.81	90.30	78.20	21.70	0.10	0.00	3627	5.14	6-123	1.03	1.41
80	2K1G	97.22	92.18	79.00	20.90	0.00	0.00	3366	14.38	5-78,83-122	1.05	1.68
81	2KKZ	96.50	87.83	82.40	16.80	0.80	0.00	2513	2.36	5-80,86-118	1.47	2.12
82	1VDY	95.48	88.37	84.60	15.40	0.00	0.00	3833	9.13	9-102,113-128	0.95	1.43
83	2KKL	94.06	80.60	71.40	28.40	0.10	0.00	2127	2.09	33-90,96-125	1.26	2.09
84	2N4B	98.94	90.25	85.00	14.50	0.60	0.00	3659	10.49	2-26,40-54,66-134	1.14	1.95
85	2L8V	93.82	76.58	85.00	15.00	0.00	0.00	1989	2.82	4-22,37-65,73-129	2.79	3.35
86	2LGH	98.03	88.92	83.50	16.40	0.00	0.00	2831	3.18	2-109,113-135	2.43	2.70
87	2K1S	98.83	92.02	86.50	13.50	0.00	0.00	2811	2.63	3-140	1.83	2.05
88	2M4F	90.80	81.83	82.30	16.90	0.80	0.00	3427	6.07	23-46,51-57,63-94,103-114,120-129,136-148	1.11	1.75
89	2JXP	97.08	89.62	86.50	13.50	0.00	0.00	2279	0.91	16-144	2.58	2.91
90	2L06	96.46	84.28	84.50	15.40	0.10	0.00	3374	13.42	15-38,45-141	1.57	2.05
91	2LAH	93.23	85.67	84.90	15.10	0.00	0.00	3573	10.34	14-25,33-158	1.71	2.22
92	2LAK	92.15	78.69	81.30	18.00	0.70	0.00	2180	4.21	10-37,68-77,93-139	1.54	1.82
93	2L82	97.87	81.05	85.40	14.40	0.20	0.00	3596	3.72	3-151	3.55	3.86
94	2M47	92.86	80.32	89.20	10.80	0.00	0.00	1930	1.46	5-25,40-56,66-156	4.72	5.42
95	2K3A	96.79	84.40	72.30	27.60	0.10	0.00	1680	0.81	57-102,108-127,138-153	0.99	1.34
96	2M7U	85.70	73.47	79.50	19.10	1.40	0.00	3243	15.64	12-151	2.14	2.96
97	2B3W	93.39	80.09	81.70	16.70	0.90	0.80	4315	23.41	16-162	2.67	3.37
98	KRAS4B	98.00	76.81	89.00	11.00	0.00	0.00	4678	10.89	4-24,37-59,66-163	1.60	2.30
99	2G0Q	93.99	84.33	79.30	20.30	0.00	0.40	3113	4.53	18-54,60-126	2.38	3.03
100	2LF2	97.56	85.86	86.10	13.80	0.10	0.00	3421	4.30	6-44,53-69,76-105,111-165	2.68	3.09

Residue ranges for RMSD calculation were determined by CYRANGE applied to the region between the first residue of the first secondary structure element and the last residue of the final secondary structure element of the reference PDB structure.

Supplementary Table 5 Structure accuracy prediction. Actual and predicted backbone RMSD between ARTINA and reference PDB structures. See Supplementary Figure 2 for details.

ID	Protein	RMSD to reference (Å)	Predicted RMSD to reference (Å)	Difference (Å)	ID	Protein	RMSD to reference (Å)	Predicted RMSD to reference (Å)	Difference (Å)
1	6SVC	0.83	1.67	0.84	51	2KIF	0.89	1.00	0.11
2	2JVD	0.71	0.56	-0.15	52	2KBN	0.92	1.00	0.08
3	2K57	0.71	1.00	0.29	53	2MK2	1.56	1.78	0.22
4	6SOW	1.16	2.44	1.28	54	2K50	1.00	1.00	0.00
5	2LX7	1.41	3.22	1.81	55	2KL5	2.58	2.11	-0.47
6	2MA6	1.56	2.00	0.44	56	2LTA	2.39	2.22	-0.17
7	2JRM	1.43	1.33	-0.10	57	2KIW	1.59	1.22	-0.37
8	1YEZ	0.73	0.78	0.05	58	2LVB	1.56	1.11	-0.45
9	2L9R	0.59	0.67	0.08	59	2LND	0.85	1.11	0.26
10	2K52	1.10	1.00	-0.10	60	1WQU	0.99	1.00	0.01
11	2KRS	1.26	0.78	-0.48	61	2KL6	0.87	1.33	0.46
12	2K53	0.88	0.78	-0.10	62	6GT7	1.39	1.33	-0.06
13	2JT1	0.94	0.67	-0.27	63	2JN8	1.83	1.67	-0.16
14	2JVO	1.77	0.78	-0.99	64	2K5D	1.47	1.67	0.20
15	2ERR	2.09	2.78	0.69	65	2KD1	1.99	2.78	0.79
16	2L1P	2.13	1.33	-0.80	66	2LTL	2.37	3.89	1.52
17	2LN3	0.89	0.89	-0.00	67	2KVO	1.87	2.22	0.35
18	2HEQ	0.60	0.89	0.29	68	1T0Y	1.27	1.00	-0.27
19	2KK8	1.25	1.56	0.31	69	2KCD	3.13	3.00	-0.13
20	2KD0	1.37	0.78	-0.59	70	2KRT	2.09	1.89	-0.20
21	2LML	1.53	1.11	-0.42	71	2LFI	2.42	3.11	0.69
22	2K3D	1.44	2.00	0.56	72	2JQN	1.52	1.67	0.15
23	2LK2	1.42	0.67	-0.75	73	2L7Q	1.57	1.22	-0.35
24	MH04	1.57	2.00	0.43	74	2KFP	2.23	2.33	0.10
25	1PQX	1.40	0.56	-0.84	75	1SE9	2.10	2.00	-0.10
26	2L33	0.79	0.67	-0.12	76	2L3G	1.28	2.00	0.72
27	2KZV	2.62	1.89	-0.73	77	2L3B	1.05	1.33	0.28
28	2KCT	0.77	1.33	0.56	78	2LRH	2.30	3.00	0.70
29	2MDR	1.72	1.67	-0.05	79	1VEE	1.03	0.56	-0.47
30	2FB7	1.94	1.56	-0.38	80	2K1G	1.05	1.00	-0.05
31	2MB0	1.11	1.00	-0.11	81	2KKZ	1.47	2.00	0.53
32	2L05	0.74	0.89	0.15	82	1VDY	0.95	1.00	0.05
33	2KJR	1.02	0.89	-0.13	83	2KKL	1.26	1.11	-0.15
34	2M5O	1.08	1.11	0.03	84	2N4B	1.14	1.00	-0.14
35	MDM2	1.24	1.11	-0.13	85	2L8V	2.79	2.56	-0.23
36	2LNA	0.86	1.00	0.14	86	2LGH	2.43	2.78	0.35
37	2LA6	0.81	0.78	-0.03	87	2K1S	1.83	2.00	0.17
38	6FIP	2.05	3.00	0.95	88	2M4F	1.11	1.33	0.22
39	2LEA	1.45	0.89	-0.56	89	2JXP	2.58	3.33	0.75
40	2LL8	1.42	1.22	-0.20	90	2L06	1.57	1.67	0.10
41	2KPN	0.97	1.00	0.03	91	2LAH	1.71	2.44	0.73
42	2K0M	1.60	1.22	-0.38	92	2LAK	1.54	1.33	-0.21
43	2K5V	0.94	0.89	-0.05	93	2L82	3.55	2.67	-0.88
44	2MQL	0.98	0.33	-0.65	94	2M47	4.72	3.11	-1.61
45	2K75	1.65	1.11	-0.54	95	2K3A	0.99	1.33	0.34
46	2LTM	0.67	1.00	0.33	96	2M7U	2.14	2.56	0.42
47	2KOB	2.24	1.78	-0.46	97	2B3W	2.67	2.89	0.22
48	2KHD	1.87	2.44	0.57	98	KRAS4B	1.60	1.22	-0.38
49	2RN7	1.93	2.33	0.40	99	2G0Q	2.38	2.44	0.06
50	2LXU	1.19	1.11	-0.08	100	2LF2	2.68	2.33	-0.35

Supplementary Table 6 ANSURR structure evaluation scores. Correlation and RMSD scores given are the average of the corresponding scores calculated by ANSURR version 2.0.55 for the individual conformers of the structure calculated by ARTINA and the reference structure in the PDB, respectively.

ID	Protein	ARTINA		Reference	
		Correlation score (%)	RMSD score (%)	Correlation score (%)	RMSD score (%)
1	6SVC	18.6	34.6	21.4	12.2
2	2JVD	72.0	83.5	94.5	74.2
3	2K57	16.3	49.3	18.3	51.0
4	6SOW	44.7	63.2	60.6	84.5
5	2LX7	28.7	4.5	35.1	19.3
6	2MA6	21.8	2.4	61.9	70.7
7	2JRM	88.7	90.6	75.8	63.5
8	1YEZ	49.1	71.0	49.9	76.5
9	2L9R	92.0	33.0	77.4	90.9
10	2K52	4.1	7.4	23.5	81.4
11	2KRS	43.5	49.7	19.7	6.4
12	2K53	60.4	39.6	59.5	77.2
13	2JT1	77.1	24.1	93.1	71.7
14	2JVO	1.4	55.1	10.3	88.6
15	2ERR	29.4	34.9	25.8	86.7
16	2L1P	91.4	50.4	77.1	40.1
17	2LN3	64.7	89.6	56.9	85.4
18	2HEQ	82.3	63.0	91.8	69.5
19	2KK8	41.4	16.1	23.9	52.1
20	2KD0	59.1	43.1	17.1	40.5
21	2LML	36.7	37.4	73.9	87.9
22	2K3D	36.2	51.6	52.1	32.9
23	2LK2	96.7	36.7	97.7	44.1
24	MH04	51.3	24.4	18.1	25.5
25	1PQX	33.9	25.2	72.7	13.2
26	2L33	80.0	46.3	89.5	64.8
27	2KZV	89.8	45.8	64.2	11.8
28	2KCT	85.0	48.8	88.3	57.9
29	2MDR	38.6	57.4	36.0	36.1
30	2FB7	73.8	10.2	44.8	18.4
31	2MB0	49.4	52.1	43.1	56.9
32	2L05	68.9	45.1	54.9	62.5
33	2KJR	49.1	32.9	31.9	7.3
34	2M5O	71.0	42.8	92.5	91.5
35	MDM2	64.3	26.0	65.1	17.3
36	2LNA	73.3	43.8	69.9	64.9
37	2LA6	45.6	31.4	76.6	79.2
38	6FIP	54.9	33.2	57.3	44.0
39	2LEA	39.1	16.1	30.8	34.1
40	2LL8	14.4	77.9	45.3	90.4
41	2KPN	92.8	26.5	65.6	33.3
42	2K0M	24.0	45.8	24.9	72.2
43	2K5V	33.7	7.6	33.2	51.4
44	2MQL	40.8	36.4	61.9	21.0
45	2K75	15.2	9.4	28.7	20.0
46	2LTM	43.5	56.1	20.7	67.1
47	2KOB	63.3	32.6	80.9	75.2
48	2KHD	79.5	19.3	77.2	23.4
49	2RN7	91.1	24.1	88.6	41.7
50	2LXU	79.4	42.2	68.9	77.7

ID	Protein	ARTINA		Reference	
		Correlation score (%)	RMSD score (%)	Correlation score (%)	RMSD score (%)
51	2KIF	26.4	58.4	53.6	85.9
52	2KBN	44.6	20.8	54.0	15.3
53	2MK2	72.2	31.2	64.9	46.1
54	2K50	46.9	15.3	65.5	65.2
55	2KL5	68.2	20.4	31.4	66.0
56	2LTA	21.3	45.9	38.0	92.7
57	2KIW	71.4	78.2	94.2	38.9
58	2LVB	30.2	70.1	51.7	96.3
59	2LND	43.0	84.7	55.3	96.7
60	1WQU	29.7	36.7	7.1	38.3
61	2KL6	42.1	18.9	31.9	55.5
62	6GT7	14.1	76.4	27.9	67.8
63	2JN8	67.1	47.5	90.5	47.1
64	2K5D	80.2	27.0	77.9	42.9
65	2KD1	71.5	35.6	65.2	71.3
66	2LTL	61.4	8.6	78.7	60.0
67	2KVO	27.3	40.1	68.5	22.2
68	1T0Y	95.3	42.3	56.9	64.6
69	2KCD	12.6	16.1	50.2	20.0
70	2KRT	28.0	29.6	23.1	62.7
71	2LFI	22.8	3.9	56.4	75.9
72	2JQN	5.1	35.1	36.7	40.7
73	2L7Q	83.5	9.7	60.5	51.7
74	2KFP	43.5	34.5	47.2	12.9
75	1SE9	78.1	11.5	82.0	44.6
76	2L3G	68.7	32.2	74.8	76.9
77	2L3B	49.2	12.6	68.2	59.6
78	2LRH	10.3	88.2	25.1	95.1
79	1VEE	0.9	37.6	1.1	50.5
80	2K1G	35.5	64.0	77.0	73.8
81	2KKZ	74.2	23.9	71.3	91.1
82	1VDY	3.7	26.9	5.7	50.8
83	2KKL	55.1	29.9	46.8	6.5
84	2N4B	22.1	23.3	48.0	62.4
85	2L8V	31.6	31.9	23.7	39.4
86	2LGH	48.4	36.5	65.8	85.1
87	2K1S	57.1	48.9	58.2	77.1
88	2M4F	81.5	26.5	76.9	46.5
89	2JXP	48.1	12.7	52.1	46.6
90	2L06	49.8	57.0	52.9	49.7
91	2LAH	24.8	63.1	19.8	92.2
92	2LAK	59.0	11.9	47.3	6.1
93	2L82	24.4	91.0	35.8	98.5
94	2M47	13.8	8.3	36.6	60.7
95	2K3A	81.0	28.0	74.7	61.0
96	2M7U	58.8	33.2	37.1	13.6
97	2B3W	73.2	24.4	55.9	55.5
98	KRAS4B	3.8	43.9	2.8	40.8
99	2G0Q	87.6	27.6	34.2	68.4
100	2LF2	31.8	29.4	28.6	35.2

Supplementary Table 7 RPF structure evaluation scores.

ID	Protein	Recall	Precision	F-measure	DP-score
1	6SVC	0.91	0.87	0.89	0.70
2	2JVD	0.93	0.93	0.93	0.77
3	2K57	0.94	0.86	0.90	0.75
4	6SOW	0.82	0.84	0.83	0.64
5	2LX7	0.86	0.84	0.85	0.52
6	2MA6	0.83	0.83	0.83	0.55
7	2JRM	0.86	0.88	0.87	0.67
8	1YEZ	0.85	0.82	0.83	0.65
9	2L9R	0.93	0.86	0.89	0.83
10	2K52	0.91	0.82	0.86	0.75
11	2KRS	0.93	0.90	0.92	0.78
12	2K53	0.89	0.89	0.89	0.66
13	2JT1	0.92	0.88	0.90	0.72
14	2JVO	0.81	0.90	0.85	0.70
15	2ERR	0.88	0.89	0.88	0.69
16	2L1P	0.81	0.88	0.85	0.68
17	2LN3	0.92	0.88	0.90	0.74
18	2HEQ	0.73	0.69	0.71	0.55
19	2KK8	0.87	0.88	0.88	0.64
20	2KD0	0.83	0.91	0.87	0.67
21	2LML	0.94	0.89	0.91	0.68
22	2K3D	0.85	0.90	0.87	0.68
23	2LK2	0.90	0.88	0.89	0.65
24	MH04	0.85	0.88	0.86	0.60
25	1PQX	0.80	0.86	0.83	0.65
26	2L33	0.88	0.88	0.88	0.74
27	2KZV	0.85	0.87	0.86	0.65
28	2KCT	0.85	0.88	0.87	0.75
29	2MDR	0.80	0.87	0.83	0.66
30	2FB7	0.78	0.81	0.79	0.57
31	2MB0	0.90	0.90	0.90	0.83
32	2L05	0.85	0.88	0.86	0.72
33	2KJR	0.89	0.90	0.89	0.74
34	2M5O	0.84	0.88	0.86	0.68
35	MDM2	0.93	0.87	0.90	0.65
36	2LNA	0.85	0.90	0.87	0.73
37	2LA6	0.89	0.87	0.88	0.72
38	6FIP	0.67	0.79	0.73	0.51
39	2LEA	0.79	0.88	0.83	0.68
40	2LL8	0.88	0.90	0.89	0.72
41	2KPN	0.89	0.87	0.88	0.78
42	2K0M	0.94	0.85	0.89	0.73
43	2K5V	0.94	0.92	0.93	0.81
44	2MQL	0.80	0.89	0.84	0.68
45	2K75	0.91	0.85	0.88	0.69
46	2LTM	0.91	0.87	0.89	0.79
47	2KOB	0.90	0.85	0.87	0.65
48	2KHD	0.92	0.85	0.88	0.64
49	2RN7	0.92	0.67	0.78	0.63
50	2LXU	0.92	0.88	0.90	0.73
51	2KIF	0.88	0.90	0.89	0.76
52	2KBN	0.91	0.88	0.89	0.72
53	2MK2	0.87	0.88	0.87	0.70
54	2K50	0.90	0.84	0.87	0.76
55	2KL5	0.83	0.79	0.81	0.63
56	2LTA	0.86	0.79	0.82	0.70
57	2KIW	0.89	0.85	0.87	0.61
58	2LVB	0.92	0.85	0.89	0.70
59	2LND	0.81	0.85	0.83	0.66
60	1WQU	0.87	0.92	0.89	0.80
61	2KL6	0.94	0.84	0.89	0.80
62	6GT7	0.90	0.84	0.87	0.76
63	2JN8	0.88	0.84	0.86	0.65
64	2K5D	0.87	0.92	0.89	0.74
65	2KD1	0.87	0.86	0.87	0.65
66	2LTL	0.90	0.85	0.87	0.63
67	2KVO	0.85	0.81	0.83	0.61
68	1T0Y	0.87	0.85	0.86	0.76
69	2KCD	0.90	0.79	0.84	0.53
70	2KRT	0.91	0.71	0.80	0.80
71	2LFI	0.78	0.83	0.81	0.53
72	2JQN	0.94	0.84	0.88	0.77
73	2L7Q	0.88	0.82	0.85	0.69
74	2KFP	0.80	0.87	0.83	0.59
75	1SE9	0.82	0.78	0.80	0.59
76	2L3G	0.92	0.86	0.89	0.77
77	2L3B	0.81	0.83	0.82	0.67
78	2LRH	0.80	0.86	0.82	0.63
79	1VEE	0.89	0.91	0.90	0.76
80	2K1G	0.87	0.88	0.88	0.80
81	2KKZ	0.90	0.82	0.86	0.68
82	1VDY	0.89	0.92	0.90	0.82
83	2KKL	0.84	0.88	0.86	0.65
84	2N4B	0.80	0.90	0.85	0.65
85	2L8V	0.86	0.77	0.81	0.62
86	2LGH	0.87	0.90	0.89	0.70
87	2K1S	0.80	0.84	0.82	0.61
88	2M4F	0.83	0.88	0.85	0.66
89	2JXP	0.90	0.82	0.86	0.73
90	2L06	0.89	0.87	0.88	0.72
91	2LAH	0.90	0.79	0.84	0.80
92	2LAK	0.90	0.84	0.87	0.72
93	2L82	0.82	0.83	0.82	0.70
94	2M47	0.89	0.81	0.85	0.69
95	2K3A	0.88	0.77	0.82	0.70
96	2M7U	0.80	0.71	0.75	0.72
97	2B3W	0.81	0.85	0.83	0.68
98	KRAS4B	0.92	0.88	0.90	0.83
99	2G0Q	0.86	0.80	0.83	0.72
100	2LF2	0.84	0.88	0.86	0.68

Calculated by the RPF web server at https://montelionelab.chem.rpi.edu/rpf/ running ASDP version 2.3. All scores are between 0 and 1, with higher values indicating better agreement between data and structure. Recall measures the fraction of NOE cross peaks that are retrieved by the query structures, while Precision measures the fraction of retrieved proton pair interactions in the query structure that are relevant, weighted by interproton distance. The F-measure score provides an assessment of the overall fit between the query model structure(s) and the experimental data, assuming that the input data are near complete; the Discriminating Power DP-score measures how the query structure is distinguished from a freely rotating chain model.[32] Scores depend on the NOESY peak lists, chemical shift assignments, and structures.

Supplementary Table 8 Consensus structure bundles. NMR structure bundles calculated by ARTINA and by using consensus distance restraints with the *multnoeassign* command of CYANA. RMSD values are the average of the 20 backbone RMSDs between the individual conformers and their mean coordinates for the residue given in Supplementary Table 4.

ID	Protein	ARTINA		Consensus	
		Target function value ($Å^2$)	RMSD to mean (Å)	Target function value ($Å^2$)	RMSD to mean (Å)
1	6SVC	1.80	0.04	0.61	0.12
2	2JVD	0.30	0.14	0.03	0.33
3	2K57	0.35	0.15	0.29	0.25
4	6SOW	2.32	0.10	0.45	0.40
5	2LX7	0.32	0.80	0.32	0.95
6	2MA6	3.25	0.14	1.95	0.41
7	2JRM	0.50	0.13	0.20	0.37
8	1YEZ	0.58	0.14	0.37	0.35
9	2L9R	0.37	0.21	0.36	0.30
10	2K52	2.68	0.17	0.84	0.48
11	2KRS	0.35	0.18	0.21	0.40
12	2K53	0.60	0.38	0.42	0.33
13	2JT1	0.11	0.29	0.10	0.35
14	2JVO	10.44	0.05	3.66	0.16
15	2ERR	13.61	0.46	8.86	0.62
16	2L1P	2.10	0.08	0.27	0.25
17	2LN3	1.15	0.14	0.09	0.27
18	2HEQ	3.20	0.03	0.82	0.30
19	2KK8	2.24	0.29	0.82	0.78
20	2KD0	4.43	0.07	1.63	0.24
21	2LML	0.67	0.48	0.57	0.69
22	2K3D	2.00	0.12	0.94	0.44
23	2LK2	0.94	0.36	0.45	0.43
24	MH04	0.86	0.46	0.54	0.81
25	1PQX	10.18	0.13	8.54	0.29
26	2L33	1.37	0.16	0.16	0.30
27	2KZV	0.41	0.46	0.30	0.81
28	2KCT	4.45	0.11	2.67	0.21
29	2MDR	3.06	0.12	0.86	0.42
30	2FB7	1.51	0.29	0.37	0.69
31	2MB0	9.63	0.09	4.22	0.25
32	2L05	2.07	0.17	0.53	0.53
33	2KJR	1.04	0.15	0.39	0.45
34	2M5O	7.18	0.07	3.54	0.24
35	MDM2	1.29	0.72	0.63	1.07
36	2LNA	4.06	0.14	1.06	0.61
37	2LA6	6.62	0.11	4.89	0.19
38	6FIP	17.06	0.13	6.79	1.39
39	2LEA	7.98	0.09	1.13	0.35
40	2LL8	2.64	0.24	1.08	0.44
41	2KPN	2.33	0.14	0.72	0.56
42	2K0M	0.69	0.43	0.53	0.57
43	2K5V	1.10	0.22	0.48	0.40
44	2MQL	22.17	0.09	5.11	0.18
45	2K75	0.77	0.41	0.21	0.65
46	2LTM	2.68	0.07	0.96	0.25
47	2KOB	2.66	0.33	1.16	0.55
48	2KHD	0.04	0.48	0.01	0.73
49	2RN7	1.80	1.02	0.61	0.12
50	2LXU	0.30	0.27	0.03	0.33
51	2KIF	3.64	0.14	0.68	0.41
52	2KBN	0.45	0.22	1.58	0.42
53	2MK2	2.62	0.39	0.21	0.71
54	2K50	0.86	0.25	1.04	0.40
55	2KL5	5.90	0.44	0.26	1.12
56	2LTA	0.73	0.46	1.96	0.63
57	2KIW	1.10	0.28	0.37	0.63
58	2LVB	0.86	0.50	0.41	0.52
59	2LND	1.29	0.15	1.12	0.62
60	1WQU	9.51	0.14	0.30	0.30
61	2KL6	1.99	0.17	2.90	0.56
62	6GT7	4.21	0.13	0.63	0.36
63	2JN8	1.12	0.39	1.73	0.72
64	2K5D	1.44	0.19	0.38	0.49
65	2KD1	6.35	0.26	0.46	0.63
66	2LTL	1.07	0.77	1.99	2.42
67	2KVO	3.88	0.22	0.66	0.75
68	1T0Y	1.60	0.15	1.73	0.46
69	2KCD	10.14	0.57	0.24	1.50
70	2KRT	0.83	0.58	5.44	0.90
71	2LFI	0.80	0.73	0.30	2.02
72	2JQN	1.09	0.58	0.43	0.72
73	2L7Q	1.74	0.23	1.02	0.55
74	2KFP	8.46	0.23	0.26	0.57
75	1SE9	1.85	0.48	4.15	1.05
76	2L3G	2.34	0.21	0.70	0.63
77	2L3B	0.81	0.30	0.76	0.55
78	2LRH	5.26	0.18	0.32	0.80
79	1VEE	5.14	0.13	0.76	0.26
80	2K1G	14.38	0.12	1.90	0.29
81	2KKZ	2.36	0.35	3.50	0.86
82	1VDY	9.13	0.13	0.69	0.47
83	2KKL	2.09	0.42	4.31	0.89
84	2N4B	10.49	0.10	0.44	0.38
85	2L8V	2.82	0.80	5.82	1.27
86	2LGH	3.18	0.56	1.06	0.83
87	2K1S	2.63	0.26	1.37	0.72
88	2M4F	6.07	0.18	0.59	0.54
89	2JXP	0.91	1.28	0.86	4.17
90	2L06	13.42	0.25	0.31	0.51
91	2LAH	10.34	0.35	5.11	0.89
92	2LAK	4.21	0.38	6.29	0.81
93	2L82	3.72	0.55	2.02	0.86
94	2M47	1.46	1.59	0.56	2.39
95	2K3A	0.81	0.25	0.78	0.45
96	2M7U	15.64	0.25	0.42	0.93
97	2B3W	23.41	0.33	7.67	0.89
98	KRAS4B	10.89	0.22	7.11	0.65
99	2G0Q	4.53	0.55	1.86	1.25
100	2LF2	4.30	0.38	1.13	0.82

Supplementary Table 9 Results of restrained energy refinement. CYANA structure bundles calculated by ARTINA were energy-refined in explicit water using OPALp. Backbone RMSDs to the reference PDB structure before and after energy refinement show a standard deviation of 0.07 Å, maximal deviation of 0.16 Å, and linear correlation coefficient of 0.998.

ID	Protein	CYANA		OPALp	
		AMBER energy (kcal/mol)	RMSD to reference (Å)	AMBER energy (kcal/mol)	RMSD to reference (Å)
1	6SVC	-664	0.83	-974	0.91
2	2JVD	-1590	0.71	-2044	0.59
3	2K57	-1547	0.71	-2270	0.61
4	6SOW	-1892	1.16	-2369	1.09
5	2LX7	-1981	1.41	-2372	1.37
6	2MA6	-1200	1.56	-1740	1.40
7	2JRM	-1733	1.43	-2309	1.38
8	1YEZ	-1961	0.73	-2463	0.61
9	2L9R	-1319	0.59	-1877	0.54
10	2K52	-2597	1.10	-3242	1.06
11	2KRS	-2228	1.26	-2809	1.19
12	2K53	-2055	0.88	-2753	0.87
13	2JT1	-2064	0.94	-2792	0.87
14	2JVO	-1912	1.77	-2573	1.77
15	2ERR	-2788	2.09	-3397	2.04
16	2L1P	-2259	2.13	-2932	2.14
17	2LN3	-2501	0.89	-3284	0.82
18	2HEQ	-1952	0.60	-2780	0.57
19	2KK8	-2369	1.25	-3099	1.20
20	2KD0	-1865	1.37	-2553	1.29
21	2LML	-2331	1.53	-3095	1.44
22	2K3D	-2347	1.44	-3094	1.43
23	2LK2	-2288	1.42	-3056	1.37
24	MH04	-2942	1.57	-3645	1.56
25	1PQX	-2398	1.40	-3198	1.31
26	2L33	-2613	0.79	-3353	0.80
27	2KZV	-2599	2.62	-3276	2.60
28	2KCT	-2484	0.77	-3225	0.73
29	2MDR	-2770	1.72	-3540	1.74
30	2FB7	-2359	1.94	-3153	1.89
31	2MB0	-2934	1.11	-3905	1.03
32	2L05	-2134	0.74	-3053	0.69
33	2KJR	-2797	1.02	-3557	1.03
34	2M5O	-1612	1.08	-2526	1.04
35	MDM2	-2857	1.24	-3644	1.24
36	2LNA	-3227	0.86	-4005	0.76
37	2LA6	-2771	0.81	-3645	0.78
38	6FIP	-2719	2.05	-3637	2.04
39	2LEA	-2738	1.45	-3599	1.38
40	2LL8	-2611	1.42	-3403	1.34
41	2KPN	-2678	0.97	-3623	0.87
42	2K0M	-3002	1.60	-3776	1.52
43	2K5V	-2818	0.94	-3728	0.84
44	2MQL	-1780	0.98	-2762	0.93
45	2K75	-3142	1.65	-4036	1.57
46	2LTM	-3030	0.67	-3870	0.57
47	2KOB	-2974	2.24	-3982	2.19
48	2KHD	-2886	1.87	-3686	1.80
49	2RN7	-3214	1.93	-4240	1.92
50	2LXU	-3344	1.19	-4281	1.08

ID	Protein	ARTINA		OPAlp	
		AMBER energy (kcal/mol)	RMSD to reference (Å)	AMBER energy (kcal/mol)	RMSD to reference (Å)
51	2KIF	-2906	0.89	-3905	0.81
52	2KBN	-3061	0.92	-3974	0.87
53	2MK2	-2936	1.56	-4013	1.49
54	2K50	-3051	1.00	-3976	0.85
55	2KL5	-3039	2.58	-4149	2.56
56	2LTA	-3214	2.39	-4298	2.36
57	2KIW	-2779	1.59	-3733	1.56
58	2LVB	-3533	1.56	-4492	1.52
59	2LND	-3512	0.85	-4359	0.81
60	1WQU	-3058	0.99	-4019	0.89
61	2KL6	-3287	0.87	-4413	0.78
62	6GT7	-3421	1.39	-4474	1.34
63	2JN8	-3285	1.83	-4290	1.77
64	2K5D	-3500	1.47	-4544	1.43
65	2KD1	-2298	1.99	-3492	1.99
66	2LTL	-3266	2.37	-4322	2.38
67	2KVO	-3111	1.87	-4156	1.76
68	1T0Y	-3261	1.27	-4412	1.23
69	2KCD	-2950	3.13	-4099	3.14
70	2KRT	-4018	2.09	-5052	2.04
71	2LFI	-3451	2.42	-4437	2.45
72	2JQN	-3560	1.52	-4547	1.48
73	2L7Q	-3272	1.57	-4451	1.45
74	2KFP	-3419	2.23	-4485	2.15
75	1SE9	-2800	2.10	-4006	2.07
76	2L3G	-4106	1.28	-5120	1.17
77	2L3B	-3838	1.05	-4904	1.01
78	2LRH	-3853	2.30	-5364	2.26
79	1VEE	-3642	1.03	-4911	0.93
80	2K1G	-4044	1.05	-5109	1.02
81	2KKZ	-3854	1.47	-5136	1.38
82	1VDY	-4235	0.95	-5516	0.87
83	2KKL	-3245	1.26	-4606	1.23
84	2N4B	-4485	1.14	-5526	1.07
85	2L8V	-4036	2.79	-5337	2.81
86	2LGH	-3831	2.43	-5001	2.39
87	2K1S	-5004	1.83	-6232	1.74
88	2M4F	-4357	1.11	-5942	1.06
89	2JXP	-4596	2.58	-5895	2.55
90	2L06	-4389	1.57	-5848	1.47
91	2LAH	-4976	1.71	-6375	1.67
92	2LAK	-4201	1.54	-5388	1.43
93	2L82	-5405	3.55	-7021	3.55
94	2M47	-3907	4.72	-5279	4.72
95	2K3A	-4339	0.99	-5567	0.92
96	2M7U	-4846	2.14	-6344	2.12
97	2B3W	-4335	2.67	-5976	2.68
98	KRAS4B	-5147	1.60	-6814	1.64
99	2G0Q	-3999	2.38	-5697	2.36
100	2LF2	-5149	2.68	-6737	2.68

Supplementary Table 10 Chemical shift assignment accuracy of protein core residues. Chemical shift assignment accuracy (%) is reported for core residues of the given amino acid types. The penultimate column (ARO) presents the accuracy calculated for His, Phe, Tyr, and Trp shifts. Core residues are those with a solvent-accessible surface area (SASA) in the protein of up to 20% of the corresponding SASA of the isolated residue. SASAs were calculated with Biopython 1.7.9.

Protein	ALA	ARG	ASN	ASP	CYS	GLU	GLN	GLY	HIS	ILE	LEU	LYS	MET	PHE	PRO	SER	THR	TRP	TYR	VAL	ARO	RMSD
**6SVC**	100.0	-	80.0	-	-	-	-	100.0	-	-	69.2	-	-	-	95.2	100.0	-	100.0	92.3	-	95.6	0.83
**2JVD**	100.0	-	-	-	-	97.0	93.3	-	-	100.0	100.0	94.1	-	-	-	-	-	-	-	-	-	0.71
**2K57**	100.0	-	100.0	100.0	-	100.0	100.0	100.0	-	100.0	100.0	-	-	87.5	100.0	100.0	100.0	-	85.7	100.0	86.7	0.71
**6SOW**	100.0	100.0	91.7	-	-	-	93.3	-	-	95.2	88.6	-	-	78.0	92.3	100.0	-	-	-	-	78.0	1.16
**2LX7**	85.7	-	-	-	100.0	93.9	-	100.0	-	100.0	100.0	100.0	-	71.0	91.7	-	94.7	95.0	-	100.0	80.4	1.41
**2MA6**	100.0	-	-	100.0	100.0	-	-	100.0	92.3	100.0	100.0	-	-	87.5	-	88.2	-	80.0	-	100.0	86.2	1.56
**2JRM**	100.0	-	-	100.0	71.4	-	-	90.0	-	-	100.0	-	-	-	-	100.0	-	95.0	100.0	-	96.3	1.43
**1YEZ**	100.0	-	-	100.0	-	-	-	100.0	-	98.8	92.9	-	-	87.5	100.0	100.0	100.0	-	100.0	97.7	91.3	0.73
**2L9R**	100.0	88.1	100.0	-	-	100.0	97.6	-	-	100.0	97.1	94.1	100.0	84.4	-	-	100.0	85.0	-	95.5	84.6	0.59
**2K52**	100.0	-	95.5	95.8	-	100.0	100.0	91.7	-	88.0	97.6	-	84.6	93.3	100.0	-	100.0	-	100.0	100.0	96.6	1.10
**2KRS**	100.0	100.0	-	100.0	-	100.0	-	97.6	-	100.0	100.0	-	100.0	-	100.0	100.0	-	100.0	100.0	97.7	100.0	1.26
**2K53**	100.0	-	95.0	100.0	100.0	95.0	-	100.0	-	100.0	100.0	100.0	94.4	100.0	-	100.0	100.0	-	100.0	100.0	100.0	0.88
**2JT1**	85.7	93.8	-	100.0	-	-	86.7	100.0	80.0	90.0	95.2	-	-	-	91.7	-	77.8	95.0	100.0	83.3	93.2	0.94
**2JVO**	87.5	84.6	-	-	-	90.0	-	100.0	90.9	84.6	94.9	-	50.0	70.5	27.3	95.2	100.0	-	-	94.1	73.0	1.77
**2ERR**	100.0	81.8	-	71.4	-	90.0	-	88.0	83.3	93.8	94.9	-	100.0	84.2	12.5	100.0	100.0	-	-	92.0	84.0	2.09
**2L1P**	92.9	-	-	-	100.0	-	92.9	50.0	-	92.9	87.1	82.4	-	93.8	-	91.7	100.0	97.5	92.9	100.0	95.7	2.13
**2LN3**	100.0	93.8	-	100.0	-	100.0	-	100.0	-	100.0	93.9	92.2	100.0	81.3	-	82.4	88.9	-	-	100.0	81.3	0.89
**2HEQ**	95.2	-	100.0	100.0	100.0	93.9	-	83.3	83.3	100.0	87.8	88.2	-	75.0	100.0	100.0	100.0	-	74.1	93.9	76.4	0.60
**2KK8**	-	-	100.0	100.0	100.0	81.8	90.5	-	-	95.2	92.8	100.0	90.9	84.6	-	100.0	92.0	-	100.0	96.1	92.6	1.25
**2KD0**	100.0	-	-	-	100.0	-	100.0	100.0	-	100.0	90.4	100.0	-	100.0	95.8	95.8	91.9	-	-	98.2	100.0	1.37
**2LML**	100.0	-	100.0	87.5	-	100.0	-	100.0	85.7	100.0	100.0	100.0	-	83.3	-	100.0	76.0	60.0	100.0	100.0	81.3	1.53
**2K3D**	100.0	-	82.9	100.0	-	-	71.4	100.0	-	94.2	100.0	100.0	100.0	87.1	-	97.0	96.4	-	97.6	93.9	91.1	1.44
**2LK2**	92.9	62.5	-	-	100.0	90.9	-	83.3	100.0	100.0	94.5	100.0	90.9	100.0	100.0	90.6	90.0	91.9	-	100.0	94.5	1.42
**MH04**	100.0	-	100.0	100.0	-	93.9	78.6	100.0	-	98.6	100.0	-	88.5	83.3	94.4	-	77.8	100.0	100.0	96.1	90.8	1.57
**1PQX**	100.0	-	100.0	100.0	-	-	100.0	100.0	80.0	96.4	97.6	94.1	84.6	68.8	-	91.7	94.4	95.0	100.0	96.6	82.9	1.40
**2L33**	100.0	-	95.0	-	-	100.0	-	100.0	-	-	95.4	100.0	79.2	95.3	100.0	-	100.0	-	100.0	100.0	96.4	0.79
**2KZV**	97.1	-	-	100.0	-	100.0	-	91.7	88.9	92.9	85.4	-	88.5	73.9	91.7	88.2	84.2	-	92.9	97.7	79.7	2.62
**2KCT**	100.0	87.5	-	100.0	-	100.0	-	97.2	-	100.0	94.0	-	-	100.0	100.0	-	95.6	-	100.0	98.7	100.0	0.77
**2MDR**	96.3	-	-	100.0	-	100.0	53.8	96.0	-	100.0	92.3	100.0	-	79.5	86.4	84.6	-	-	92.3	100.0	85.5	1.72
**2FB7**	100.0	-	-	100.0	-	-	-	91.7	-	90.6	90.0	92.6	-	70.0	-	93.8	94.3	-	100.0	100.0	85.0	1.94
**2MB0**	97.2	-	100.0	89.5	-	100.0	100.0	96.0	-	97.4	100.0	100.0	91.7	80.8	-	100.0	100.0	-	100.0	97.4	83.3	1.11
**2L05**	100.0	93.8	-	100.0	100.0	90.9	-	100.0	83.3	100.0	97.6	-	-	81.3	100.0	100.0	96.3	100.0	100.0	98.9	91.9	0.74
**2KJR**	100.0	-	91.7	87.5	-	100.0	93.3	100.0	71.4	100.0	92.9	94.1	100.0	75.0	-	100.0	94.7	-	92.9	100.0	79.5	1.02
**2M5O**	-	-	-	100.0	100.0	-	100.0	86.7	-	99.0	90.5	100.0	76.9	81.3	-	87.5	100.0	-	92.9	97.7	86.7	1.08
**MDM2**	100.0	-	100.0	83.3	0.0	-	100.0	100.0	70.0	91.5	97.8	-	100.0	64.0	100.0	100.0	95.8	-	63.1	100.0	64.0	1.24
**2LNA**	-	87.5	91.7	100.0	-	100.0	93.3	100.0	-	100.0	94.6	-	-	72.5	-	-	100.0	86.8	92.9	100.0	80.1	0.86
**2LA6**	100.0	-	73.8	100.0	-	90.9	-	95.8	-	99.0	100.0	-	-	89.5	100.0	95.8	100.0	-	100.0	100.0	90.8	0.81
**6FIP**	100.0	-	-	93.8	-	100.0	-	100.0	-	95.9	85.7	-	84.6	76.6	91.7	87.5	100.0	60.0	69.2	94.5	72.2	2.05
**2LEA**	100.0	63.6	90.0	96.4	-	100.0	-	93.1	-	92.3	93.2	68.8	51.2	96.4	88.9	90.5	97.4	-	-	98.6	96.4	1.45
**2LL8**	100.0	87.5	91.7	100.0	100.0	100.0	-	-	85.7	100.0	99.0	-	-	88.9	100.0	93.8	89.3	70.0	71.4	97.7	82.9	1.42
**2KPN**	96.4	-	-	100.0	-	-	93.3	100.0	91.7	100.0	97.6	-	-	100.0	100.0	-	97.8	-	93.1	99.0	94.7	0.97
**2K0M**	100.0	100.0	100.0	100.0	87.5	100.0	100.0	100.0	90.0	96.4	98.6	94.1	96.2	75.0	-	20.0	-	63.2	92.3	100.0	77.9	1.60
**2K5V**	97.1	-	91.7	100.0	-	100.0	80.0	100.0	-	96.4	100.0	94.1	-	87.5	-	100.0	100.0	-	100.0	100.0	91.3	0.94
**2MQL**	91.7	-	90.0	100.0	71.4	90.0	30.8	90.0	54.5	79.0	80.0	-	85.7	53.6	63.6	76.2	-	-	70.8	89.0	60.3	0.98
**2K75**	100.0	-	91.7	100.0	-	-	86.7	95.8	-	96.0	85.4	-	-	75.0	-	97.2	100.0	-	76.9	99.0	75.9	1.65
**2LTM**	100.0	100.0	100.0	83.3	-	-	100.0	100.0	-	100.0	91.8	98.0	69.6	86.3	95.8	100.0	100.0	85.0	100.0	98.2	87.6	0.67
**2KOB**	100.0	85.7	90.9	100.0	87.5	-	70.0	33.3	37.5	99.2	76.5	88.2	-	59.1	91.7	60.0	88.9	95.0	64.3	100.0	65.8	2.24
**2KHD**	100.0	75.0	-	-	-	100.0	-	100.0	-	100.0	85.7	91.2	-	77.1	-	-	100.0	-	100.0	100.0	85.5	1.87
**2RN7**	100.0	-	-	-	100.0	-	100.0	-	-	100.0	64.3	-	92.3	-	-	93.3	100.0	75.0	-	100.0	75.0	1.93
**2LXU**	100.0	-	83.3	100.0	100.0	98.2	93.3	95.7	-	97.6	98.2	98.0	96.2	81.3	100.0	100.0	100.0	-	-	99.0	81.3	1.19

Supplementary Table 10 continued

Protein	ALA	ARG	ASN	ASP	CYS	GLU	GLN	GLY	HIS	ILE	LEU	LYS	MET	PHE	PRO	SER	THR	TRP	TYR	VAL	ARO	RMSD
**2KIF**	100.0	96.4	90.9	100.0	-	90.0	92.9	100.0	-	91.3	95.2	90.6	-	75.6	100.0	90.5	100.0	97.4	92.3	100.0	87.7	0.89
**2KBN**	95.2	-	97.0	100.0	-	90.9	78.2	93.3	-	97.9	95.9	80.0	-	100.0	83.3	100.0	100.0	90.0	100.0	96.1	96.0	0.92
**2MK2**	100.0	93.8	-	100.0	93.8	100.0	-	94.4	71.4	96.4	95.7	-	-	94.1	100.0	94.1	100.0	100.0	92.9	100.0	91.8	1.56
**2K50**	100.0	85.7	100.0	96.8	-	100.0	-	86.7	85.7	96.4	93.9	100.0	76.9	100.0	79.2	88.9	94.4	90.0	-	100.0	95.1	1.00
**2KL5**	88.9	46.2	21.4	92.9	8.3	95.0	44.4	90.0	-	76.9	71.9	81.3	-	83.0	-	-	16.7	-	80.0	100.0	81.7	2.58
**2LTA**	92.9	-	-	87.5	-	100.0	100.0	-	-	97.4	91.4	87.1	-	100.0	-	68.8	100.0	70.0	100.0	95.5	88.0	2.39
**2KIW**	95.9	92.9	83.3	93.8	-	-	-	-	-	95.2	85.7	-	-	66.1	100.0	95.2	55.6	73.7	100.0	98.5	78.9	1.59
**2LVB**	100.0	-	100.0	100.0	-	90.9	100.0	61.5	-	96.9	92.9	78.8	61.5	89.6	83.3	84.4	100.0	-	92.6	97.0	90.7	1.56
**2LND**	94.3	-	95.2	100.0	-	77.3	89.3	63.2	-	98.0	95.2	-	69.2	87.5	100.0	94.6	94.4	-	97.4	97.0	92.0	0.85
**1WQU**	100.0	87.5	-	100.0	-	95.5	96.4	92.9	100.0	98.2	79.5	-	-	77.1	95.8	93.9	100.0	95.0	100.0	97.7	91.6	0.99
**2KL6**	100.0	75.0	93.5	100.0	-	100.0	-	100.0	100.0	100.0	83.3	94.1	-	96.9	95.8	100.0	93.1	76.9	95.3	99.4	90.5	0.87
**6GT7**	100.0	-	50.0	95.7	100.0	85.0	-	80.0	100.0	94.5	86.0	66.7	90.9	66.7	81.3	91.7	88.5	97.2	97.4	89.9	93.0	1.39
**2JN8**	97.6	93.8	100.0	75.0	-	100.0	100.0	100.0	55.6	100.0	97.1	94.1	38.5	87.5	100.0	75.0	100.0	97.5	100.0	86.4	87.5	1.83
**2K5D**	100.0	75.0	-	100.0	-	100.0	93.3	-	-	100.0	100.0	88.2	-	84.4	-	96.9	100.0	95.0	96.6	98.2	91.4	1.47
**2KD1**	100.0	93.3	81.8	-	-	90.9	-	93.3	90.9	96.8	95.8	100.0	84.6	75.0	-	84.4	100.0	85.0	79.2	97.7	82.0	1.99
**2LTL**	91.7	-	95.0	74.3	100.0	-	90.4	100.0	-	99.4	93.1	96.0	95.8	75.6	-	92.9	90.6	64.7	-	100.0	72.6	2.37
**2KVO**	100.0	-	100.0	87.5	-	100.0	71.4	96.7	-	100.0	88.1	88.2	89.2	75.7	97.2	97.5	94.7	90.0	100.0	98.5	83.9	1.87
**1T0Y**	100.0	-	100.0	100.0	-	90.0	100.0	100.0	100.0	97.6	89.1	94.1	100.0	92.9	-	92.3	100.0	-	97.6	96.8	97.1	1.27
**2KCD**	100.0	-	73.9	100.0	-	100.0	18.2	94.1	75.0	99.0	81.9	50.0	100.0	81.0	79.2	100.0	100.0	40.0	67.5	100.0	68.2	3.13
**2KRT**	85.7	-	85.7	87.5	-	81.8	78.6	100.0	-	93.6	77.9	90.0	100.0	90.9	25.0	100.0	100.0	-	100.0	100.0	93.5	2.09
**2LFI**	95.2	-	91.7	81.3	-	90.5	38.0	91.3	60.0	84.3	90.2	-	-	58.5	-	81.3	96.3	85.3	69.2	98.5	68.7	2.42
**2JQN**	96.4	-	-	100.0	-	100.0	76.7	95.5	90.0	100.0	87.9	94.1	-	82.5	91.7	95.8	100.0	100.0	100.0	100.0	90.4	1.52
**2L7Q**	100.0	50.0	83.3	91.7	-	95.5	70.5	100.0	-	94.6	90.2	-	100.0	85.2	91.7	91.4	94.7	60.0	88.8	100.0	84.3	1.57
**2KFP**	91.9	-	91.7	100.0	-	-	-	96.7	100.0	98.4	85.5	81.8	95.8	87.5	52.2	100.0	79.3	70.3	90.9	96.9	84.8	2.23
**1SE9**	100.0	100.0	-	100.0	-	100.0	100.0	100.0	-	82.1	94.3	74.4	90.9	75.0	75.0	93.3	100.0	100.0	100.0	92.4	87.9	2.10
**2L3G**	100.0	-	95.5	93.8	92.3	93.9	100.0	96.7	-	92.7	95.6	-	-	89.7	100.0	96.9	97.3	90.0	100.0	96.6	91.3	1.28
**2L3B**	100.0	82.4	-	100.0	100.0	90.9	75.0	100.0	-	95.2	94.7	-	100.0	89.6	83.3	79.2	93.3	-	93.5	100.0	91.6	1.05
**2LRH**	85.7	-	93.9	91.7	-	87.7	96.4	-	-	96.4	90.6	89.7	89.7	64.1	-	88.9	90.0	-	100.0	88.3	78.3	2.30
**1VEE**	100.0	77.8	100.0	100.0	-	-	100.0	95.8	-	100.0	95.7	94.1	-	88.7	89.2	89.6	96.4	100.0	98.2	100.0	94.9	1.03
**2K1G**	100.0	89.3	91.7	93.8	87.5	100.0	83.6	98.1	79.2	100.0	100.0	-	84.6	84.4	100.0	97.9	100.0	100.0	96.2	100.0	90.2	1.05
**2KKZ**	95.2	44.4	94.3	100.0	100.0	87.9	-	90.0	76.9	98.0	92.8	88.2	77.8	83.3	79.2	91.3	100.0	65.0	-	100.0	77.8	1.47
**1VDY**	100.0	89.5	100.0	100.0	100.0	90.9	100.0	66.7	-	97.3	84.9	92.6	92.3	88.6	100.0	89.5	89.7	-	96.4	100.0	91.7	0.95
**2KKL**	89.3	-	91.7	84.4	-	45.5	50.0	91.2	28.6	96.4	91.2	-	-	84.2	-	90.9	89.3	-	-	93.2	75.6	1.26
**2N4B**	100.0	88.2	87.5	100.0	-	97.0	93.3	100.0	80.0	100.0	100.0	-	100.0	82.3	100.0	100.0	95.7	80.0	90.5	97.0	83.0	1.14
**2L8V**	95.1	71.4	41.7	93.8	-	86.4	50.0	100.0	80.0	91.4	76.1	70.6	-	50.0	91.7	78.8	94.7	-	82.0	97.2	67.9	2.79
**2LGH**	98.4	88.2	55.9	100.0	100.0	100.0	92.0	100.0	91.7	97.6	96.3	100.0	100.0	82.5	100.0	100.0	100.0	79.1	100.0	98.9	82.8	2.43
**2K1S**	100.0	93.8	95.2	87.5	-	100.0	100.0	95.7	-	97.1	92.9	-	55.3	100.0	100.0	95.7	95.5	-	78.3	99.2	87.2	1.83
**2M4F**	100.0	-	100.0	100.0	-	92.1	-	100.0	-	100.0	97.3	97.1	92.3	70.1	-	87.5	100.0	75.0	72.7	100.0	71.8	1.11
**2JXP**	100.0	37.5	-	100.0	-	94.5	90.6	100.0	-	98.2	89.6	100.0	80.0	95.1	-	93.8	100.0	-	96.4	99.1	95.7	2.58
**2L06**	98.6	43.8	91.7	100.0	-	93.2	91.7	100.0	80.0	98.9	93.4	64.7	84.6	81.4	72.2	84.8	100.0	-	76.2	100.0	79.2	1.57
**2LAH**	97.2	92.3	97.5	100.0	87.5	90.0	84.6	80.0	78.6	93.6	90.5	100.0	75.0	74.3	90.9	81.8	93.8	94.3	92.3	96.7	84.2	1.71
**2LAK**	100.0	75.0	45.5	93.3	100.0	90.5	81.8	90.5	78.6	96.4	96.7	-	92.3	37.5	88.9	95.1	94.7	66.7	67.9	96.5	63.9	1.54
**2L82**	100.0	74.1	100.0	95.9	-	76.0	98.1	100.0	-	96.9	92.3	-	-	77.5	100.0	78.3	95.8	89.5	97.4	87.4	83.8	3.55
**2M47**	97.6	-	100.0	87.5	100.0	54.5	76.9	90.2	-	81.1	78.3	100.0	76.9	83.3	78.9	89.6	92.9	62.9	91.7	96.6	75.7	4.72
**2K3A**	100.0	-	97.0	-	87.5	95.5	85.7	90.0	57.1	98.2	100.0	-	100.0	-	83.3	94.6	100.0	91.7	93.5	100.0	87.3	0.99
**2M7U**	89.3	61.1	75.0	73.9	50.0	84.2	85.7	96.2	68.2	88.3	75.8	-	83.3	89.5	63.6	65.2	84.8	83.8	91.7	87.2	85.0	2.14
**2B3W**	92.8	91.1	73.9	95.5	-	75.8	75.9	82.1	80.8	100.0	84.4	79.4	72.2	77.2	95.8	87.5	81.4	71.8	53.8	96.0	71.6	2.67
**KRAS4B**	94.4	77.8	80.0	97.1	95.2	84.6	78.4	94.3	100.0	79.1	84.0	63.2	90.0	81.4	68.4	64.3	86.2	-	92.6	95.7	87.7	1.60
**2G0Q**	100.0	87.0	100.0	93.3	75.0	69.7	85.7	95.2	100.0	90.0	82.5	92.3	83.9	87.5	78.6	90.9	94.6	65.0	82.9	94.1	85.0	2.38
**2LF2**	100.0	87.5	83.3	100.0	87.5	86.4	93.3	95.1	100.0	99.3	89.7	-	87.5	89.7	100.0	92.0	91.8	61.2	96.2	100.0	82.4	2.68
**Mean**	97.7	81.8	89.2	95.7	88.4	92.8	85.4	93.6	81.3	96.1	91.6	90.3	86.6	82.2	88.3	91.0	94.2	83.6	91.4	97.5	85.2	1.55
**Median**	100.0	87.5	93.5	100.0	100.0	95.0	92.4	96.7	83.3	97.6	93.1	94.1	90.0	83.3	94.8	93.8	96.4	88.2	96.2	98.6	85.5	1.44

Supplementary Table 11 Quantitative analysis of sources of backbone RMSD in automatically determined structures. Each row presents backbone the RMSD to reference [Å] of an automatically determined protein structure. The quantity was calculated for 7 different residue ranges. The broadest one (**REF**) has been determined by CYRANGE[27] and corresponds to the values reported in Supplementary Table 4 and Figure 4. Remaining residue ranges cover the same fragments of the sequence as REF with the exclusion of: (**A**) the first secondary structure element, (**B**) the last secondary structure element, (**C**) α-helices, (**D**) β-sheets, (**E**) α-helices and β-sheets, and (**F**) flexible loops. The last column (**G**) presents difference between REF and the minimum value of A–F. Proteins that benefit by more than 0.5 Å RMSD decrease from the exclusion of one group of residues are highlighted in green. If a residue range contains less than 10 residues, its RMSD is not calculated.

ID	Protein	REF	A	B	C	D	E	F	G
1	6SVC	0.83	0.84	0.78	0.83	-	-	0.54	0.29
2	2JVD	0.71	0.58	0.68	-	0.71	-	0.69	0.13
3	2K57	0.71	0.74	0.69	0.71	0.66	0.68	0.60	0.11
4	6SOW	1.16	0.57	1.20	-	1.16	-	1.20	0.59
5	2LX7	1.41	1.43	1.43	1.41	1.59	1.59	0.70	0.71
6	2MA6	1.56	1.56	1.51	1.54	1.44	1.37	1.38	0.19
7	2JRM	1.43	1.46	1.58	1.87	1.43	-	1.04	0.39
8	1YEZ	0.73	0.70	0.74	0.73	0.86	0.86	0.63	0.10
9	2L9R	0.59	0.56	0.60	-	0.59	-	0.6	0.03
10	2K52	1.10	1.16	1.13	1.10	1.35	1.37	0.81	0.29
11	2KRS	1.26	1.28	1.22	1.26	1.43	1.40	1.16	0.10
12	2K53	0.88	0.88	0.92	0.80	0.88	-	0.85	0.08
13	2JT1	0.94	0.86	0.95	0.74	0.98	-	0.84	0.20
14	2JVO	1.77	1.76	1.73	1.83	1.63	1.73	1.67	0.14
15	2ERR	2.09	2.13	2.14	2.27	1.97	2.44	1.89	0.20
16	2L1P	2.13	2.25	2.07	2.96	2.13	2.96	1.5	0.63
17	2LN3	0.89	0.87	0.91	0.79	0.92	0.84	0.85	0.10
18	2HEQ	0.60	0.62	0.61	0.60	0.76	0.63	0.43	0.17
19	2KK8	1.25	1.23	1.26	1.28	1.16	1.39	1.08	0.17
20	2KD0	1.37	1.42	1.40	1.15	1.57	1.37	1.30	0.22
21	2LML	1.53	1.46	1.50	1.39	1.53	1.39	1.57	0.14
22	2K3D	1.44	1.28	1.44	1.32	1.54	1.49	1.33	0.16
23	2LK2	1.42	1.49	1.36	1.21	1.42	1.21	1.13	0.29
24	MH04	1.57	1.57	1.58	1.58	1.85	1.97	0.97	0.60
25	1PQX	1.40	1.44	1.39	1.24	1.61	1.62	1.19	0.21
26	2L33	0.79	0.72	0.77	0.70	0.90	-	0.70	0.09
27	2KZV	2.62	2.82	2.70	3.07	2.84	3.33	1.98	0.64
28	2KCT	0.77	0.71	0.78	0.77	0.79	0.79	0.65	0.12
29	2MDR	1.72	1.73	1.70	1.77	1.79	1.96	1.13	0.59
30	2FB7	1.94	2.02	2.01	1.94	2.5	2.5	1.41	0.53
31	2MB0	1.11	1.14	1.11	1.04	1.18	1.06	1.08	0.07
32	2L05	0.74	0.76	0.73	0.75	0.66	0.69	0.73	0.08
33	2KJR	1.02	1.05	1.02	1.06	1.08	1.26	0.83	0.19
34	2M5O	1.08	1.12	1.08	1.02	1.13	1.26	0.93	0.15
35	MDM2	1.24	1.22	1.19	1.17	1.26	1.20	1.08	0.16
36	2LNA	0.86	0.86	0.84	0.85	0.92	1.00	0.77	0.09
37	2LA6	0.81	0.82	0.81	0.81	0.87	0.91	0.56	0.25
38	6FIP	2.05	2.07	2.08	1.98	2.37	2.29	1.72	0.33
39	2LEA	1.45	1.36	1.47	1.51	1.36	1.42	1.34	0.11
40	2LL8	1.42	1.42	1.50	1.47	1.42	1.47	1.32	0.10

Supplementary Table 11 continued

ID	Protein	REF	A	B	C	D	E	F	G
41	2KPN	0.97	0.98	1.04	0.83	1.19	0.93	0.93	0.14
42	2K0M	1.60	1.62	1.57	1.38	1.82	1.45	1.52	0.22
43	2K5V	0.94	0.95	0.85	0.94	1.18	0.93	0.92	0.09
44	2MQL	0.98	1.01	0.98	0.96	1.10	1.14	0.82	0.16
45	2K75	1.65	1.65	1.64	1.65	1.73	1.76	1.47	0.18
46	2LTM	0.67	0.68	0.65	0.66	0.72	0.69	0.62	0.05
47	2KOB	2.24	2.20	2.28	2.08	2.24	2.23	2.20	0.16
48	2KHD	1.87	1.88	1.13	1.11	1.99	0.83	1.78	1.04
49	2RN7	1.93	1.12	2.12	-	1.93	-	1.81	0.81
50	2LXU	1.19	1.21	1.24	1.29	1.22	1.45	0.96	0.23
51	2KIF	0.89	0.90	0.90	0.98	0.92	1.02	0.79	0.10
52	2KBN	0.92	0.88	0.92	0.92	1.00	0.92	0.91	0.04
53	2MK2	1.56	1.58	1.57	1.57	1.80	1.91	1.11	0.45
54	2K50	1.00	1.00	0.91	1.00	0.90	0.88	1.01	0.12
55	2KL5	2.58	2.63	2.69	2.09	3.25	2.93	2.15	0.49
56	2LTA	2.39	2.47	2.32	2.18	2.65	2.88	1.92	0.47
57	2KIW	1.59	1.63	1.56	1.69	1.59	1.86	1.47	0.12
58	2LVB	1.56	1.57	1.65	1.83	1.56	2.36	1.20	0.36
59	2LND	0.85	0.86	0.82	0.66	0.84	0.69	0.87	0.19
60	1WQU	0.99	0.99	0.99	0.96	1.04	1.04	0.89	0.10
61	2KL6	0.87	0.89	0.88	0.87	1.09	1.09	0.63	0.24
62	6GT7	1.39	1.36	1.29	1.16	1.39	1.16	1.40	0.23
63	2JN8	1.83	1.74	1.87	2.17	1.83	2.22	1.61	0.22
64	2K5D	1.47	1.49	1.54	1.47	1.77	1.77	0.96	0.51
65	2KD1	1.99	1.97	1.89	1.94	1.99	2.04	1.91	0.10
66	2LTL	2.37	2.32	2.10	2.33	2.40	2.72	2.09	0.28
67	2KVO	1.87	1.89	2.00	2.01	2.11	2.64	1.33	0.54
68	1T0Y	1.27	1.29	1.30	1.31	1.43	1.71	0.88	0.39
69	2KCD	3.13	2.98	2.40	2.40	3.61	2.75	3.22	0.73
70	2KRT	2.09	1.92	2.00	1.94	2.41	2.18	1.90	0.19
71	2LFI	2.42	2.34	2.40	2.42	2.64	2.59	2.28	0.14
72	2JQN	1.52	1.55	1.51	1.50	1.65	1.57	1.37	0.15
73	2L7Q	1.57	1.35	1.58	1.57	1.61	1.60	1.53	0.22
74	2KFP	2.23	2.29	2.24	2.21	2.44	2.56	1.71	0.52
75	1SE9	2.10	2.19	2.20	2.19	2.48	2.93	1.10	1.00
76	2L3G	1.28	1.32	1.33	1.72	1.28	1.76	0.84	0.44
77	2L3B	1.05	0.97	1.02	1.05	0.96	0.96	1.07	0.09
78	2LRH	2.3	2.35	1.45	1.50	2.57	1.85	2.25	0.85
79	1VEE	1.03	1.03	1.03	1.02	1.09	1.10	0.89	0.14
80	2K1G	1.05	1.05	1.08	1.06	1.12	1.24	0.92	0.13
81	2KKZ	1.47	1.43	1.46	1.51	1.50	1.72	1.32	0.15
82	1VDY	0.95	0.88	1.01	1.12	0.95	1.20	0.83	0.12
83	2KKL	1.26	1.29	1.28	1.26	1.50	1.50	0.85	0.41
84	2N4B	1.14	1.17	1.13	1.17	1.22	1.40	0.94	0.20
85	2L8V	2.79	2.80	2.79	2.79	2.79	2.80	2.54	0.25
86	2LGH	2.43	2.49	2.58	2.67	2.83	3.57	1.67	0.76
87	2K1S	1.83	1.76	1.87	1.81	2.00	2.29	1.56	0.27
88	2M4F	1.11	1.14	1.13	1.16	1.48	1.93	0.83	0.28
89	2JXP	2.58	2.60	2.46	2.22	3.11	2.63	2.51	0.36
90	2L06	1.57	1.58	1.58	1.74	1.57	1.79	1.43	0.14
91	2LAH	1.71	1.75	1.64	1.90	1.71	1.95	1.63	0.08
92	2LAK	1.54	1.54	1.61	1.33	1.34	1.52	1.53	0.21
93	2L82	3.55	3.61	1.04	1.08	3.97	1.24	3.83	2.51
94	2M47	4.72	4.88	4.79	3.77	4.8	4.33	4.71	0.95
95	2K3A	0.99	0.97	1.00	1.02	1.05	1.14	0.89	0.10
96	2M7U	2.14	2.19	2.18	1.96	2.31	2.27	1.92	0.22
97	2B3W	2.67	2.62	2.60	2.91	2.59	2.77	2.50	0.17
98	KRAS4B	1.60	1.61	1.61	1.57	1.67	1.58	1.56	0.04
99	2G0Q	2.38	2.44	2.48	2.50	3.08	3.67	1.14	1.24
100	2LF2	2.68	2.71	2.24	2.37	3.03	3.00	2.51	0.44

Supplementary Table 12 Quality metrics of 26 protein structures determined without and with 4D CC-NOESY spectra.

Protein	Backbone RMSD to reference [Å]		Heavy-atom RMSD to reference [Å]		Backbone chemical shift assignment accuracy [%]		Side-chain chemical shift assignment accuracy [%]	
	without CCNOESY	with CCNOESY	without CCNOESY	with CCNOESY	without CCNOESY	with CCNOESY	without CCNOESY	with CCNOESY
2B3W	2.25	2.67	3.05	3.37	95.28	93.39	81.41	80.09
2HEQ	0.50	0.60	1.27	1.48	95.30	94.48	84.03	84.63
2K1S	1.49	1.83	1.82	2.05	98.39	98.83	92.14	92.02
2K3D	1.54	1.44	2.33	2.13	98.96	98.96	88.89	89.76
2K75	1.65	1.65	2.03	1.98	98.36	98.77	88.36	88.36
2KBN	1.09	0.92	1.76	1.46	98.18	97.98	89.49	88.49
2KCD	1.77	3.13	2.48	3.59	97.22	91.30	86.86	79.24
2KFP	2.03	2.23	2.65	2.89	97.02	97.39	81.96	79.51
2KIW	2.78	1.59	3.23	2.08	94.96	93.99	79.50	80.94
2KKL	1.80	1.26	2.45	2.09	94.06	94.06	81.67	80.60
2KOB	2.27	2.24	2.77	2.93	91.18	88.18	85.05	82.29
2KRS	1.02	1.26	1.35	1.50	98.47	98.47	96.40	96.18
2KVO	2.24	1.87	2.58	2.18	98.35	98.17	90.27	90.00
2KZV	2.04	2.62	2.68	3.13	96.58	95.84	83.45	81.67
2L3B	1.23	1.05	1.74	1.63	93.79	93.97	86.31	86.19
2L8V	7.49	2.79	7.87	3.35	93.63	93.82	76.32	76.58
2LAK	1.49	1.54	1.82	1.82	92.59	92.15	80.73	78.69
2LF2	3.79	2.68	4.10	3.09	97.80	97.56	86.58	85.86
2LGH	1.54	2.43	1.99	2.70	97.43	98.03	88.44	88.92
2LK2	0.90	1.42	1.66	1.98	96.81	96.08	87.44	86.08
2LML	1.15	1.53	1.54	1.90	97.44	97.44	91.53	91.71
2LNA	0.89	0.86	1.51	1.45	98.14	99.30	91.57	90.78
2LX7	1.42	1.41	1.93	2.02	97.81	98.54	88.59	87.84
2LXU	1.64	1.19	2.33	1.81	99.20	99.40	92.77	91.33
2MA6	1.52	1.56	2.09	2.09	98.59	98.94	89.36	89.36
2N4B	1.26	1.14	2.04	1.95	98.94	98.94	89.81	90.25
**Median**	**1.54**	**1.55**	**2.07**	**2.07**	**97.43**	**97.50**	**87.90**	**87.01**
**Mean**	**1.88**	**1.73**	**2.43**	**2.26**	**96.71**	**96.31**	**86.88**	**86.05**

Rows presenting proteins with more than 1 Å difference in backbone RMSD to reference are highlighted in green (if better without 4D CC-NOESY) or red (if better with 4D CC-NOESY).

Supplementary Table 13 Accuracy of automated chemical shift assignment using all input spectra. Chemical shift assignment accuracy (%) is reported for the 45 proteins in the automated chemical shift assignment experiment (see Results section).

Protein	All shifts	Backbone	Side-chain	ALA	ARG	ASN	ASP	CYS	GLU	GLN	GLY	HIS	ILE	LEU	LYS	MET	PHE	PRO	SER	THR	TRP	TYR	VAL
**2JVD**	95.8	99.6	93.2	100.0	100.0	91.7	-	-	98.7	91.1	100.0	-	100.0	100.0	90.8	100.0	75.0	-	95.8	100.0	-	92.9	100.0
**2K57**	95.9	99.6	93.2	100.0	95.8	100.0	95.8	-	100.0	98.2	100.0	-	100.0	100.0	95.3	-	65.6	100.0	100.0	97.8	-	78.6	100.0
**2LX7**	93.1	98.2	89.6	100.0	93.1	-	100.0	100.0	96.4	90.0	93.2	57.1	100.0	99.1	100.0	80.0	74.5	97.2	100.0	89.5	85.0	100.0	100.0
**1YEZ**	94.7	99.7	91.0	100.0	93.9	-	100.0	-	93.9	85.7	100.0	-	100.0	85.7	90.0	0.0	81.7	97.2	100.0	100.0	-	100.0	97.7
**2K52**	92.5	95.8	90.3	100.0	87.0	97.7	95.0	-	97.7	100.0	96.7	-	91.0	87.4	91.6	91.7	82.6	91.7	100.0	100.0	-	88.1	100.0
**2JT1**	89.2	92.8	86.7	100.0	90.0	87.5	100.0	-	92.5	86.7	100.0	80.0	92.9	85.7	80.9	100.0	-	87.5	75.9	94.4	95.0	92.9	84.1
**2HEQ**	87.5	95.0	82.0	95.0	96.9	97.0	96.8	100.0	95.5	-	85.4	61.3	100.0	91.7	92.9	-	50.0	95.0	87.5	100.0	55.0	71.1	92.7
**2KD0**	95.2	98.6	92.7	100.0	92.9	100.0	100.0	100.0	100.0	97.1	100.0	83.3	100.0	97.8	90.6	87.5	71.9	95.8	95.8	94.5	-	-	100.0
**2LK2**	90.8	97.5	86.1	100.0	83.1	91.7	96.8	100.0	87.0	92.2	83.3	91.3	98.2	89.7	85.0	96.0	90.5	97.9	89.3	96.4	86.5	95.1	100.0
**MH04**	92.9	98.8	88.7	98.6	86.4	100.0	100.0	-	92.4	84.5	100.0	-	97.1	97.1	95.2	80.8	85.7	96.7	90.0	80.0	80.0	96.4	97.7
**2KZV**	87.2	95.4	81.3	97.6	77.1	100.0	100.0	-	95.5	90.5	91.7	67.4	92.9	86.3	86.3	78.6	76.1	91.3	89.3	89.5	71.1	92.9	100.0
**2L05**	94.8	99.5	91.7	100.0	91.7	87.0	100.0	100.0	98.5	93.3	100.0	83.3	100.0	100.0	94.1	93.9	87.5	83.9	100.0	97.2	77.5	100.0	100.0
**6FIP**	88.6	98.5	81.8	100.0	78.1	100.0	90.9	-	100.0	96.4	100.0	0.0	96.9	92.9	86.3	84.6	72.4	95.8	93.8	100.0	70.0	61.5	100.0
**2KPN**	92.2	96.7	88.6	97.1	81.3	63.6	97.2	-	96.0	86.7	92.3	90.9	100.0	95.2	82.4	52.4	81.3	100.0	90.6	98.3	-	89.7	98.7
**2K0M**	91.7	95.3	89.1	94.6	96.4	100.0	97.9	68.8	97.3	100.0	97.6	72.7	97.1	94.0	95.7	92.3	63.0	95.7	89.2	100.0	57.9	87.5	98.7
**2K5V**	94.2	98.9	90.8	97.1	90.6	90.0	100.0	-	91.9	86.7	98.3	-	92.0	93.7	93.6	79.2	85.4	100.0	97.2	100.0	100.0	100.0	100.0
**2KOB**	81.8	87.4	77.8	100.0	72.5	85.3	85.4	87.5	83.1	73.3	66.7	25.0	100.0	84.2	76.2	0.0	56.7	91.7	71.3	88.9	95.0	71.4	100.0
**2KHD**	90.6	96.9	85.6	100.0	73.7	83.3	100.0	100.0	86.7	92.0	97.1	66.7	98.5	90.5	90.2	-	66.2	100.0	96.7	100.0	-	100.0	99.0
**2MK2**	94.0	99.2	90.0	100.0	87.7	100.0	100.0	100.0	98.2	94.0	97.9	76.2	96.4	95.1	100.0	81.8	95.7	97.9	90.4	100.0	80.0	87.5	97.9
**2K50**	92.3	98.0	88.3	100.0	94.3	100.0	100.0	-	96.6	-	96.7	80.6	98.2	91.1	95.1	63.4	87.1	65.0	93.9	91.7	90.0	-	97.7
**2KIW**	85.5	96.3	78.2	95.2	92.8	87.5	93.8	-	92.7	90.0	100.0	67.7	98.4	83.3	69.6	68.0	69.4	75.0	92.5	82.2	68.4	94.0	94.9
**1WQU**	90.5	98.6	84.9	100.0	93.4	100.0	100.0	-	95.5	87.8	95.0	86.1	98.6	80.5	77.9	-	75.0	96.9	95.2	100.0	97.5	95.2	97.9
**2KL6**	94.1	98.1	91.3	100.0	84.4	92.4	100.0	-	93.0	86.7	97.9	83.3	96.4	92.9	97.5	85.7	72.9	100.0	100.0	97.8	89.7	87.7	98.5
**6GT7**	85.1	96.7	78.1	100.0	90.6	58.8	95.5	100.0	76.6	100.0	86.7	90.0	94.2	88.7	77.0	100.0	76.2	79.2	94.4	83.3	64.6	88.2	94.9
**2K5D**	94.8	98.5	92.1	100.0	90.4	89.6	96.6	-	100.0	93.3	97.2	-	100.0	94.4	91.6	96.4	81.3	100.0	95.8	98.4	60.0	96.5	98.0
**2KVO**	91.3	96.7	87.3	100.0	77.0	93.6	92.5	-	96.1	90.5	93.3	75.0	100.0	89.8	90.4	94.0	75.5	79.2	96.9	96.9	90.0	90.5	98.5
**2KCD**	81.2	86.7	77.6	100.0	95.2	75.9	100.0	50.0	77.0	81.2	91.3	67.5	99.2	72.7	83.5	92.0	60.7	83.3	96.9	94.9	55.0	68.0	100.0
**2L7Q**	86.8	94.5	81.5	100.0	64.1	82.3	96.3	-	93.2	69.2	97.9	50.0	96.4	84.5	89.7	68.0	85.5	88.3	93.0	97.3	92.5	82.9	94.5
**2KFP**	86.2	96.8	79.6	97.3	68.2	91.2	100.0	-	89.6	90.5	94.4	81.0	98.4	90.1	75.1	95.8	82.1	76.8	93.8	86.2	86.0	74.3	96.9
**2L3B**	88.8	94.0	85.0	90.5	79.3	61.1	90.9	50.0	81.8	80.6	97.6	86.7	94.6	93.3	93.1	78.6	90.0	88.1	90.3	97.8	-	90.0	98.5
**1VEE**	92.9	98.0	89.1	100.0	93.5	100.0	100.0	-	98.5	100.0	93.4	-	100.0	98.0	84.6	-	76.7	96.5	85.8	95.3	97.5	83.9	100.0
**2K1G**	89.7	95.4	85.6	100.0	92.4	87.0	97.5	25.0	90.9	86.4	92.2	70.8	97.6	85.7	95.8	63.3	72.7	100.0	91.3	98.0	82.5	90.0	100.0
**2KKZ**	89.7	95.2	86.0	91.5	86.9	84.3	100.0	100.0	90.9	88.9	88.7	61.5	98.9	90.3	88.2	67.1	84.0	83.0	93.7	100.0	80.0	100.0	100.0
**1VDY**	90.2	95.5	86.4	100.0	91.9	84.6	100.0	100.0	78.5	100.0	71.7	95.2	99.1	84.3	90.1	84.6	78.9	96.6	87.3	93.1	100.0	92.9	100.0
**2KKL**	82.3	90.2	76.3	95.2	66.4	83.3	76.1	-	84.4	78.0	90.8	44.9	97.6	81.8	96.1	-	66.1	90.1	86.3	89.4	-	78.6	95.1
**2L8V**	82.2	93.1	74.6	97.9	77.8	83.3	98.4	-	83.6	70.0	96.7	88.9	92.9	76.8	74.6	-	65.3	89.4	72.3	97.2	89.5	72.1	84.7
**2LGH**	90.5	96.2	86.1	98.2	79.3	58.8	100.0	100.0	98.3	93.3	97.2	74.1	85.5	100.0	96.0	94.1	80.9	100.0	98.4	97.8	73.8	78.6	100.0
**2M4F**	85.1	91.1	80.4	96.4	100.0	90.7	93.8	-	87.2	46.2	97.6	44.4	93.9	98.2	78.9	76.9	70.3	-	76.8	94.4	70.0	65.4	99.1
**2JXP**	89.5	93.9	86.1	97.6	86.7	100.0	100.0	-	72.7	89.9	82.9	64.3	98.1	89.3	89.6	77.8	87.5	86.1	92.5	100.0	-	88.1	99.2
**2LAK**	84.2	91.7	78.6	93.7	79.0	45.5	100.0	100.0	82.2	92.0	91.5	72.9	100.0	90.2	94.1	77.8	65.4	81.2	92.4	85.5	63.2	62.7	93.6
**2M47**	84.6	91.6	79.0	98.2	83.0	93.8	74.1	100.0	84.7	79.5	91.2	66.7	85.6	89.2	96.1	53.8	86.7	76.1	80.3	92.9	58.1	95.8	85.8
**2K3A**	86.8	94.2	80.0	89.5	87.5	84.8	96.9	87.5	87.9	69.2	83.0	63.6	88.1	90.8	63.3	76.9	87.5	91.7	89.5	88.6	90.0	91.7	98.3
**2B3W**	82.2	89.6	77.4	96.2	79.4	80.7	94.5	-	83.1	82.6	94.1	61.4	98.8	88.6	81.9	74.2	81.1	94.3	83.0	57.7	65.4	52.6	96.5
**KRAS4B**	85.2	97.7	77.5	96.3	76.2	85.0	95.2	85.7	88.5	90.3	96.4	78.4	81.6	80.6	78.7	80.0	74.3	70.7	81.5	85.6	-	89.8	96.0
**2LF2**	89.1	96.6	83.5	96.9	71.4	90.3	98.9	87.5	96.8	93.3	95.4	81.0	98.4	90.6	77.2	85.0	85.8	96.7	93.0	95.4	69.3	88.5	100.0
**Median**	**89.7**	**96.6**	**86.0**	**100.0**	**87.0**	**90.0**	**98.7**	**100.0**	**92.7**	**90.0**	**96.4**	**72.9**	**98.2**	**90.3**	**90.1**	**80.4**	**76.5**	**94.3**	**92.5**	**96.9**	**80.0**	**89.7**	**98.7**

Supplementary Table 14 Accuracy of automated chemical shift assignment using all except NOESY-type input spectra. Chemical shift assignment accuracy (%) is reported for the 45 proteins in the automated chemical shift assignment experiment (see Results section).

Protein	All shifts	Backbone	Side-chain	ALA	ARG	ASN	ASP	CYS	GLU	GLN	GLY	HIS	ILE	LEU	LYS	MET	PHE	PRO	SER	THR	TRP	TYR	VAL
**2JVD**	91.4	100.0	85.4	100.0	100.0	70.8	-	-	100.0	80.0	100.0	-	100.0	100.0	83.2	100.0	66.7	-	95.8	77.8	-	80.0	100.0
**2K57**	92.5	98.9	88.0	100.0	89.6	84.2	95.8	-	100.0	87.7	100.0	-	100.0	100.0	95.3	-	53.1	100.0	100.0	97.8	-	60.7	100.0
**2LX7**	85.8	93.8	80.4	100.0	93.1	-	100.0	87.5	83.6	65.0	84.7	85.7	100.0	99.1	100.0	100.0	57.4	94.4	100.0	89.5	55.0	85.2	100.0
**1YEZ**	92.3	98.7	87.5	100.0	92.7	-	100.0	-	87.8	78.6	100.0	-	100.0	85.7	90.0	100.0	69.0	97.2	95.8	100.0	-	100.0	97.7
**2K52**	85.4	94.1	79.7	85.7	84.8	77.3	85.0	-	96.6	67.9	86.7	-	99.1	69.5	96.4	83.3	63.0	79.2	100.0	100.0	-	61.9	100.0
**2JT1**	75.6	99.1	58.6	100.0	61.7	75.0	100.0	-	79.2	65.0	100.0	80.0	73.8	70.5	63.2	90.9	-	79.2	79.3	88.9	50.0	80.0	78.4
**2HEQ**	75.1	87.0	66.2	87.5	75.0	63.6	87.1	100.0	71.2	-	64.6	35.5	92.9	82.3	76.5	-	66.7	86.7	87.5	88.6	40.0	76.8	92.7
**2KD0**	92.9	97.5	89.5	100.0	92.9	90.9	100.0	100.0	100.0	91.4	90.5	66.7	100.0	92.8	91.2	95.8	62.5	95.8	94.4	94.5	-	-	100.0
**2LK2**	84.0	96.1	75.6	100.0	74.2	68.8	93.5	100.0	83.3	77.8	88.9	84.8	98.2	86.8	80.0	72.0	85.7	97.9	92.9	96.4	67.6	75.6	100.0
**MH04**	87.3	98.6	79.1	94.3	80.0	84.8	100.0	-	97.0	86.9	100.0	-	98.6	82.9	94.0	76.9	63.3	88.3	93.3	88.0	45.0	80.0	98.9
**2KZV**	77.0	91.0	66.9	97.6	63.5	75.8	89.1	-	90.9	52.4	83.3	69.6	89.3	66.1	79.4	92.9	60.9	95.7	81.3	78.9	57.9	78.6	93.2
**2L05**	81.8	95.5	73.1	100.0	82.3	60.9	100.0	100.0	90.9	53.3	86.7	66.7	97.1	87.3	77.3	87.9	75.0	46.2	91.7	100.0	55.0	78.6	100.0
**6FIP**	85.7	99.4	76.2	100.0	75.0	100.0	89.1	-	87.3	91.1	100.0	11.1	91.8	92.9	82.9	85.9	81.5	94.4	100.0	100.0	35.0	88.9	95.5
**2KPN**	87.9	96.3	81.3	97.1	87.5	86.4	95.8	-	87.9	66.7	94.9	68.2	100.0	89.3	75.9	61.9	58.3	89.6	87.5	96.6	-	76.2	97.4
**2K0M**	82.5	91.1	76.3	95.9	77.5	86.4	97.9	56.3	85.1	82.1	95.2	50.0	95.7	81.0	92.2	84.6	47.1	95.7	89.2	100.0	15.8	78.6	96.0
**2K5V**	89.5	98.5	83.1	97.1	81.3	75.0	100.0	-	88.9	66.7	93.3	-	91.1	97.6	90.9	87.5	64.6	100.0	97.2	100.0	60.0	73.2	100.0
**2KOB**	74.5	85.6	66.3	100.0	81.3	50.5	93.8	12.5	80.0	73.3	66.7	31.3	87.8	82.0	75.5	0.0	54.5	70.8	72.5	96.3	30.0	52.5	100.0
**2KHD**	83.0	92.5	75.4	100.0	71.9	69.4	100.0	93.8	80.6	80.0	88.6	62.5	58.2	88.9	89.2	-	54.9	100.0	93.3	100.0	-	69.0	96.0
**2MK2**	85.0	98.7	74.5	100.0	76.2	72.7	97.5	75.0	90.9	73.5	93.8	69.0	89.3	86.9	100.0	72.7	74.5	97.9	78.8	85.2	70.0	75.0	94.8
**2K50**	85.6	95.6	78.4	100.0	87.9	75.8	89.1	-	88.8	-	93.3	58.1	97.3	93.8	93.7	48.8	69.6	48.3	93.9	91.7	40.0	-	100.0
**2KIW**	78.3	95.2	67.0	95.2	90.4	66.7	93.8	-	87.3	70.0	100.0	51.6	92.1	85.7	62.9	64.0	61.3	77.8	83.6	77.8	73.7	76.2	89.9
**1WQU**	83.4	97.9	73.6	100.0	92.1	81.8	100.0	-	87.5	72.4	90.0	81.9	97.1	78.8	64.7	-	50.0	94.8	94.0	100.0	80.0	54.8	91.8
**2KL6**	85.9	95.5	79.2	100.0	53.1	73.7	90.6	-	85.2	66.7	97.9	83.3	98.2	94.6	86.6	95.2	45.8	98.3	100.0	95.6	46.2	67.9	98.0
**6GT7**	61.0	95.4	40.0	90.9	59.4	52.9	81.8	76.9	58.9	50.0	60.0	60.0	62.5	59.4	51.5	63.6	57.1	58.3	83.3	72.2	62.5	52.9	65.3
**2K5D**	88.0	96.4	81.8	100.0	86.5	68.8	90.9	-	96.6	66.7	88.9	-	100.0	86.5	88.2	64.3	68.8	97.2	95.8	100.0	75.0	63.2	98.0
**2KVO**	84.4	95.2	76.5	100.0	62.1	76.6	92.5	-	91.5	69.0	91.7	50.0	100.0	89.8	87.5	68.0	64.3	77.1	93.8	96.9	70.0	57.1	98.5
**2KCD**	76.3	85.6	70.1	100.0	81.0	60.2	88.9	62.5	86.2	61.4	91.3	65.0	99.2	67.4	80.0	100.0	50.0	80.0	93.8	94.9	60.0	61.6	100.0
**2L7Q**	61.1	72.9	52.7	95.2	34.4	49.4	86.3	-	66.7	32.4	91.7	50.0	81.0	64.3	62.9	68.0	43.1	86.7	83.7	78.4	5.0	25.9	96.4
**2KFP**	75.3	91.1	65.4	94.6	56.5	59.6	90.0	-	90.9	64.9	97.2	81.0	98.4	91.4	61.7	87.5	53.6	67.4	87.5	82.8	43.9	50.0	95.3
**2L3B**	68.7	83.1	57.8	90.5	51.2	50.0	78.4	25.0	69.1	46.8	92.9	53.3	94.6	59.3	70.3	71.4	68.6	78.6	83.3	97.8	-	53.0	83.1
**1VEE**	84.4	96.4	75.3	100.0	87.0	77.3	98.6	-	93.9	85.7	86.8	-	100.0	93.4	72.8	-	68.5	91.8	80.0	90.6	55.0	58.9	100.0
**2K1G**	82.6	94.6	74.1	100.0	86.4	72.5	100.0	0.0	83.1	72.7	79.2	70.8	97.6	85.7	92.4	67.3	61.0	100.0	82.6	92.0	40.0	73.8	100.0
**2KKZ**	71.2	83.5	62.8	83.0	76.2	60.2	78.6	100.0	70.2	75.6	79.2	30.8	81.1	61.0	71.6	52.9	59.6	50.0	82.5	79.2	70.0	85.7	97.4
**1VDY**	81.4	91.8	73.8	100.0	65.4	53.8	100.0	100.0	76.9	82.1	63.0	76.2	98.2	87.9	78.7	84.6	47.4	91.5	80.0	93.1	100.0	82.1	99.1
**2KKL**	57.8	69.4	49.0	86.7	32.9	57.1	67.0	-	72.5	49.2	80.3	30.6	50.0	48.3	88.2	-	34.7	64.8	64.4	51.1	-	100.0	64.0
**2L8V**	73.5	89.5	61.9	93.8	65.7	70.0	85.9	-	84.9	50.0	86.7	72.2	78.6	74.0	74.6	-	65.4	74.5	58.5	97.2	50.0	59.3	82.3
**2LGH**	82.0	92.6	73.7	84.7	74.1	50.0	93.1	100.0	89.2	75.2	93.1	67.2	84.3	98.1	94.0	85.3	61.8	100.0	96.9	95.5	47.6	92.9	99.1
**2M4F**	84.5	94.2	76.9	96.4	93.8	72.9	93.8	-	82.0	69.2	96.4	61.1	96.6	95.5	76.8	76.9	69.2	-	89.3	97.8	80.0	62.8	96.4
**2JXP**	79.3	86.7	73.4	96.0	78.8	63.6	80.0	-	54.0	66.1	74.3	75.0	91.6	79.4	84.3	55.6	68.8	86.1	78.8	100.0	-	71.4	95.9
**2LAK**	78.0	89.1	69.7	90.1	69.8	18.2	97.3	100.0	77.8	84.0	89.6	55.7	100.0	93.8	94.1	61.1	53.3	77.9	93.9	89.1	40.4	62.2	76.4
**2M47**	76.4	86.7	68.3	98.2	74.1	77.1	74.1	79.2	77.3	66.7	93.0	50.0	76.0	88.3	94.2	69.2	62.7	55.7	73.2	86.6	39.0	75.0	89.4
**2K3A**	82.8	94.5	72.1	91.0	87.5	73.3	100.0	87.5	83.8	73.1	83.0	70.5	84.5	93.9	95.9	84.6	6.3	91.7	82.4	86.2	75.0	67.8	98.3
**2B3W**	76.6	92.3	66.3	97.1	68.9	68.4	90.9	-	87.7	75.7	92.2	61.4	100.0	85.0	77.8	71.0	57.9	54.3	87.2	73.1	48.7	48.7	96.5
**KRAS4B**	66.9	85.7	55.4	92.6	52.3	55.0	76.2	95.2	73.8	54.0	96.4	70.3	59.2	77.1	55.1	80.0	60.8	31.7	57.4	73.3	-	48.1	89.3
**2LF2**	78.7	89.7	70.5	95.9	64.8	66.7	70.5	25.0	92.5	70.0	92.3	57.1	95.6	86.2	72.4	70.0	70.8	58.3	82.5	92.7	49.1	71.2	97.4
**Median**	**82.5**	**94.2**	**73.7**	**97.6**	**76.2**	**70.0**	**93.3**	**87.5**	**86.2**	**70.0**	**91.7**	**65.0**	**96.6**	**86.5**	**82.9**	**76.9**	**61.6**	**86.7**	**87.5**	**93.1**	**52.5**	**71.4**	**97.4**
