# Supplementary material for: Rapid protein assignments and structures from raw NMR spectra with the deep learning technique ARTINA
Source: Nat Commun. 2022 Oct 18;13:6151. doi: 10.1038/s41467-022-33879-5 (PMC9579175; doi:10.1038/s41467-022-33879-5)
Supplement: Supplementary file 2 — Description of Additional Supplementary files [file 41467_2022_33879_MOESM2_ESM.docx]

**Supplementary Movie 1** Visualization of 100 automatically determined protein structures (blue) along with their PDB depositions (orange). The displayed proteins are sorted by their RMSD to PDB reference in ascending order.

**Supplementary Movie 2** Visualization of automated visual spectrum analysis. The movie presents sequentially all planes from a challenging ^13^C-resolved [^1^H,^1^H]-NOESY spectrum of the 20 kDa protein 2B3W. All planes were annotated automatically by ARTINA within 5 minutes and without any human involvement in the process. Model confidence about a signal being a true peak is presented by color-coding, according to the legend in the upper right corner of the movie.

**Supplementary Movie 3** Visualization of the ability of automated visual spectrum analysis to handle strong background artefacts. The movie presents sequentially all planes from a noisy ^13^C-resolved [^1^H,^1^H]-NOESY spectrum of the 7 kDa protein 6SOW with only 20% ^13^C labeling. All planes were annotated automatically by ARTINA within 5 min and without any human involvement in the process. The model confidence about a signal being a true peak is presented by color-coding, according to the legend in the upper right corner of the movie.

**Supplementary Movie 4** Visualization of automated visual spectrum analysis. The movie presents sequentially all planes from an HCCH-TOCSY spectrum, which has been acquired on the 16 kDa protein 2LGH. All planes have been annotated automatically by ARTINA without any human involvement in the process. The spectrum analysis time was under 5 minutes. The model confidence about a signal being a true peak is presented by color-coding according to the legend in the upper right corner of the movie.

**Supplementary Movie 5** Video tutorial presenting all steps of automated protein structure determination with ARTINA as available on the nmrtist.org website.
